# Supplementary figures and images for: Tumor elimination by clustered microRNAs miR-306 and miR-79 via noncanonical activation of JNK signaling
Source: eLife. 2022 Oct 12;11:e77340. doi: 10.7554/eLife.77340 (PMC9612915; doi:10.7554/eLife.77340)

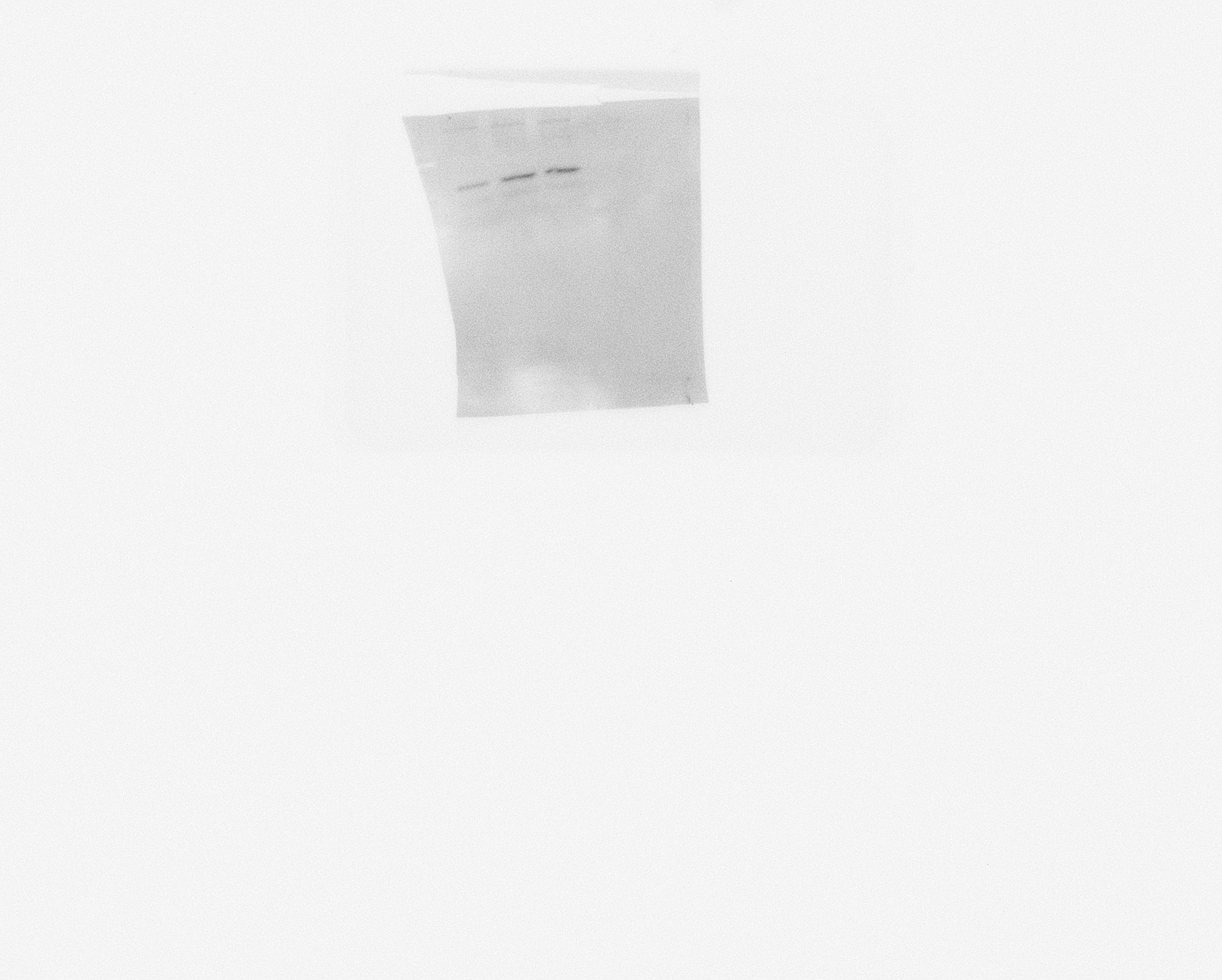

Supplement: Figure 3—figure supplement 2—source data 1. [file elife-77340-fig3-figsupp2-data1.zip › Figure 3—figure supplement 2—source data 1/Figure 3—figure supplement 2G raw data/#1/P-JNK/P-JNK.tif]

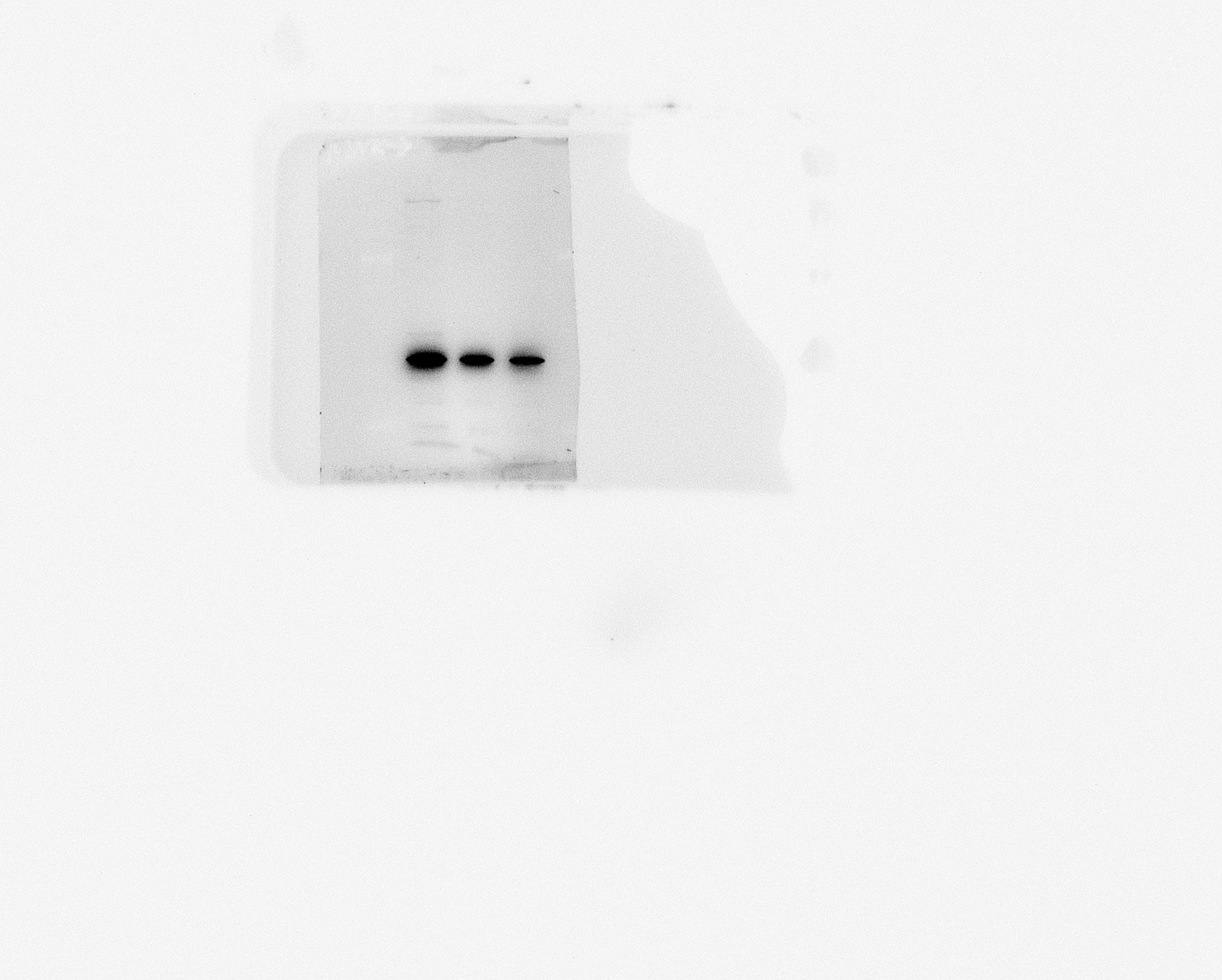

Supplement: Figure 3—figure supplement 2—source data 1. [file elife-77340-fig3-figsupp2-data1.zip › Figure 3—figure supplement 2—source data 1/Figure 3—figure supplement 2G raw data/#1/total JNK/total JNK.tif]

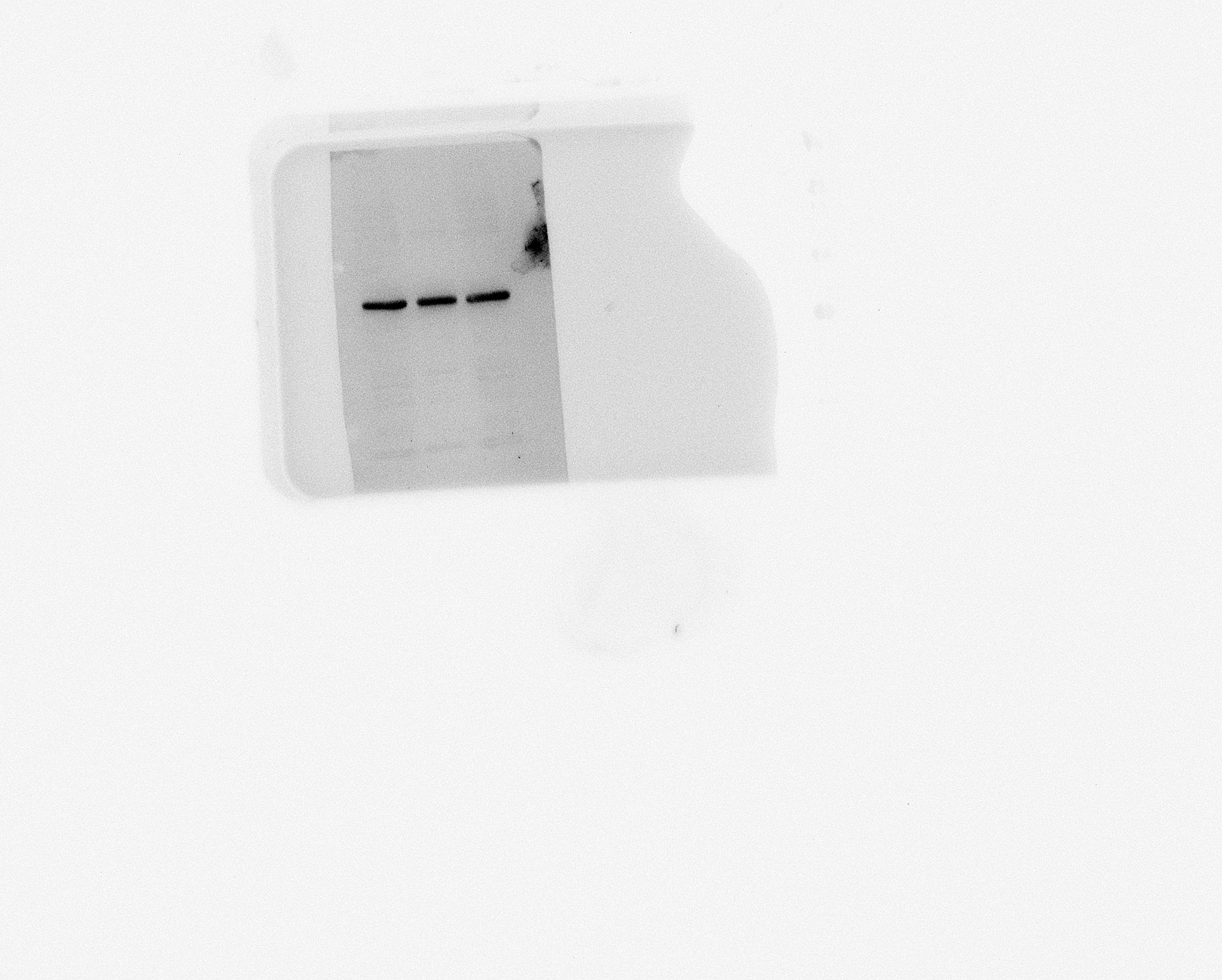

Supplement: Figure 3—figure supplement 2—source data 1. [file elife-77340-fig3-figsupp2-data1.zip › Figure 3—figure supplement 2—source data 1/Figure 3—figure supplement 2G raw data/#1/tubulin/tubulin.tif]

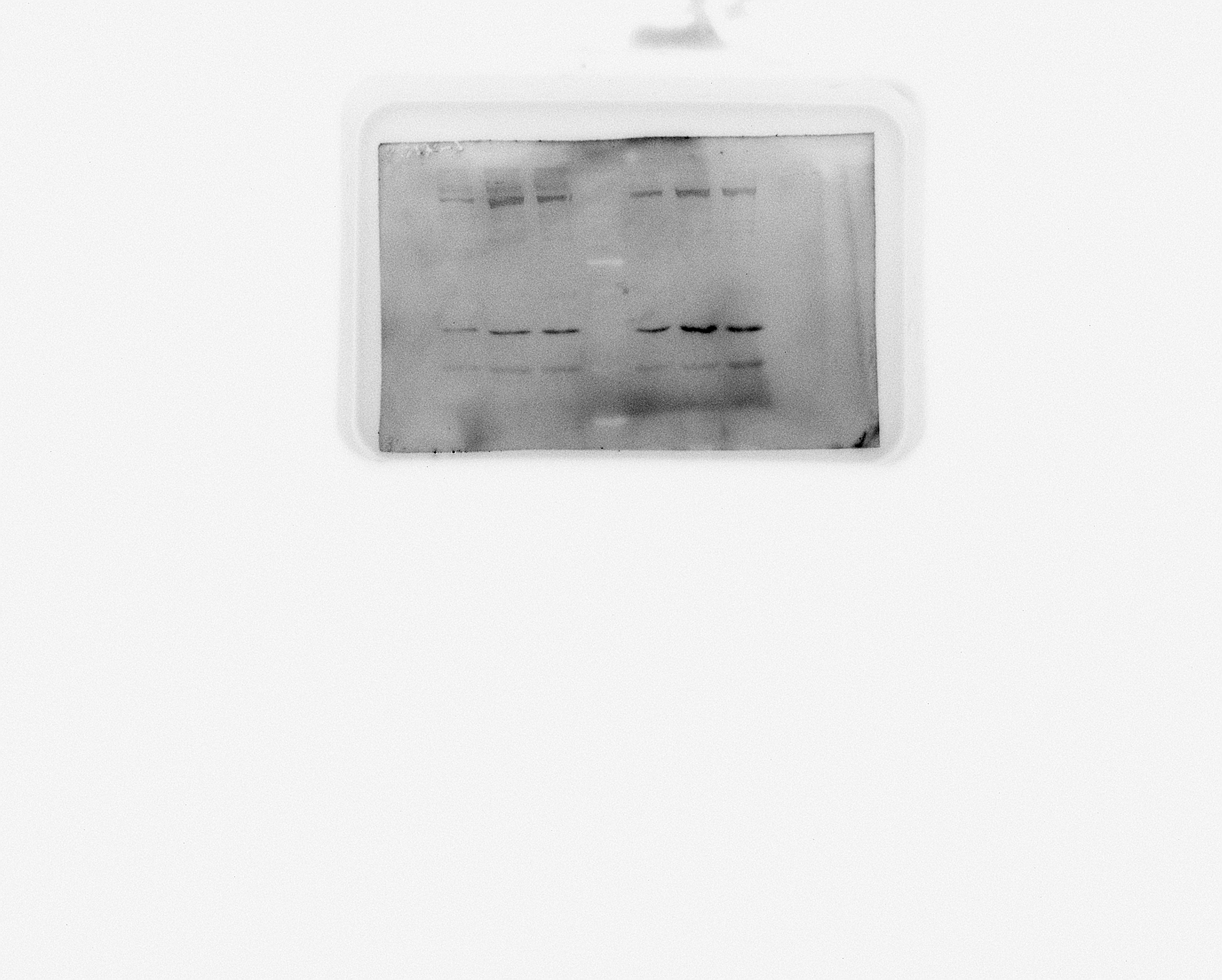

Supplement: Figure 3—figure supplement 2—source data 1. [file elife-77340-fig3-figsupp2-data1.zip › Figure 3—figure supplement 2—source data 1/Figure 3—figure supplement 2G raw data/#2#3/P-JNK/P-JNK.tif]

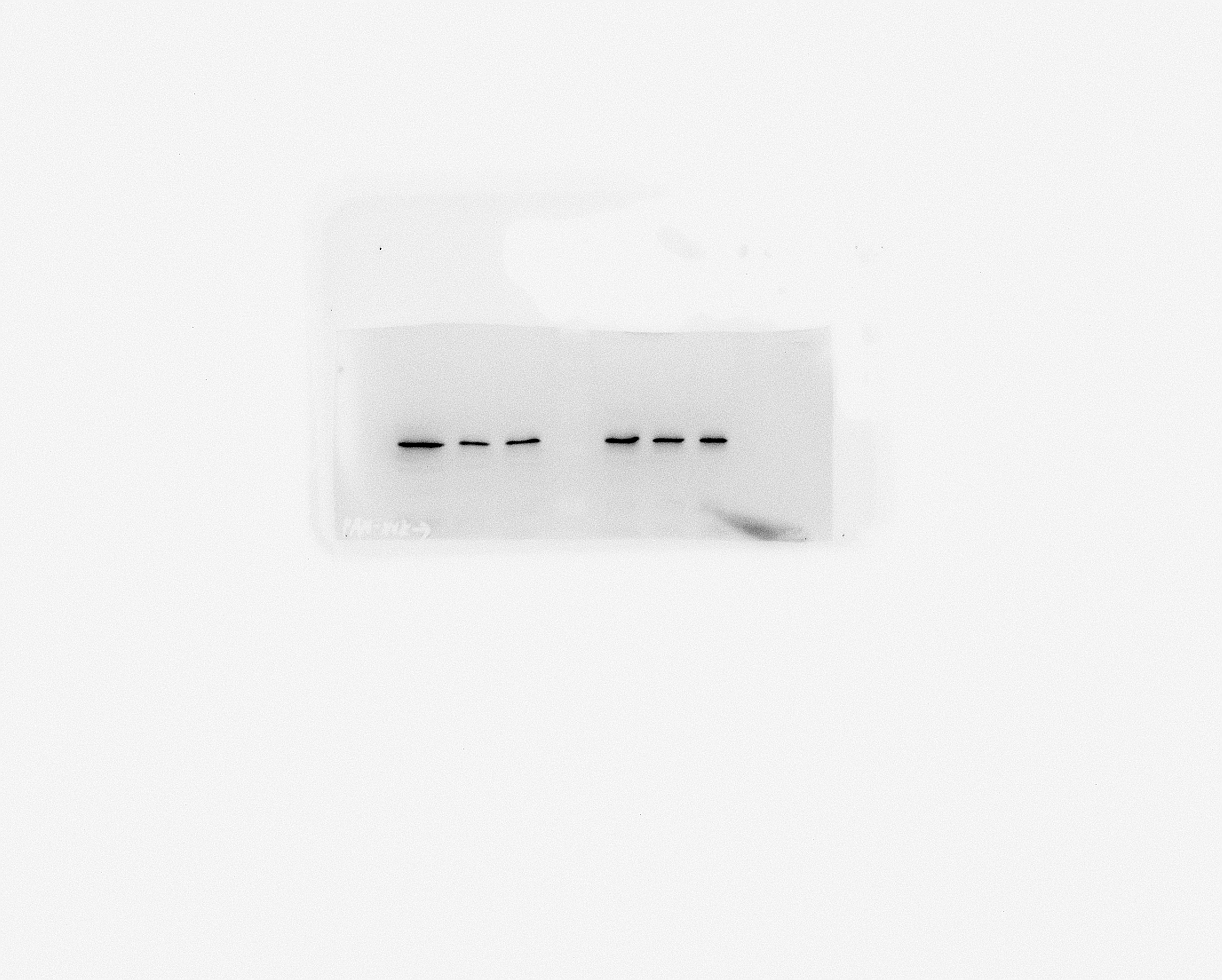

Supplement: Figure 3—figure supplement 2—source data 1. [file elife-77340-fig3-figsupp2-data1.zip › Figure 3—figure supplement 2—source data 1/Figure 3—figure supplement 2G raw data/#2#3/total JNK/total JNK.tif]

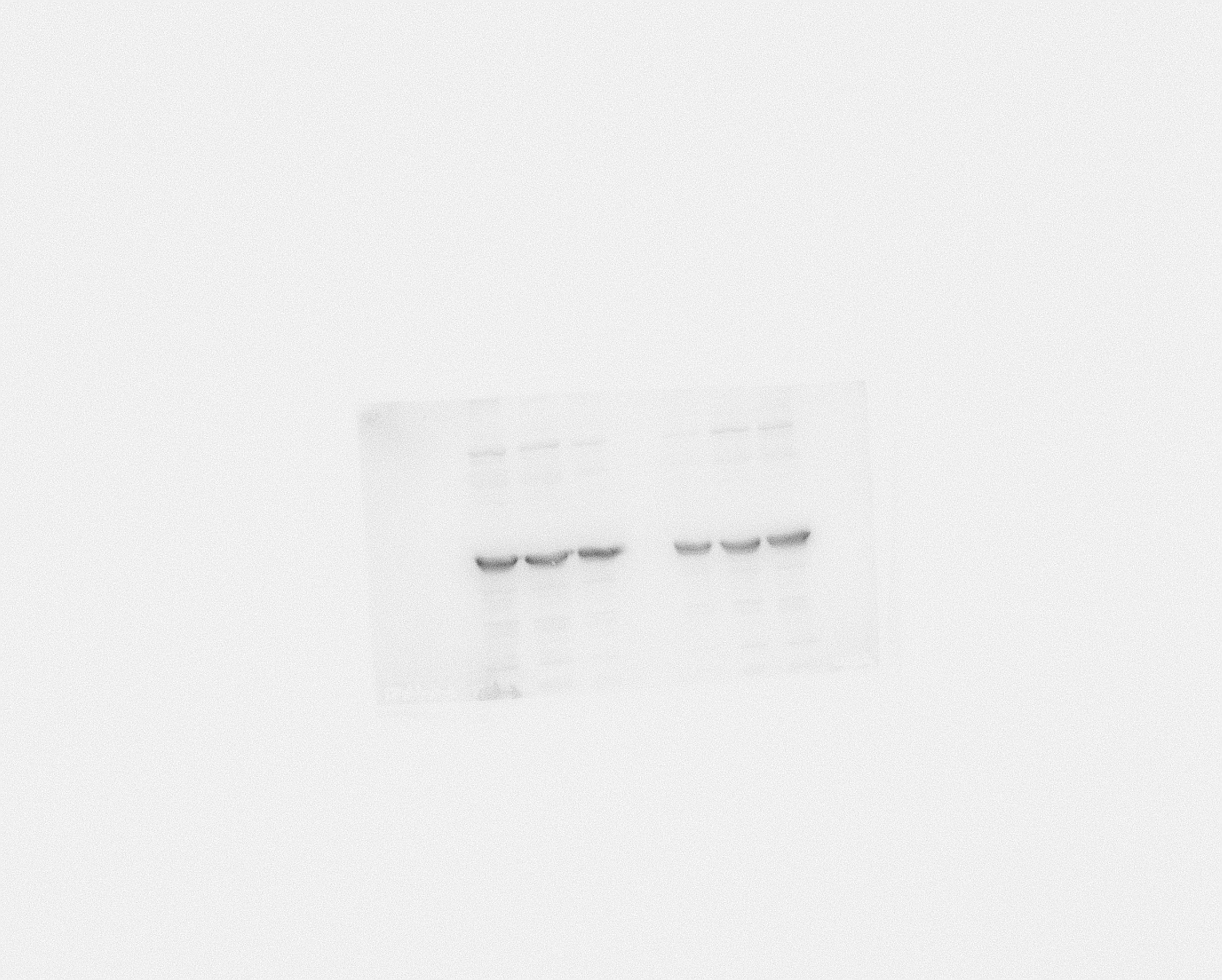

Supplement: Figure 3—figure supplement 2—source data 1. [file elife-77340-fig3-figsupp2-data1.zip › Figure 3—figure supplement 2—source data 1/Figure 3—figure supplement 2G raw data/#2#3/tubulin/tubulin.tif]

#1

P-JNK

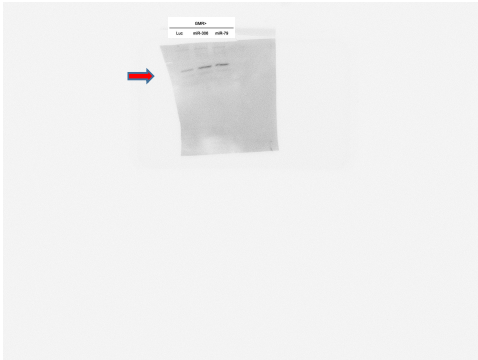

JNK

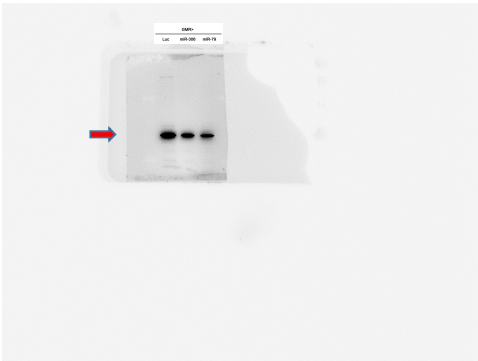

tubulin

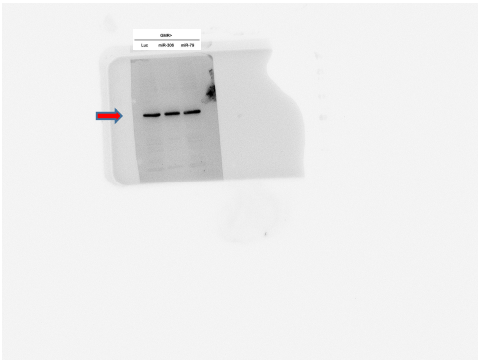

#2&#3

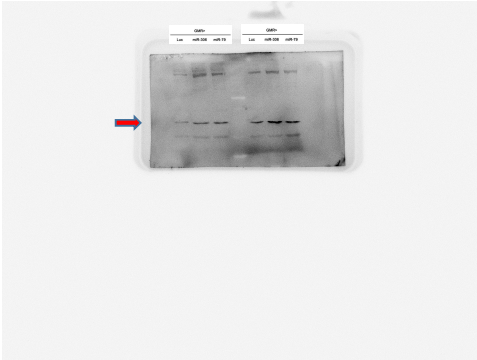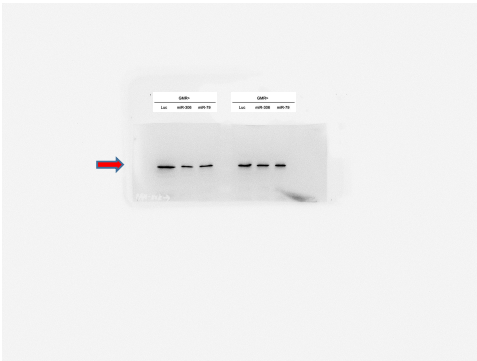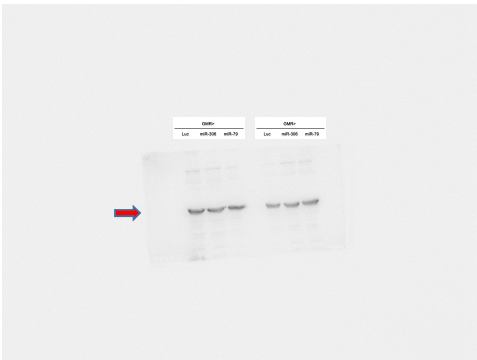

Supplement: Figure 3—figure supplement 2—source data 1. [file elife-77340-fig3-figsupp2-data1.zip › Figure 3—figure supplement 2—source data 1/Figure 3—figure supplement 2G uncropped blots with label/Figure 3—figure supplement 2G with label.pdf]

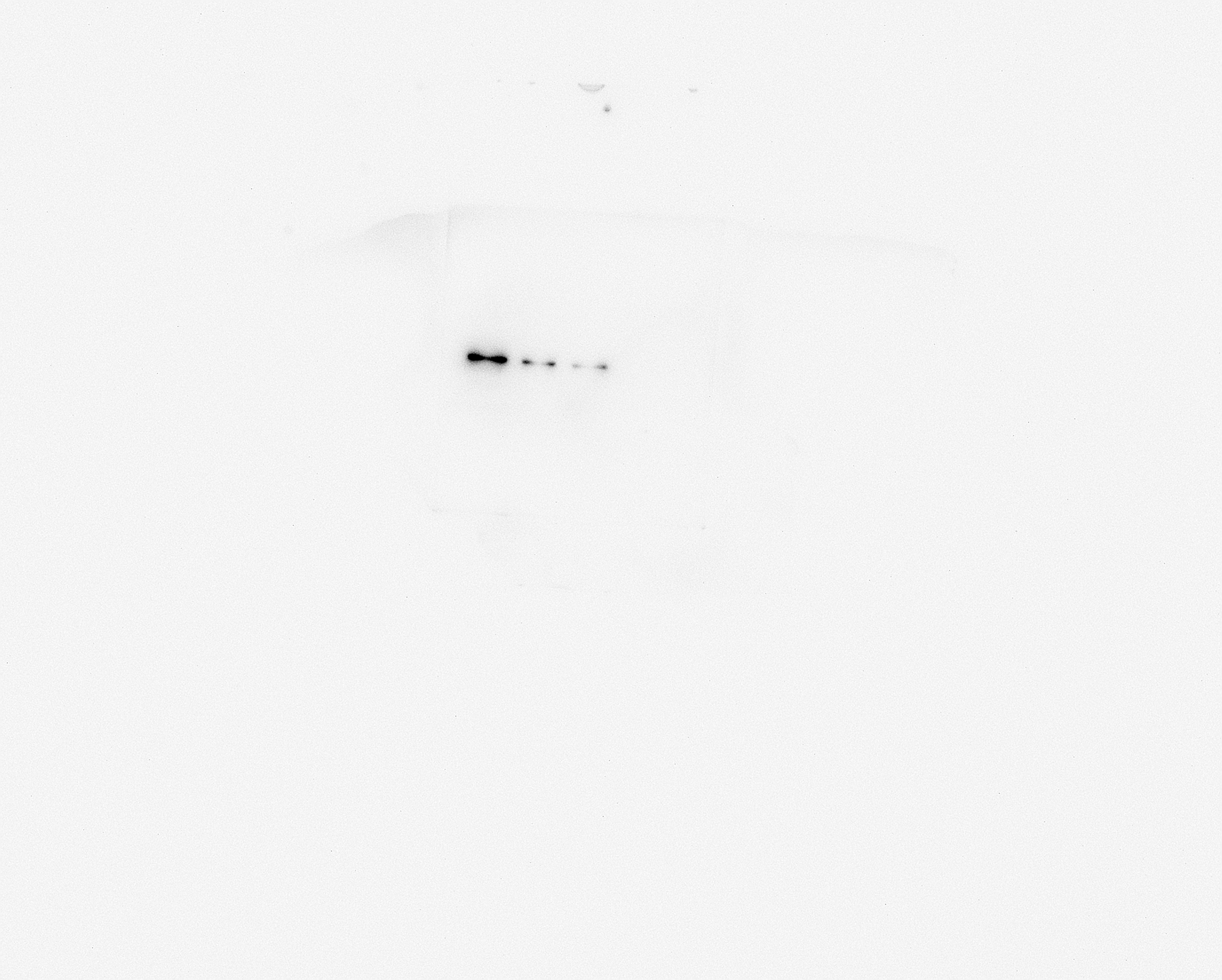

Supplement: Figure 5—source data 1. [file elife-77340-fig5-data1.zip › Figure 5—source data 1/Figure 5F raw data/#1/RNF146/RNF146.tif]

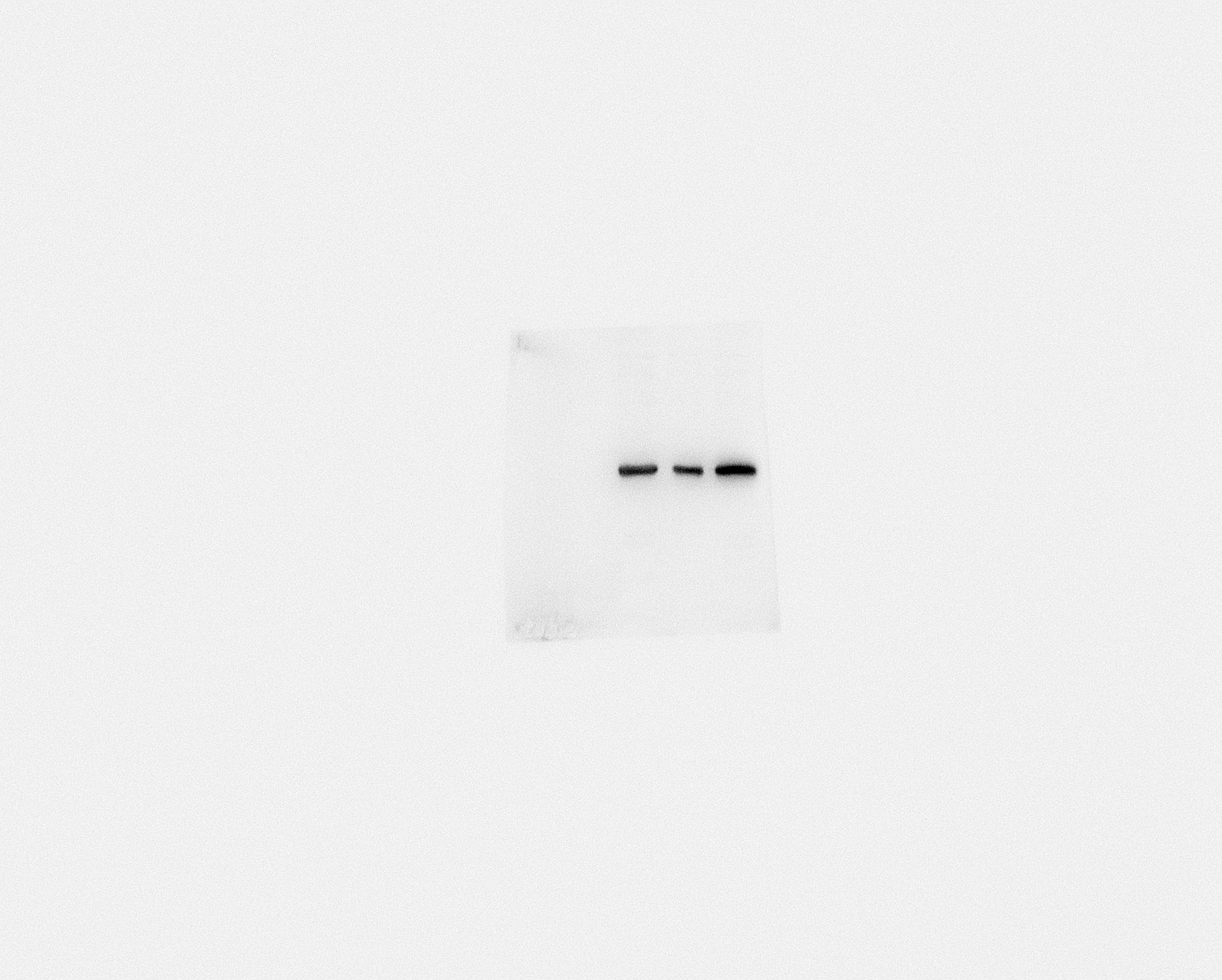

Supplement: Figure 5—source data 1. [file elife-77340-fig5-data1.zip › Figure 5—source data 1/Figure 5F raw data/#1/tubulin/tubulin.tif]

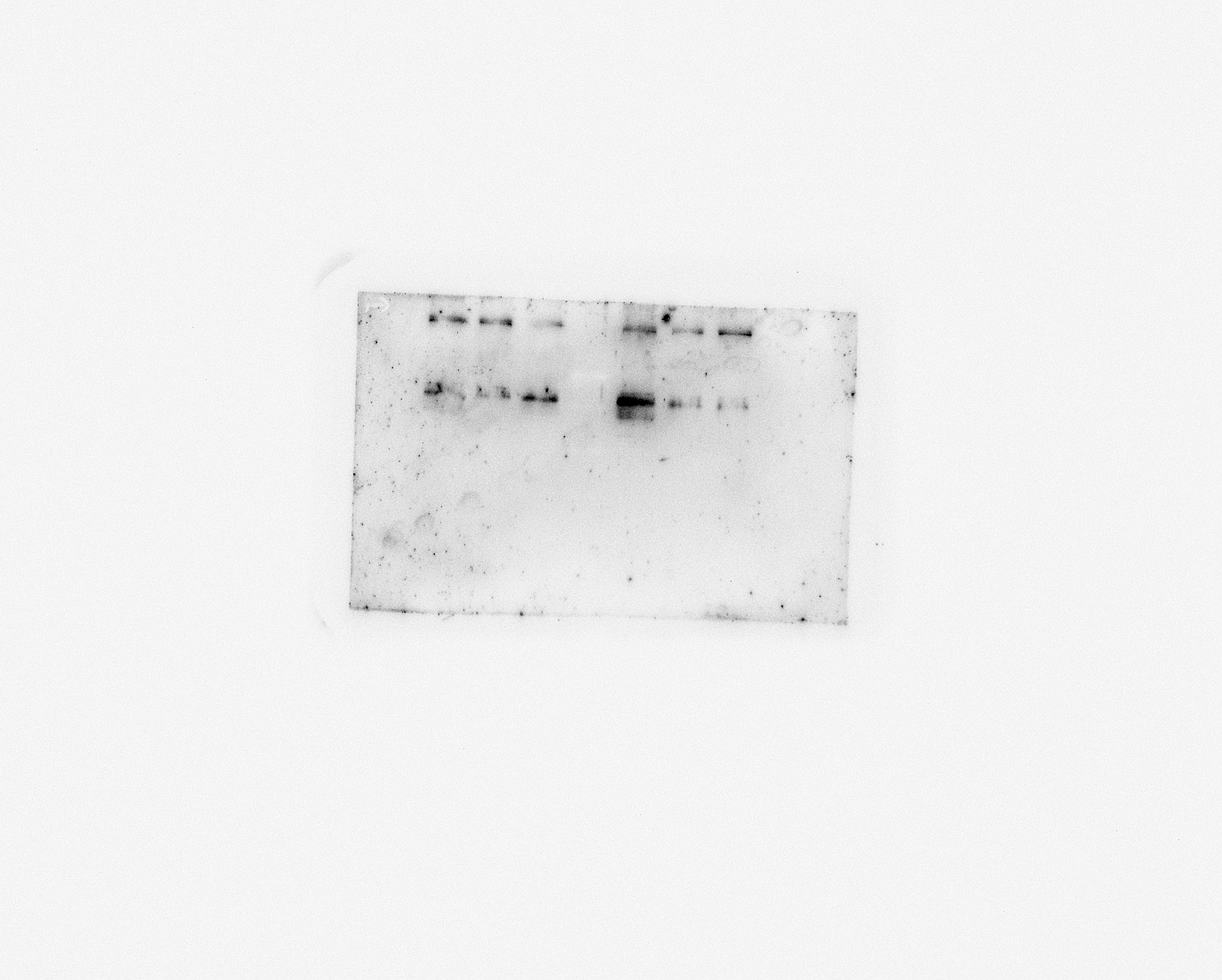

Supplement: Figure 5—source data 1. [file elife-77340-fig5-data1.zip › Figure 5—source data 1/Figure 5F raw data/#2#3/RNF146/RNF146.tif]

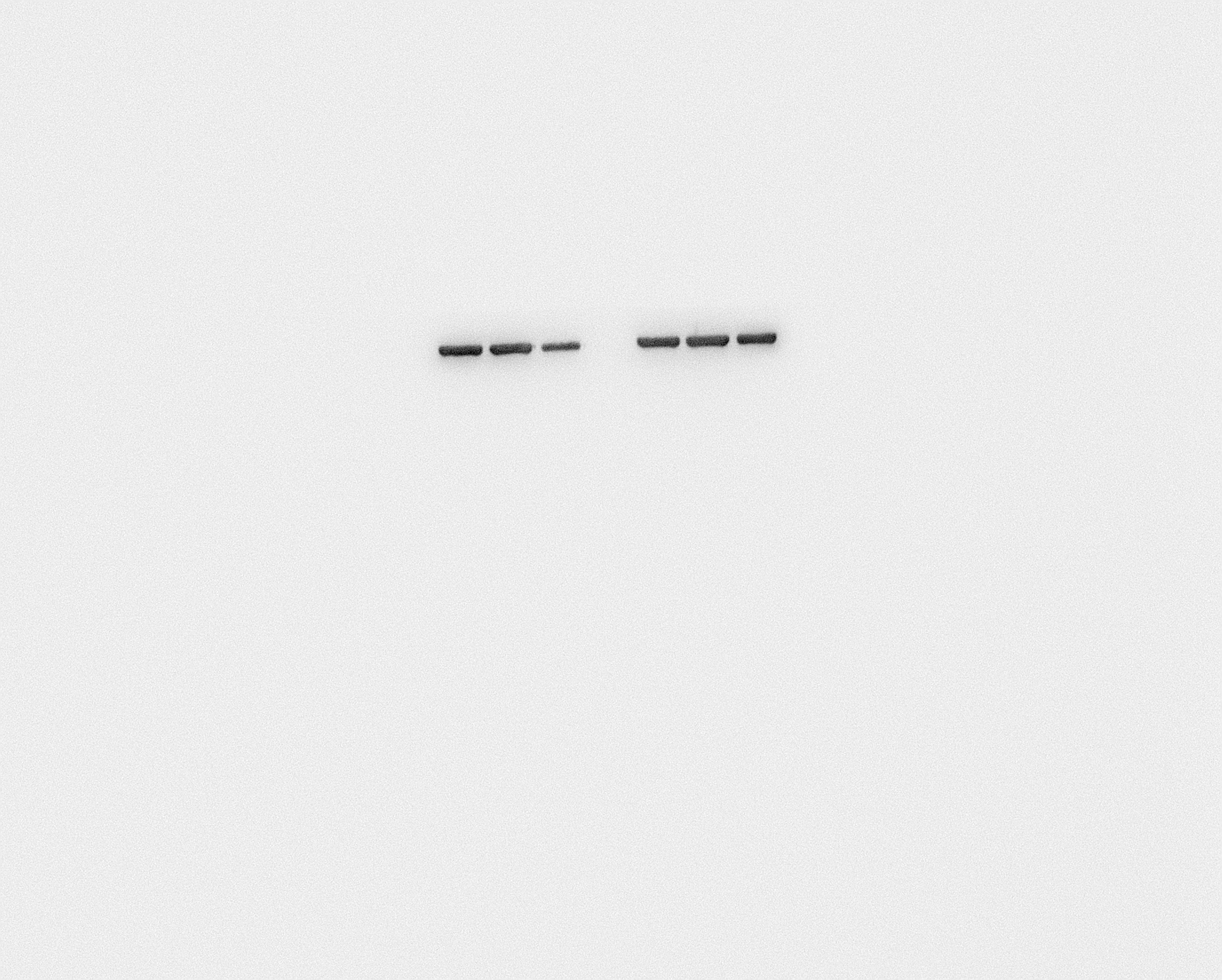

Supplement: Figure 5—source data 1. [file elife-77340-fig5-data1.zip › Figure 5—source data 1/Figure 5F raw data/#2#3/tubulin/tubulin.tif]

#1

RNF146

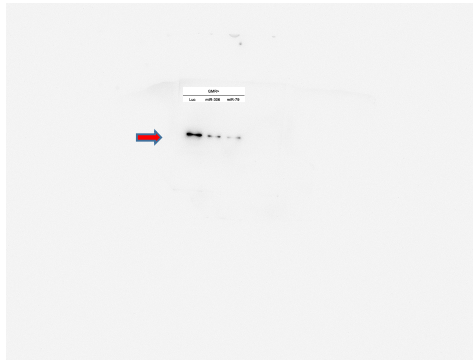

Tubulin

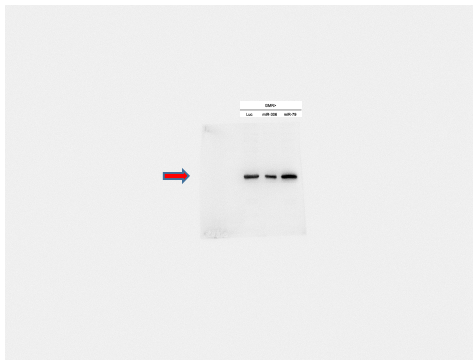

#2&#3

RNF146

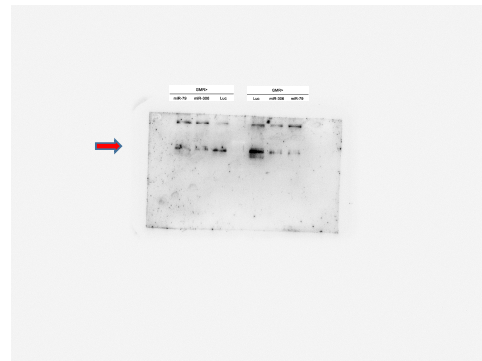

Tubulin

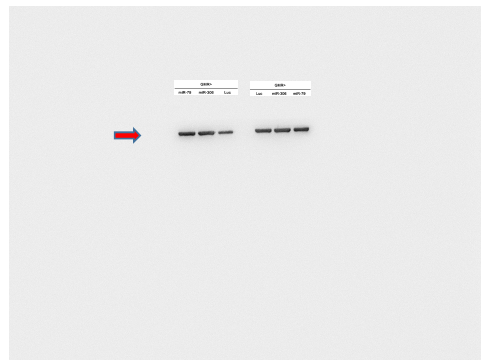

Supplement: Figure 5—source data 1. [file elife-77340-fig5-data1.zip › Figure 5—source data 1/Figure 5F uncropped blots with label/Figure 5F with label.pdf]

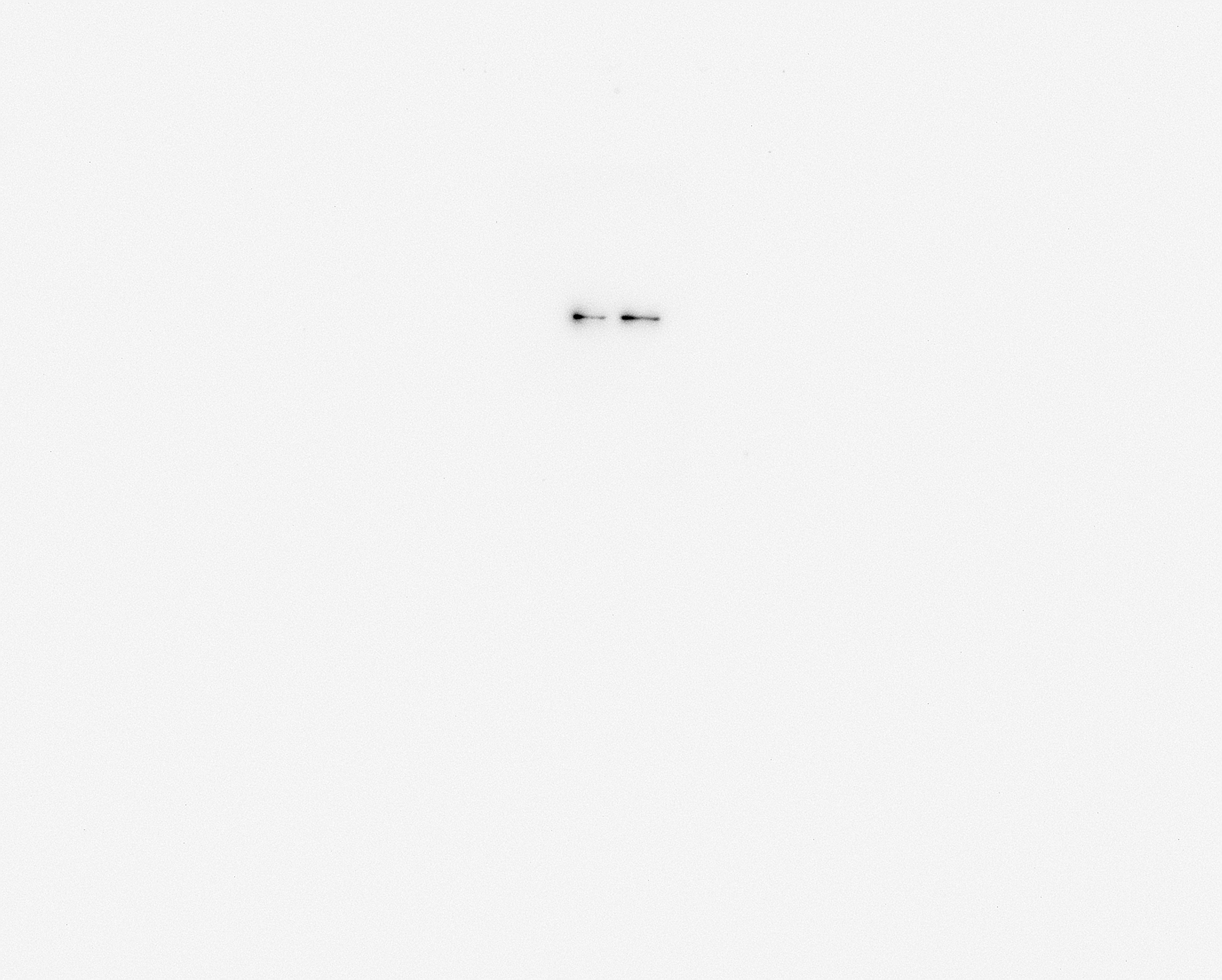

Supplement: Figure 5—figure supplement 3—source data 1. [file elife-77340-fig5-figsupp3-data1.zip › Figure 5—figure supplement 3—source data 1/Figure 5—figure supplement 3A raw data/#1/RNF146/RNF146.tif]

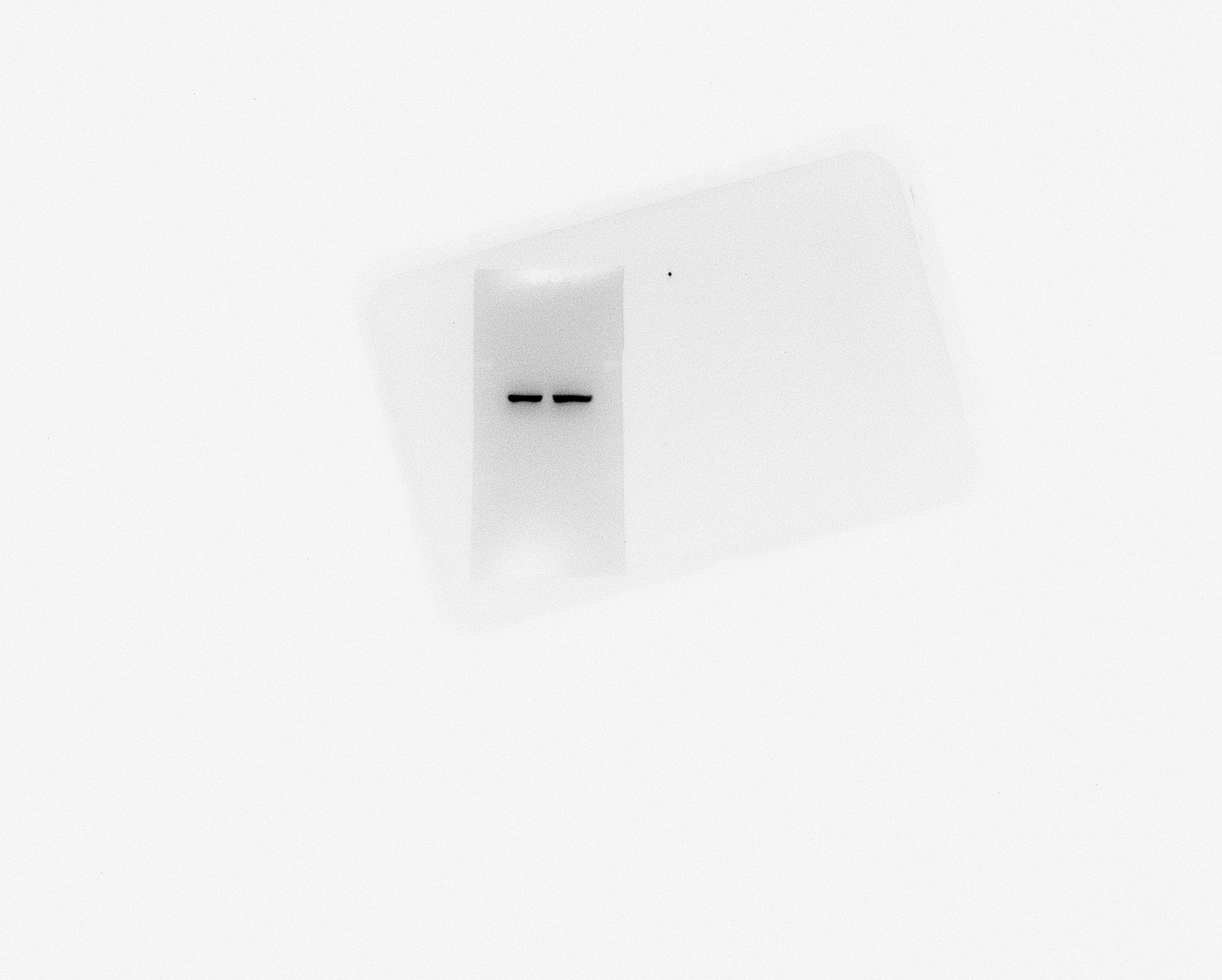

Supplement: Figure 5—figure supplement 3—source data 1. [file elife-77340-fig5-figsupp3-data1.zip › Figure 5—figure supplement 3—source data 1/Figure 5—figure supplement 3A raw data/#1/tubulin/tubulin.tif]

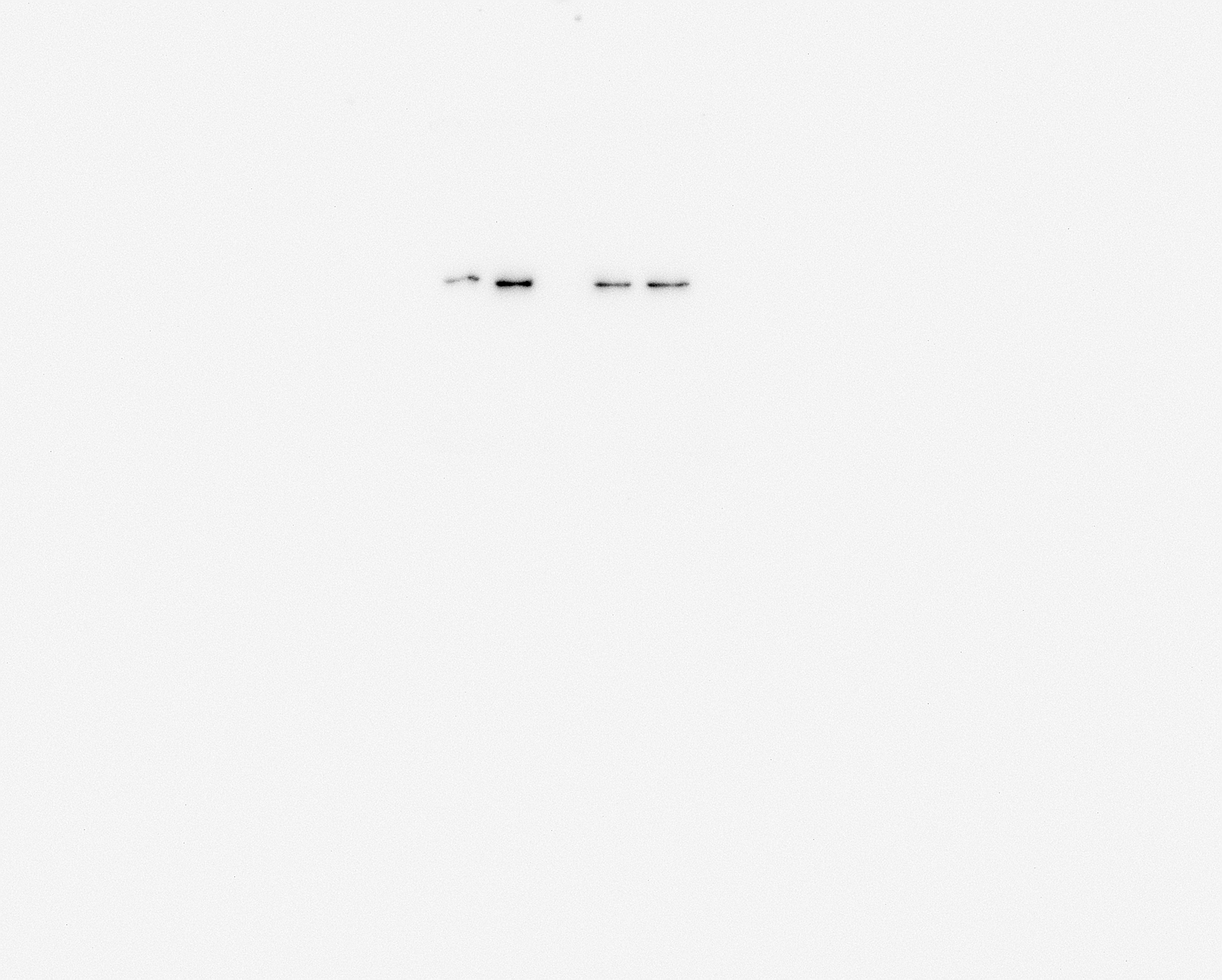

Supplement: Figure 5—figure supplement 3—source data 1. [file elife-77340-fig5-figsupp3-data1.zip › Figure 5—figure supplement 3—source data 1/Figure 5—figure supplement 3A raw data/#2#3/RNF146/RNF146.tif]

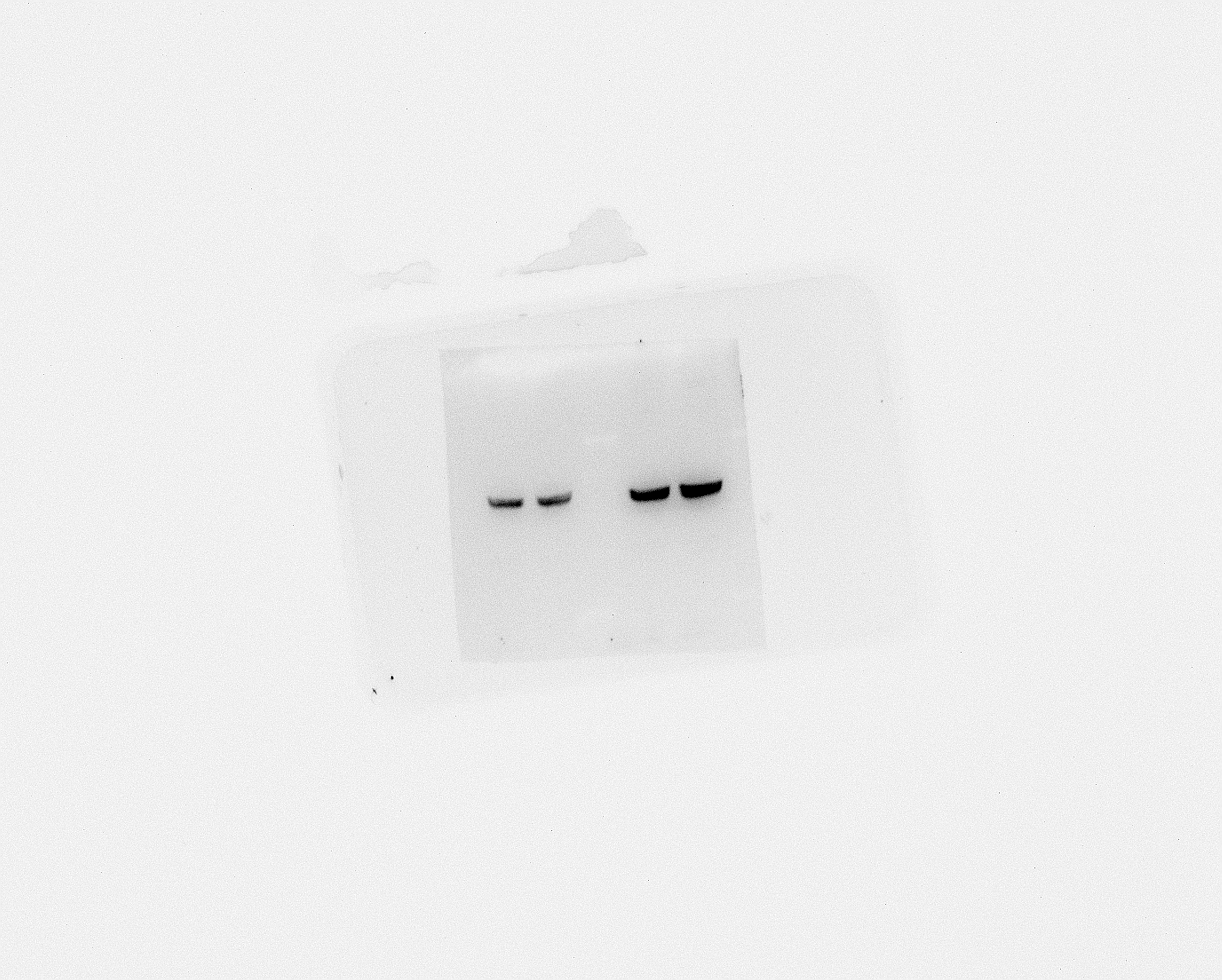

Supplement: Figure 5—figure supplement 3—source data 1. [file elife-77340-fig5-figsupp3-data1.zip › Figure 5—figure supplement 3—source data 1/Figure 5—figure supplement 3A raw data/#2#3/tubulin/tubulin.tif]

#1

RNF146

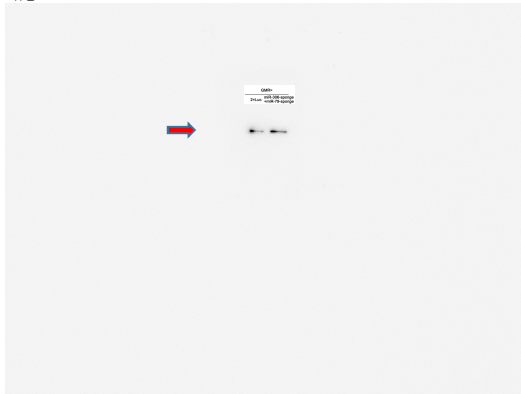

#2&#3

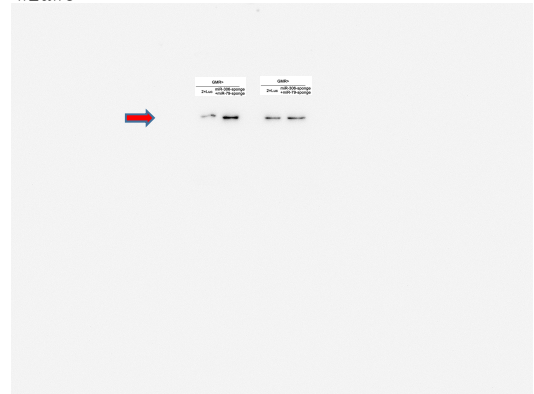

tubulin

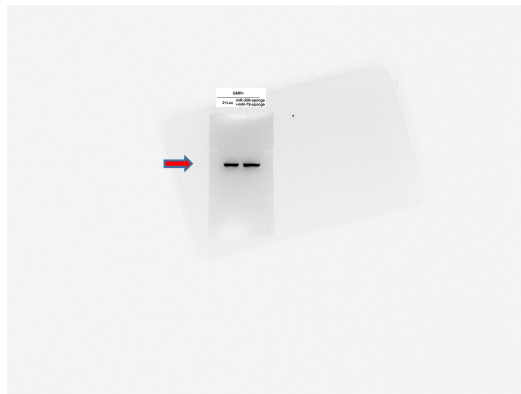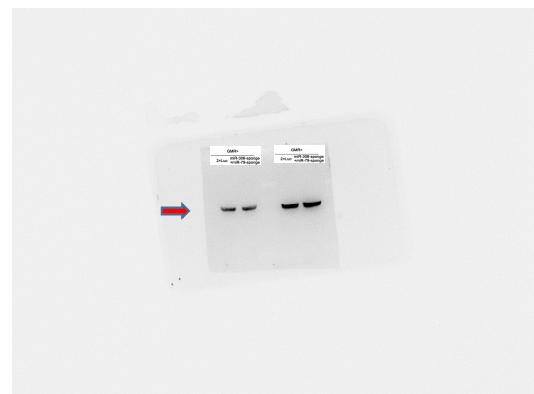

Supplement: Figure 5—figure supplement 3—source data 1. [file elife-77340-fig5-figsupp3-data1.zip › Figure 5—figure supplement 3—source data 1/Figure 5—figure supplement 3A uncropped blots with label/Figure 5—figure supplement 3A with label.pdf]

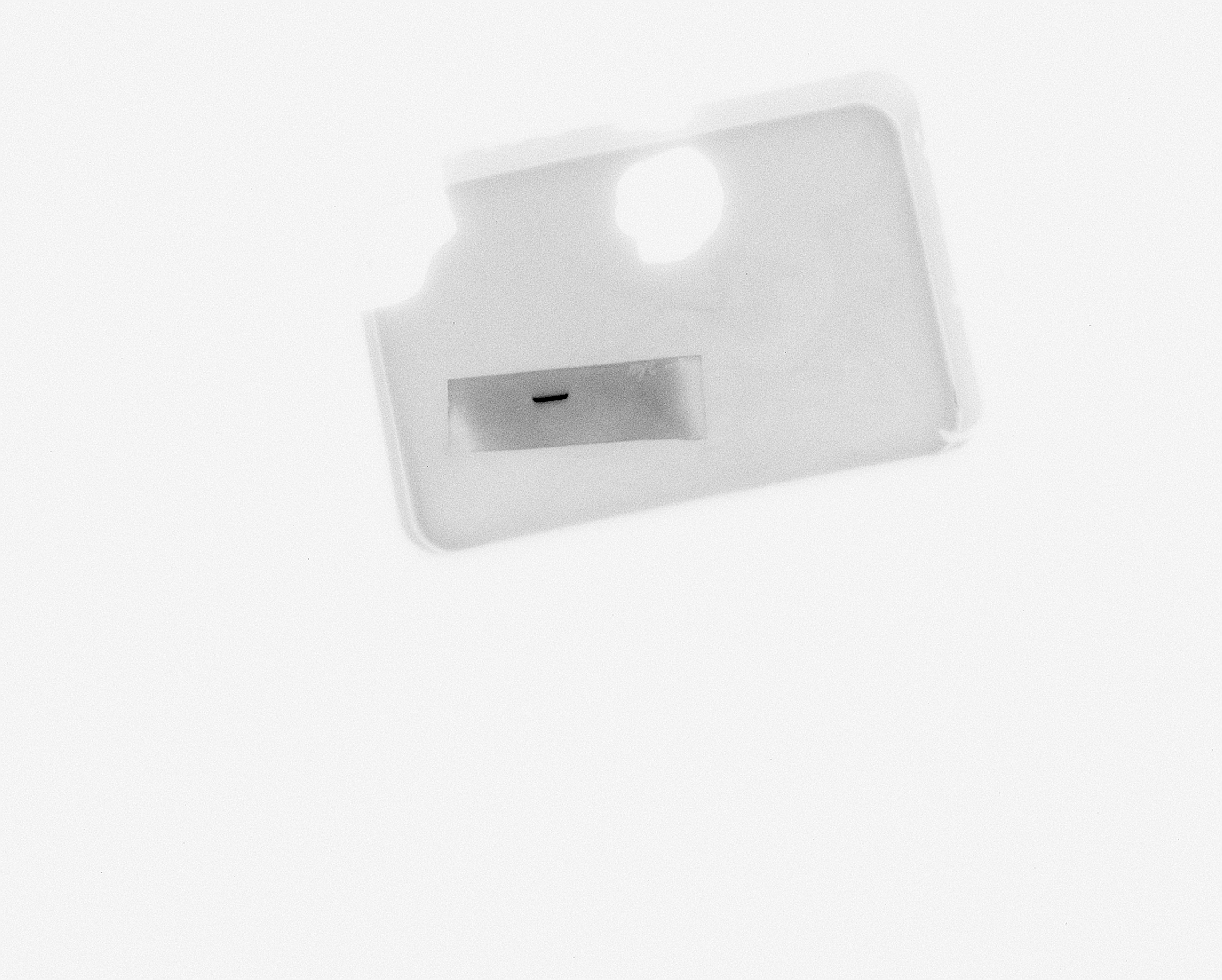

Supplement: Figure 6—source data 1. [file elife-77340-fig6-data1.zip › Figure 6A raw data/#1/myc/myc.tif]

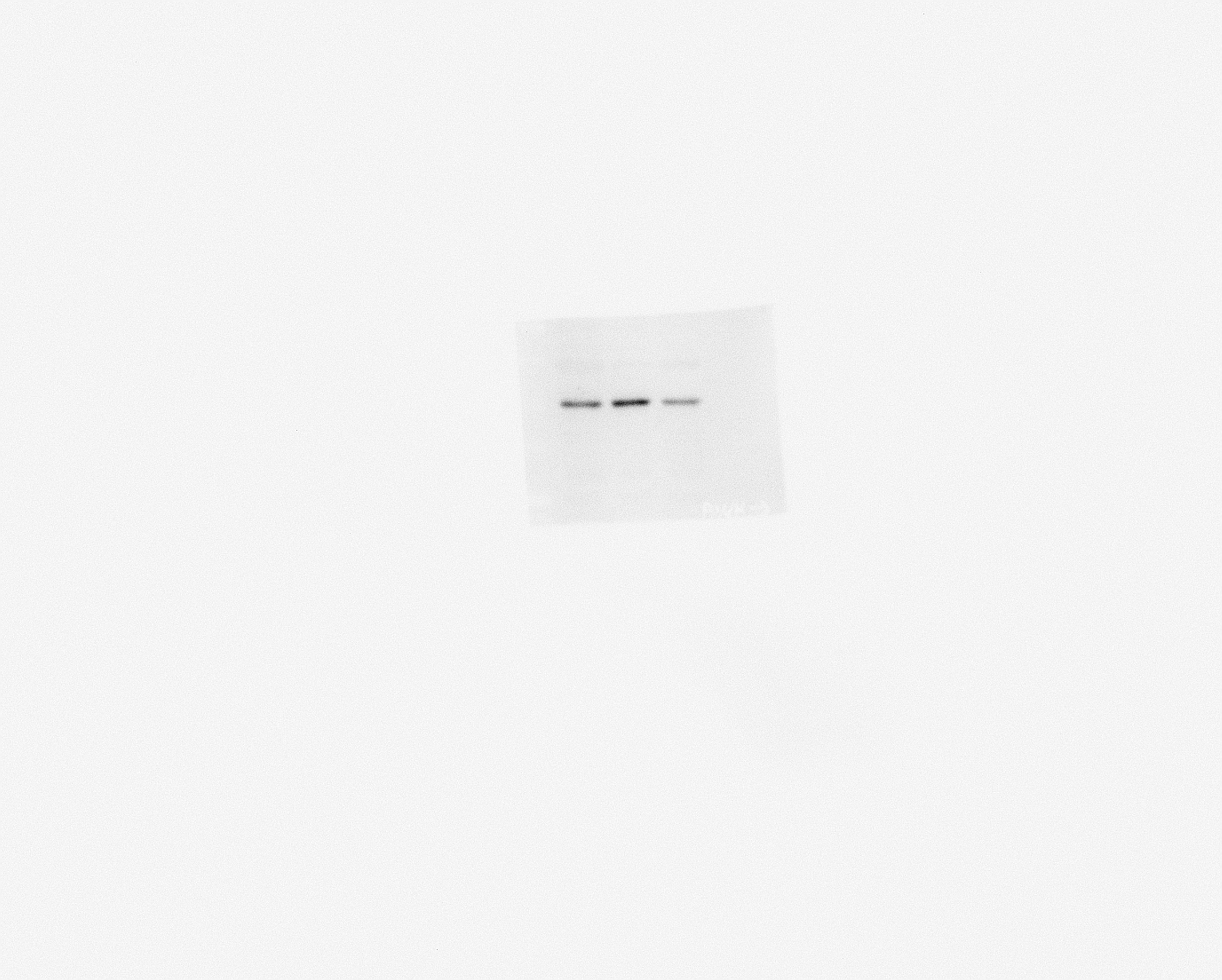

Supplement: Figure 6—source data 1. [file elife-77340-fig6-data1.zip › Figure 6A raw data/#1/P-JNK/P-JNK.tif]

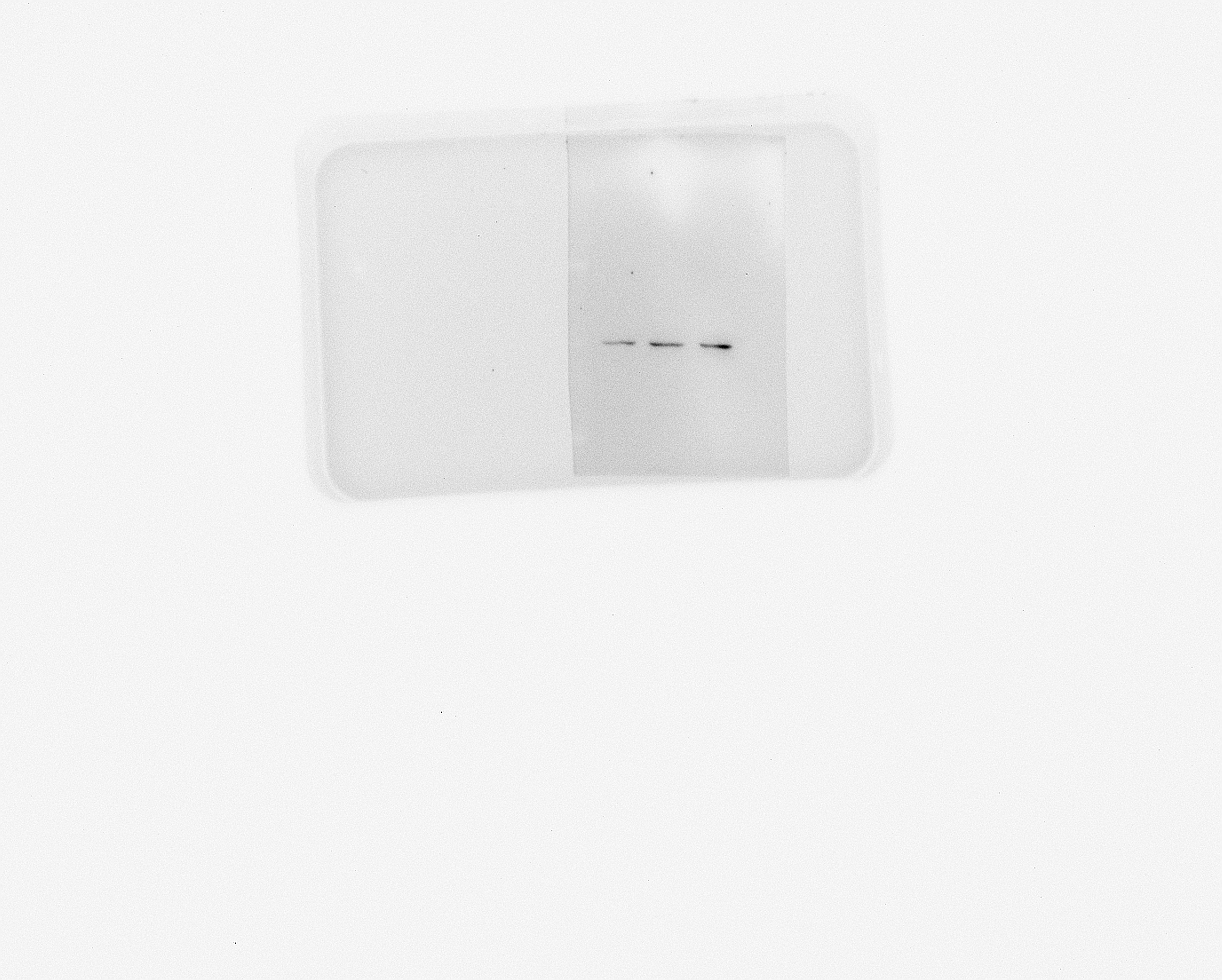

Supplement: Figure 6—source data 1. [file elife-77340-fig6-data1.zip › Figure 6A raw data/#1/total JNK/total JNK.tif]

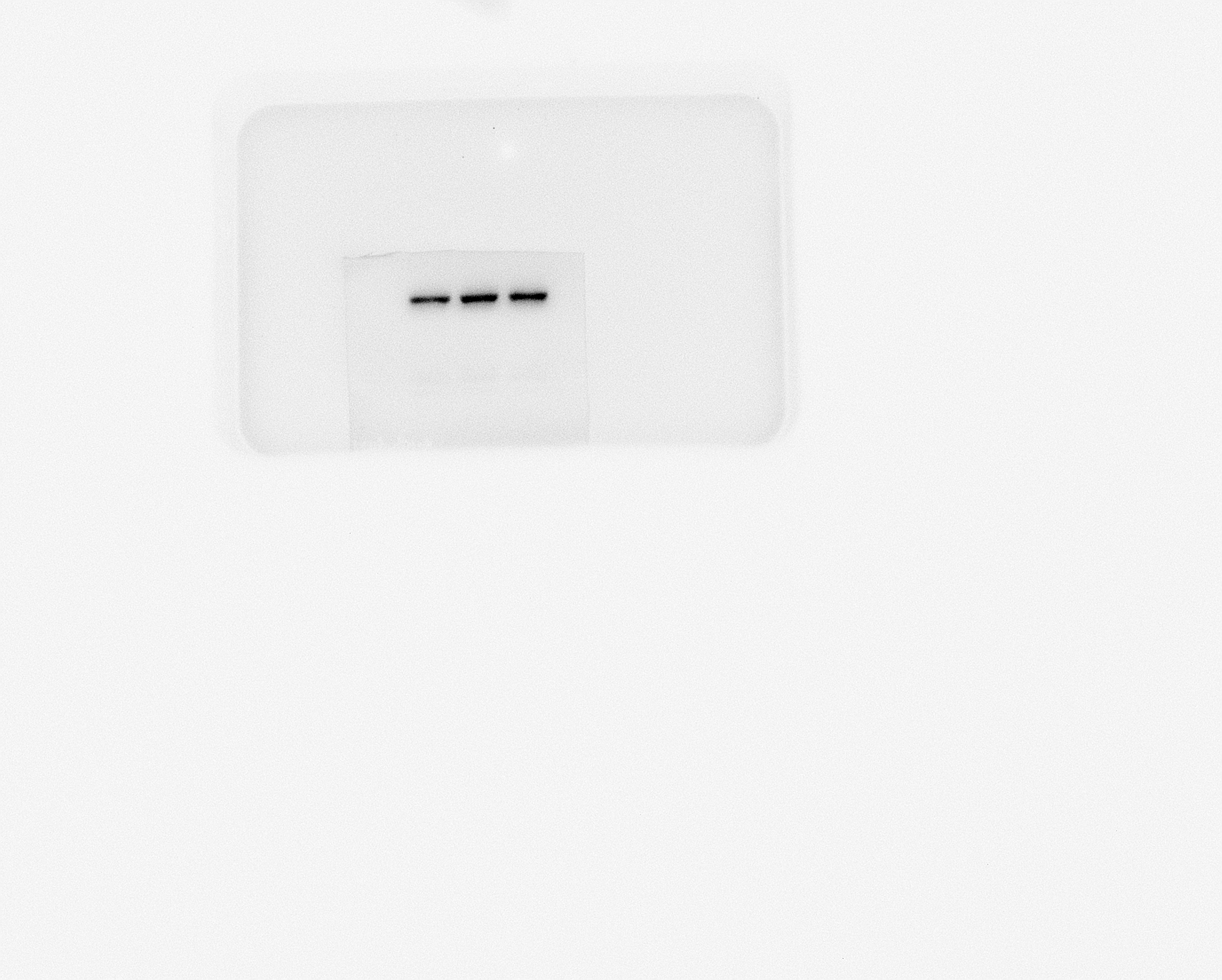

Supplement: Figure 6—source data 1. [file elife-77340-fig6-data1.zip › Figure 6A raw data/#1/tubulin/tubulin.tif]

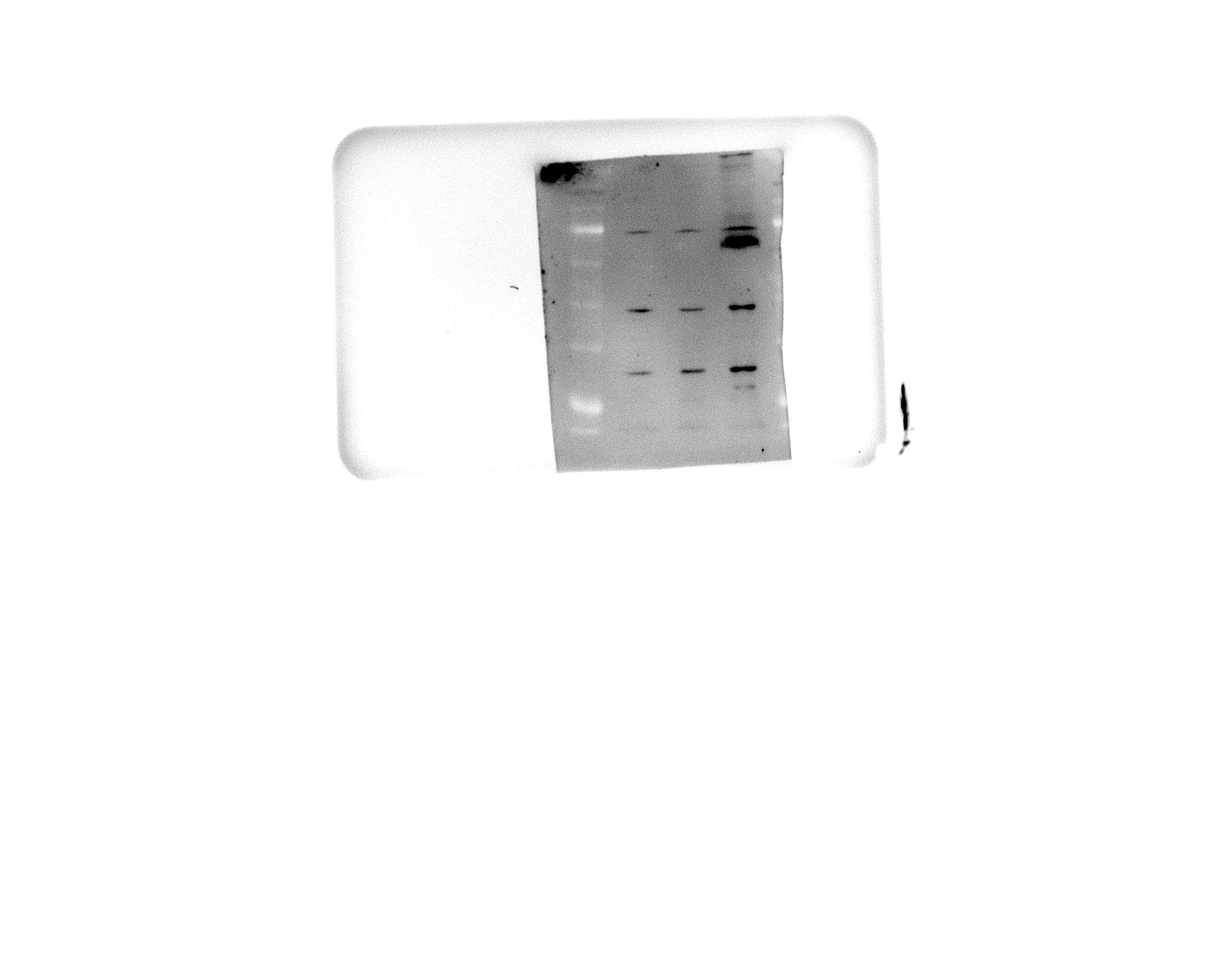

Supplement: Figure 6—source data 1. [file elife-77340-fig6-data1.zip › Figure 6A raw data/#1/V5/V5.tif]

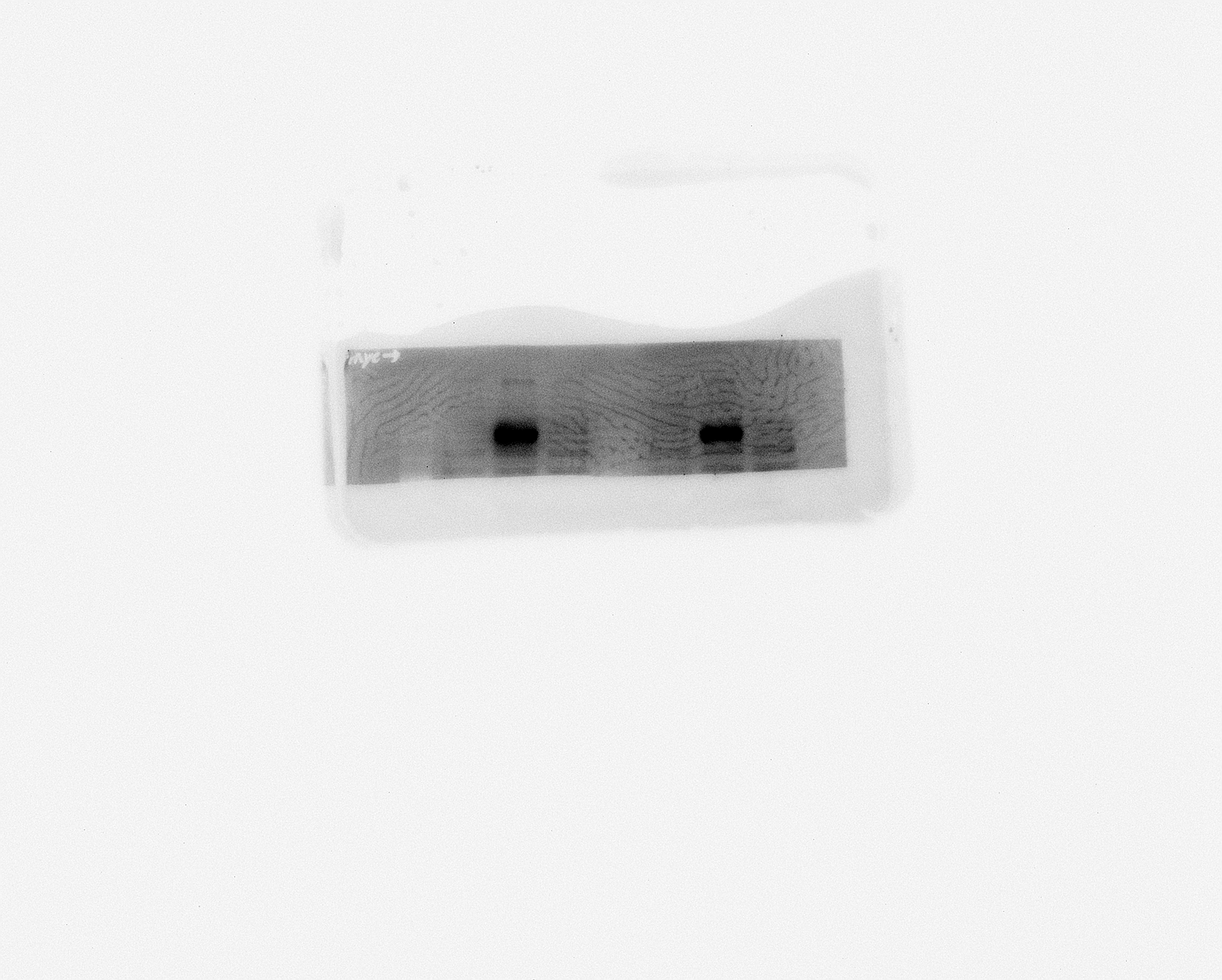

Supplement: Figure 6—source data 1. [file elife-77340-fig6-data1.zip › Figure 6A raw data/#2#3/myc/myc.tif]

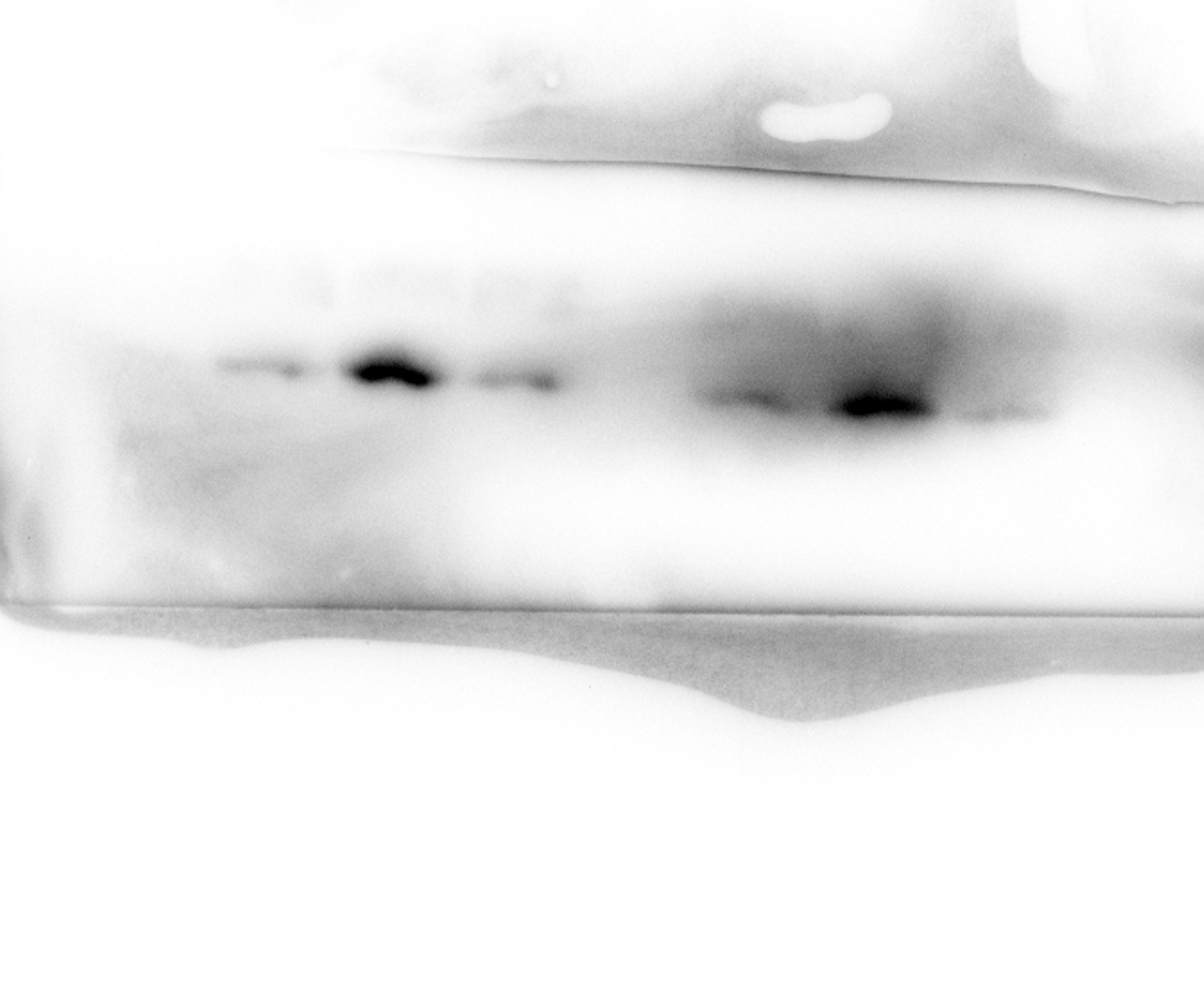

Supplement: Figure 6—source data 1. [file elife-77340-fig6-data1.zip › Figure 6A raw data/#2#3/P-JNK/P-JNK.Tif]

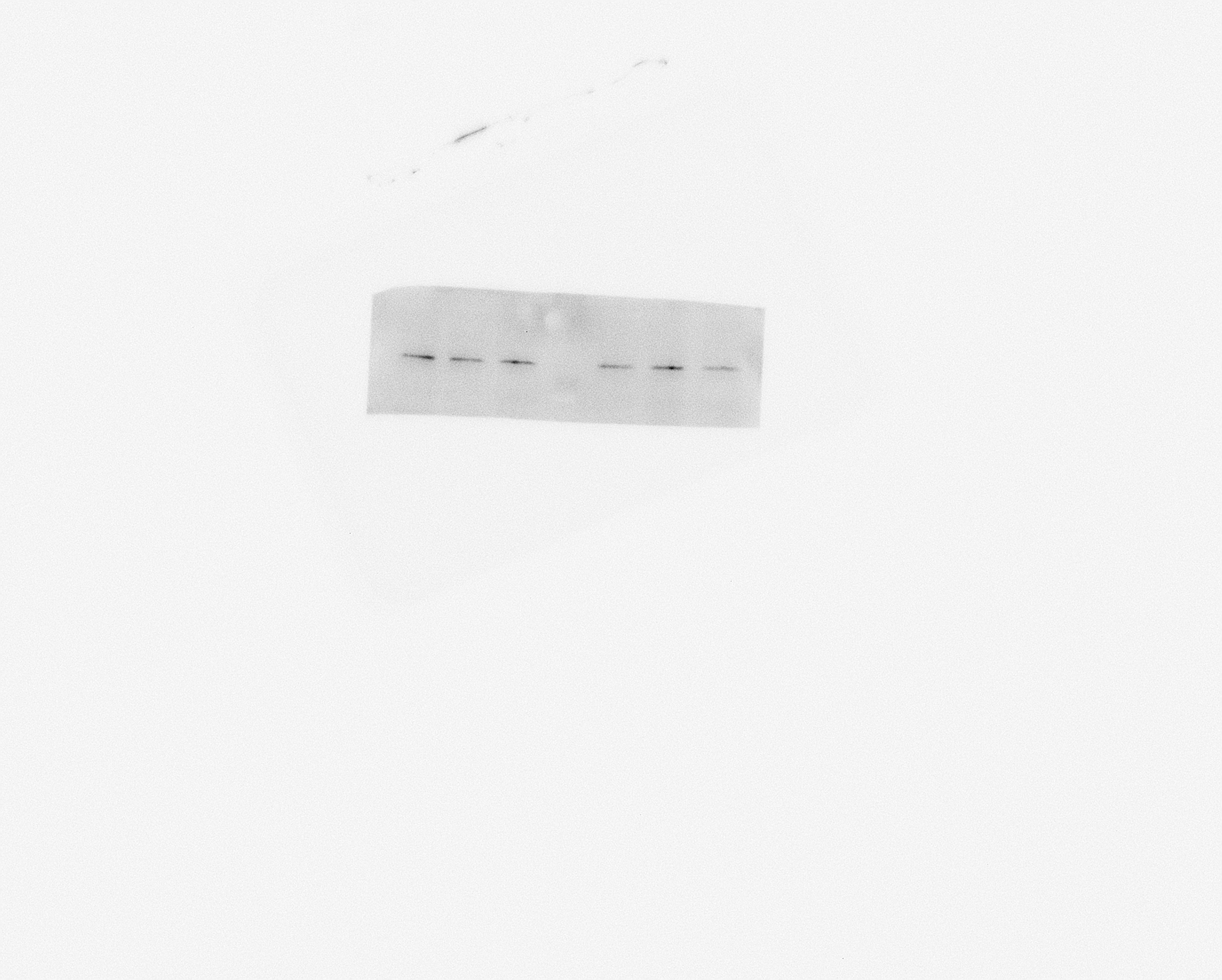

Supplement: Figure 6—source data 1. [file elife-77340-fig6-data1.zip › Figure 6A raw data/#2#3/total JNK/total JNK.tif]

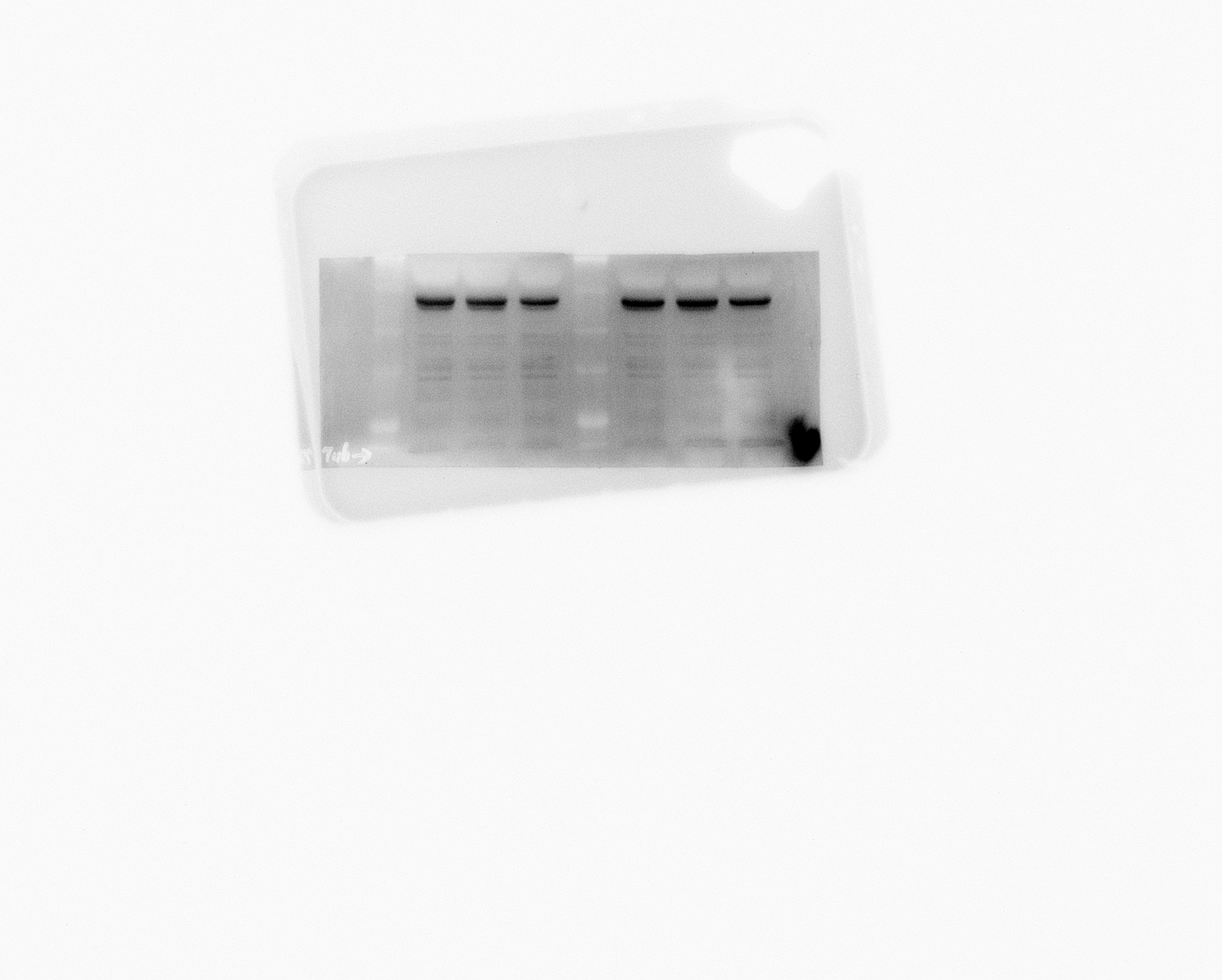

Supplement: Figure 6—source data 1. [file elife-77340-fig6-data1.zip › Figure 6A raw data/#2#3/tubulin/tubulin.tif]

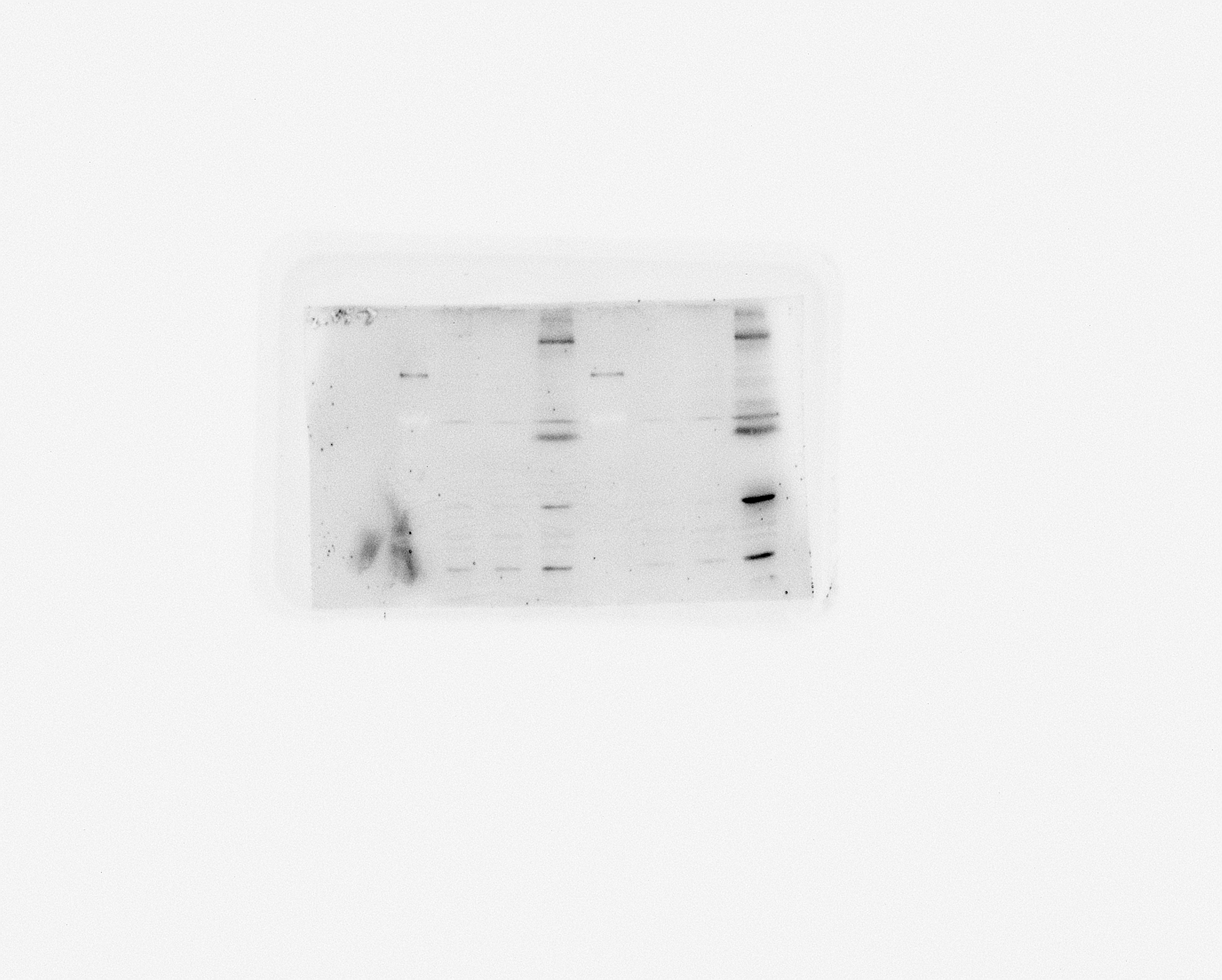

Supplement: Figure 6—source data 1. [file elife-77340-fig6-data1.zip › Figure 6A raw data/#2#3/V5/V5.tif]

#1

myc

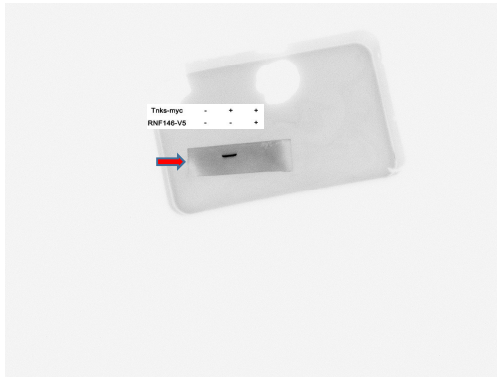

#2&#3

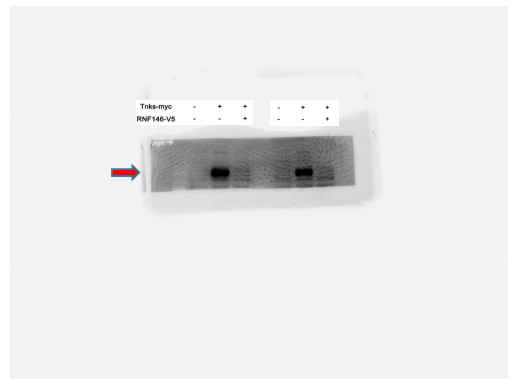

V5

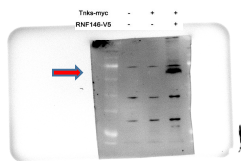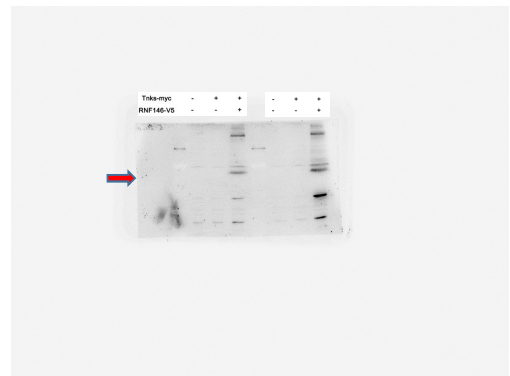

P-JNK

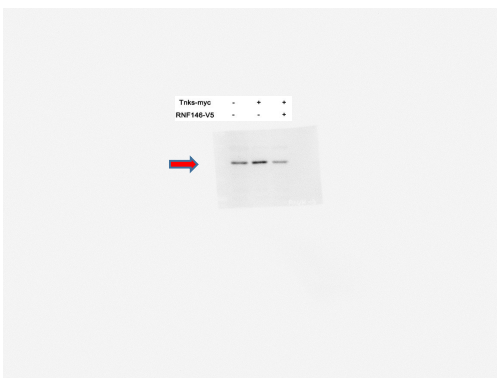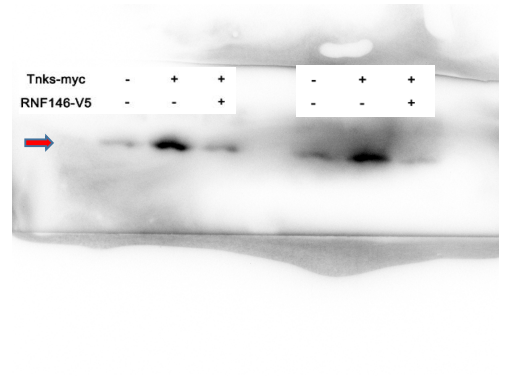

JNK

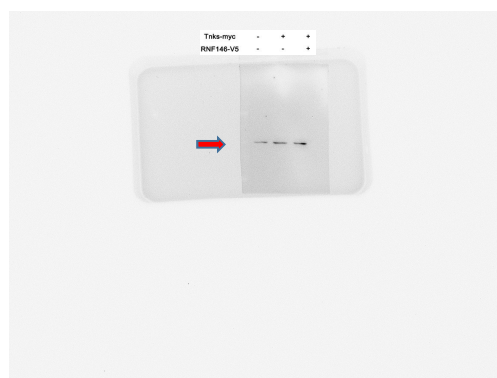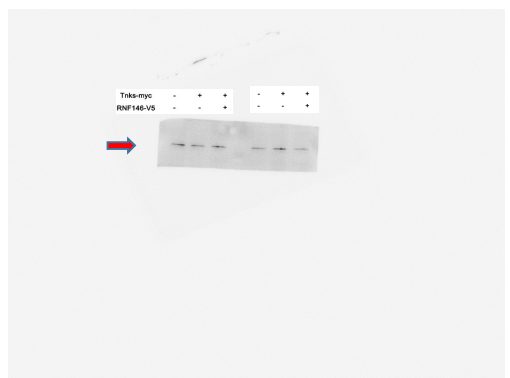

tubulin

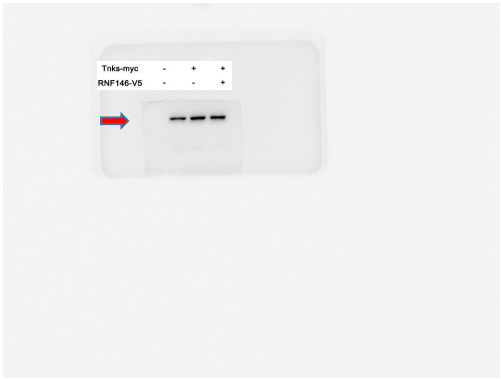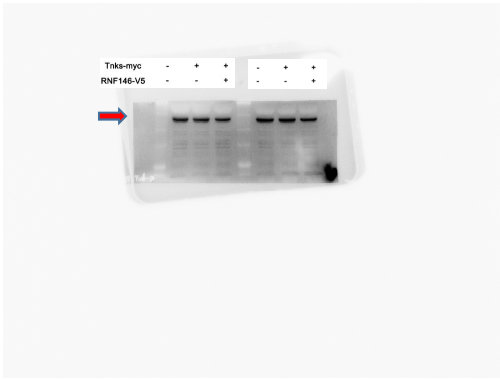

Supplement: Figure 6—source data 1. [file elife-77340-fig6-data1.zip › Figure 6A uncropped blots with label/Figure 6A with label.pdf]

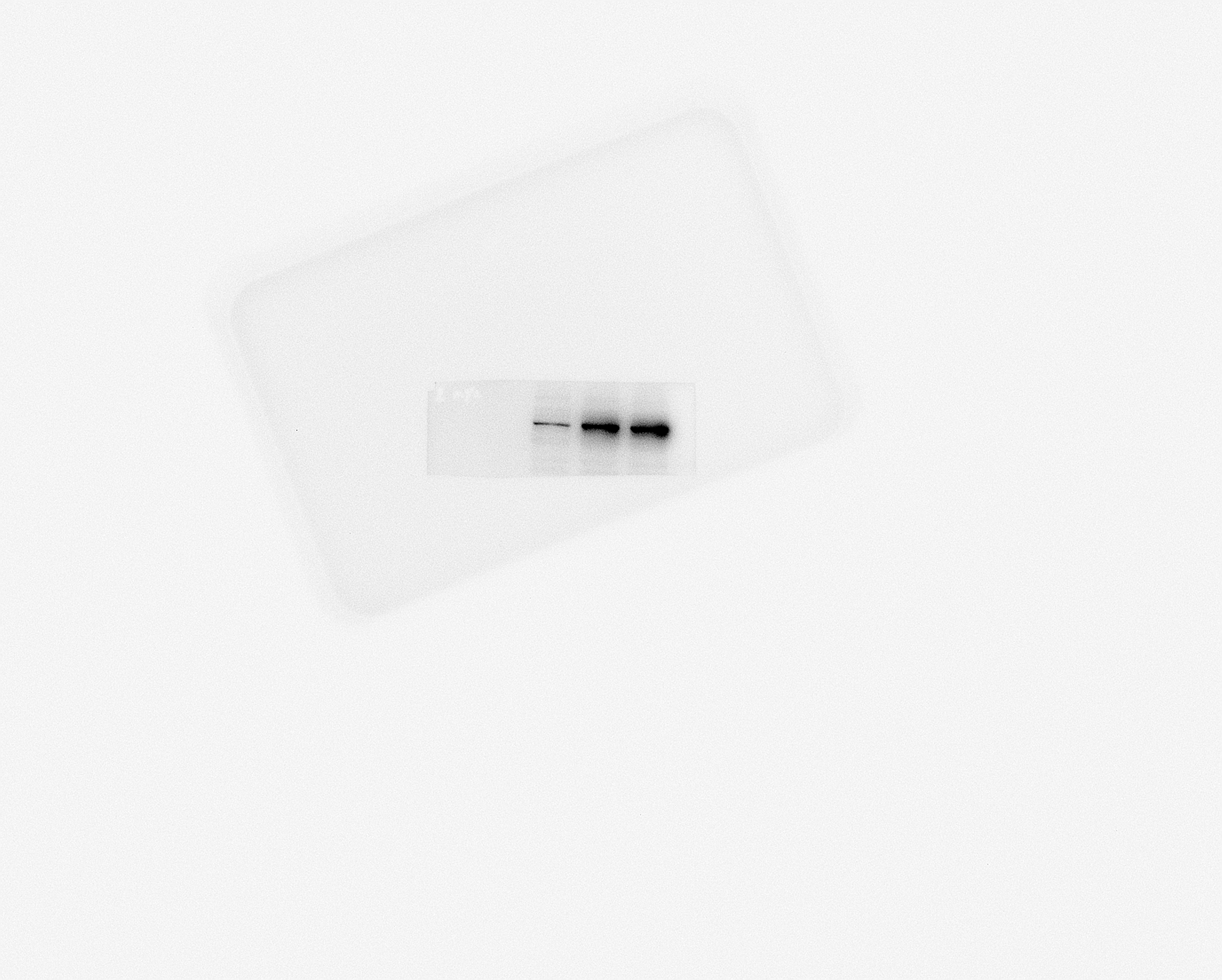

Supplement: Figure 6—source data 2. [file elife-77340-fig6-data2.zip › Figure 6D raw data/#1/myc/myc.tif]

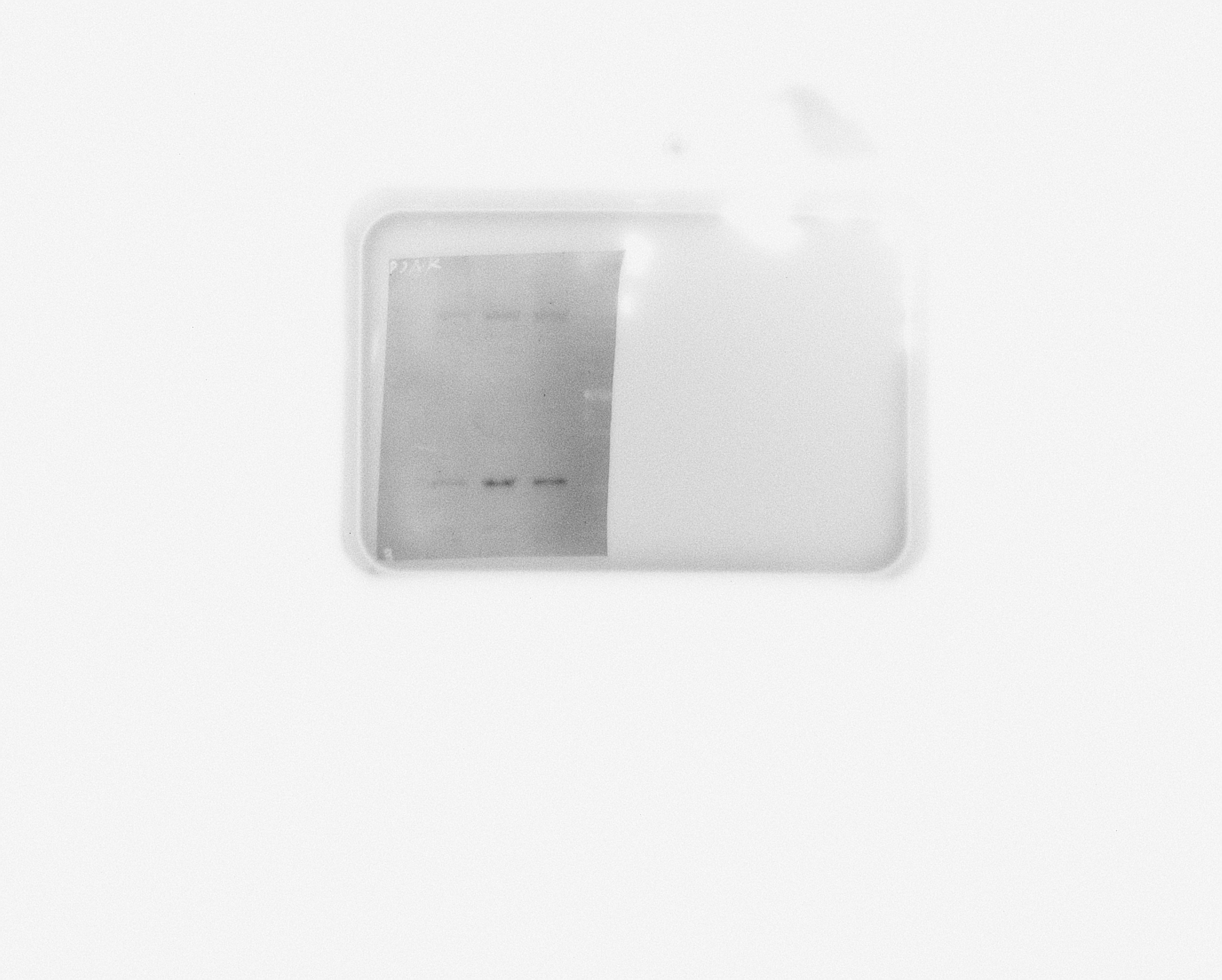

Supplement: Figure 6—source data 2. [file elife-77340-fig6-data2.zip › Figure 6D raw data/#1/P-JNK/P-JNK.tif]

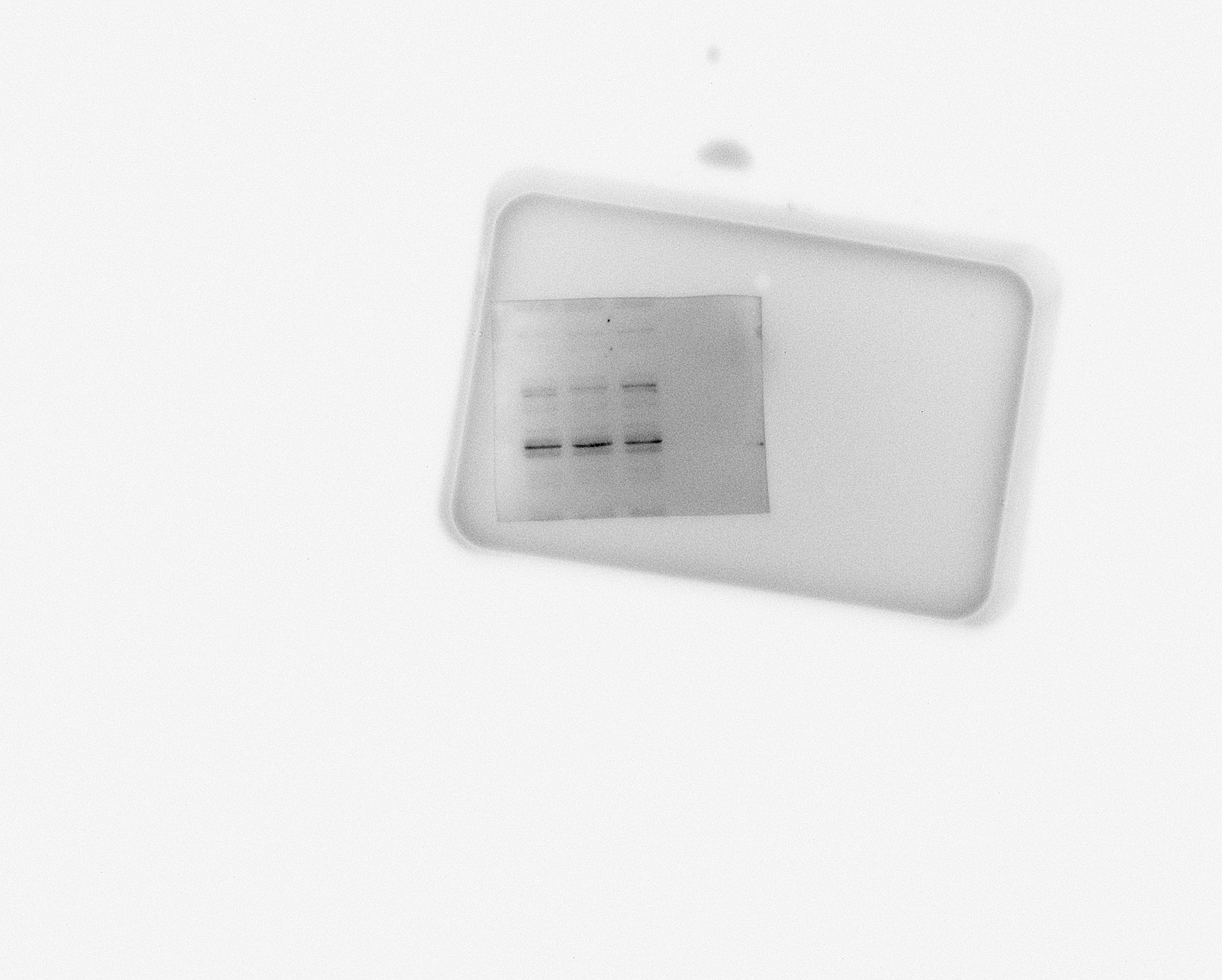

Supplement: Figure 6—source data 2. [file elife-77340-fig6-data2.zip › Figure 6D raw data/#1/total JNK/total JNK.tif]

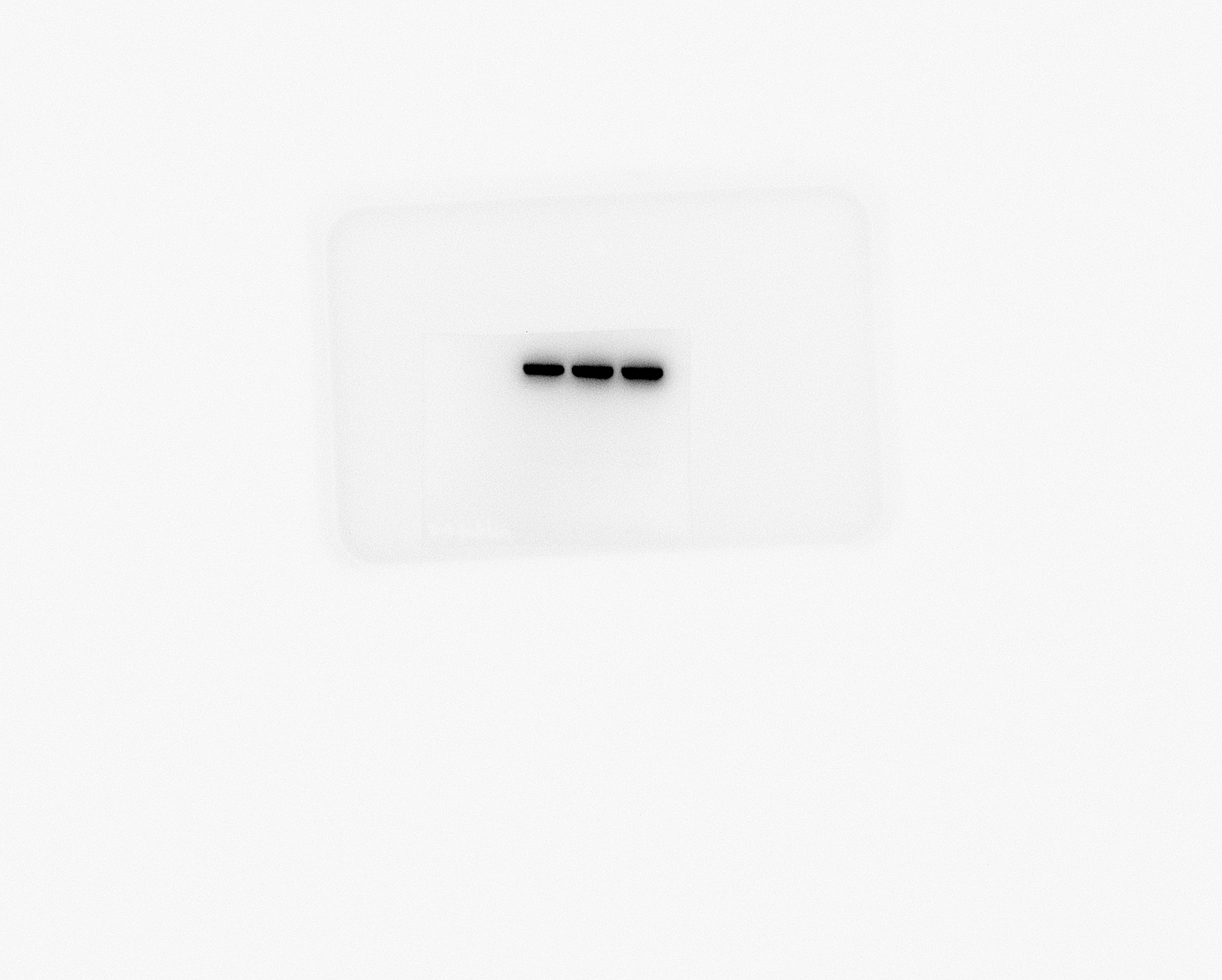

Supplement: Figure 6—source data 2. [file elife-77340-fig6-data2.zip › Figure 6D raw data/#1/tubulin/tubulin.tif]

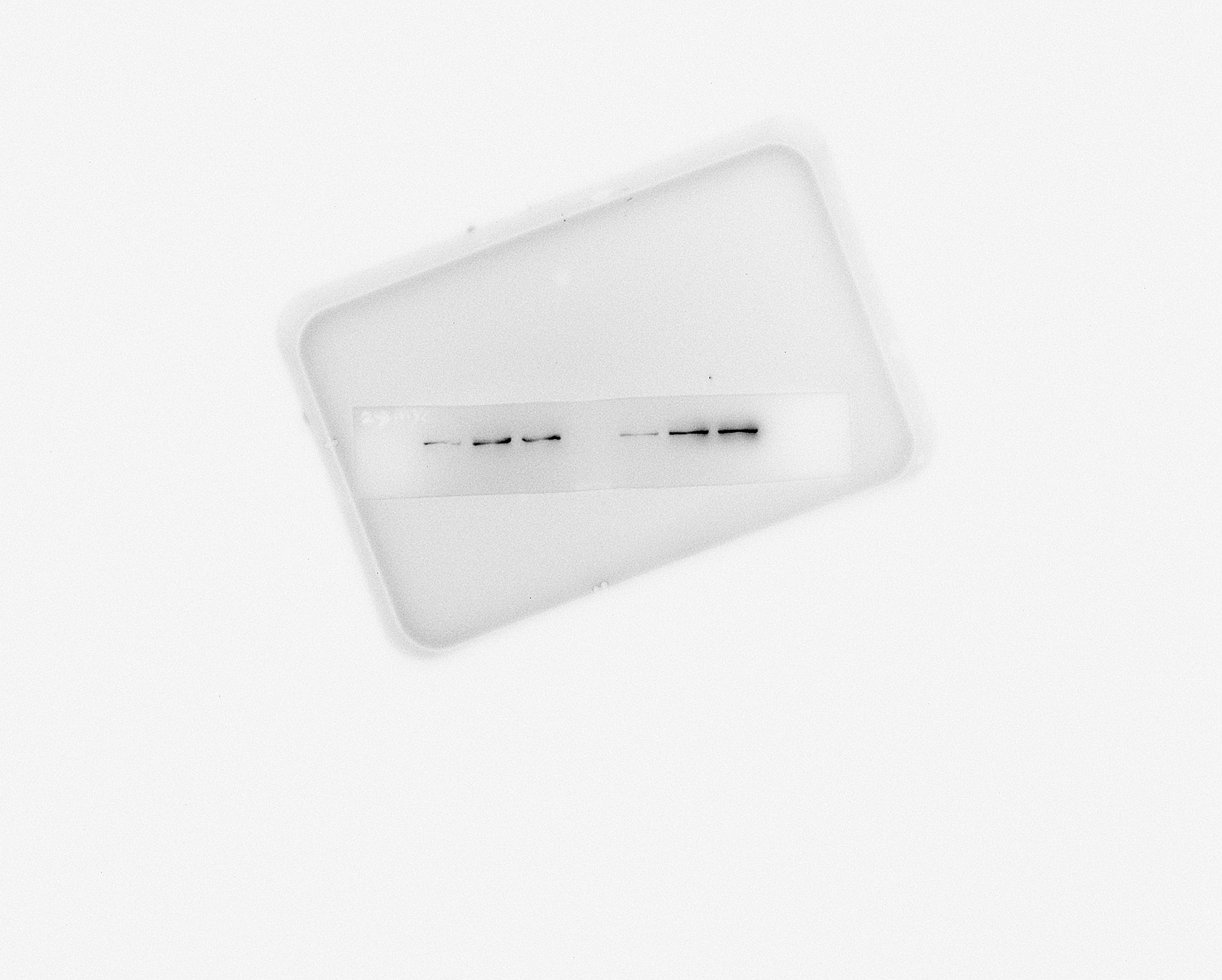

Supplement: Figure 6—source data 2. [file elife-77340-fig6-data2.zip › Figure 6D raw data/#2#3/myc/myc.tif]

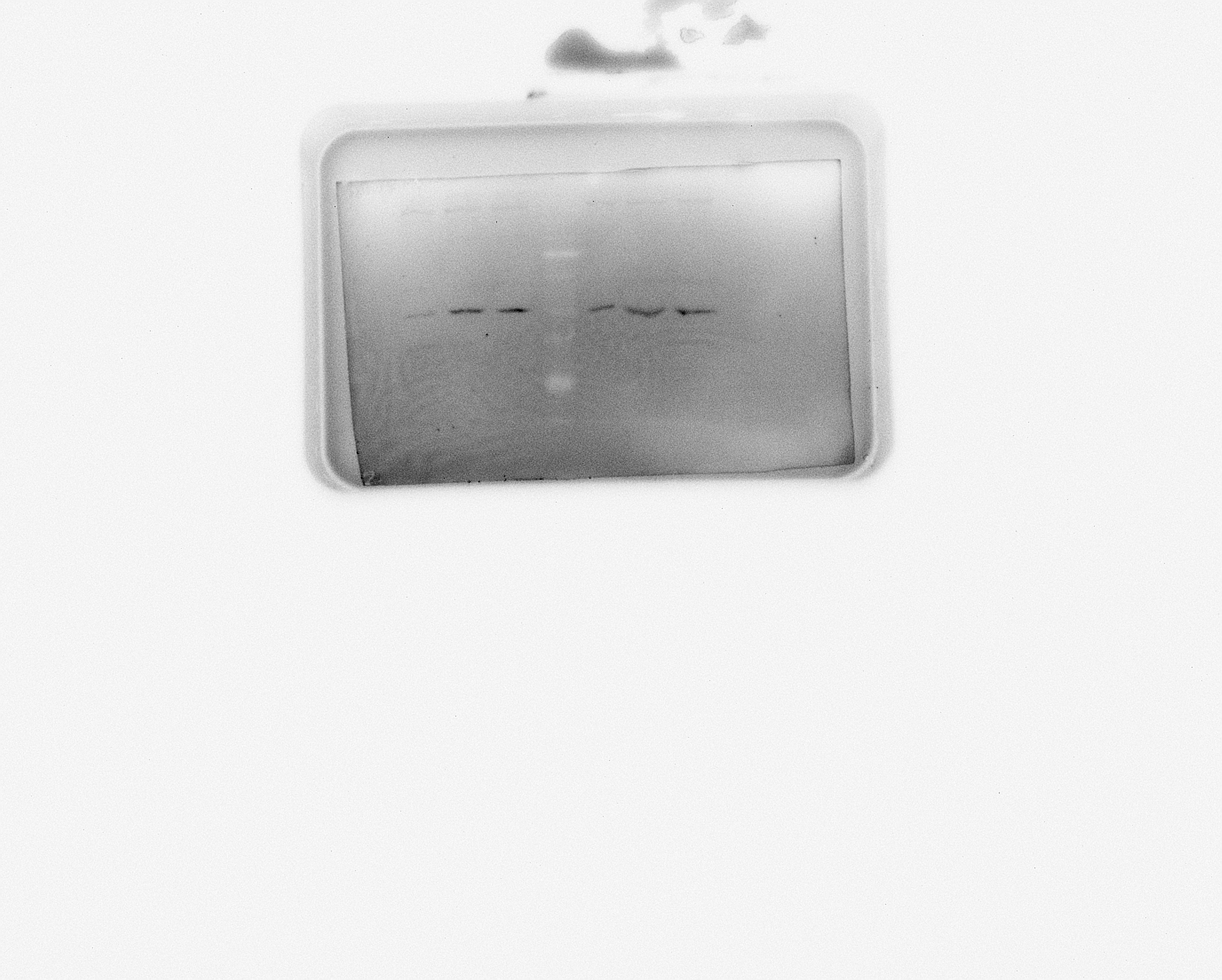

Supplement: Figure 6—source data 2. [file elife-77340-fig6-data2.zip › Figure 6D raw data/#2#3/P-JNK/P-JNK.tif]

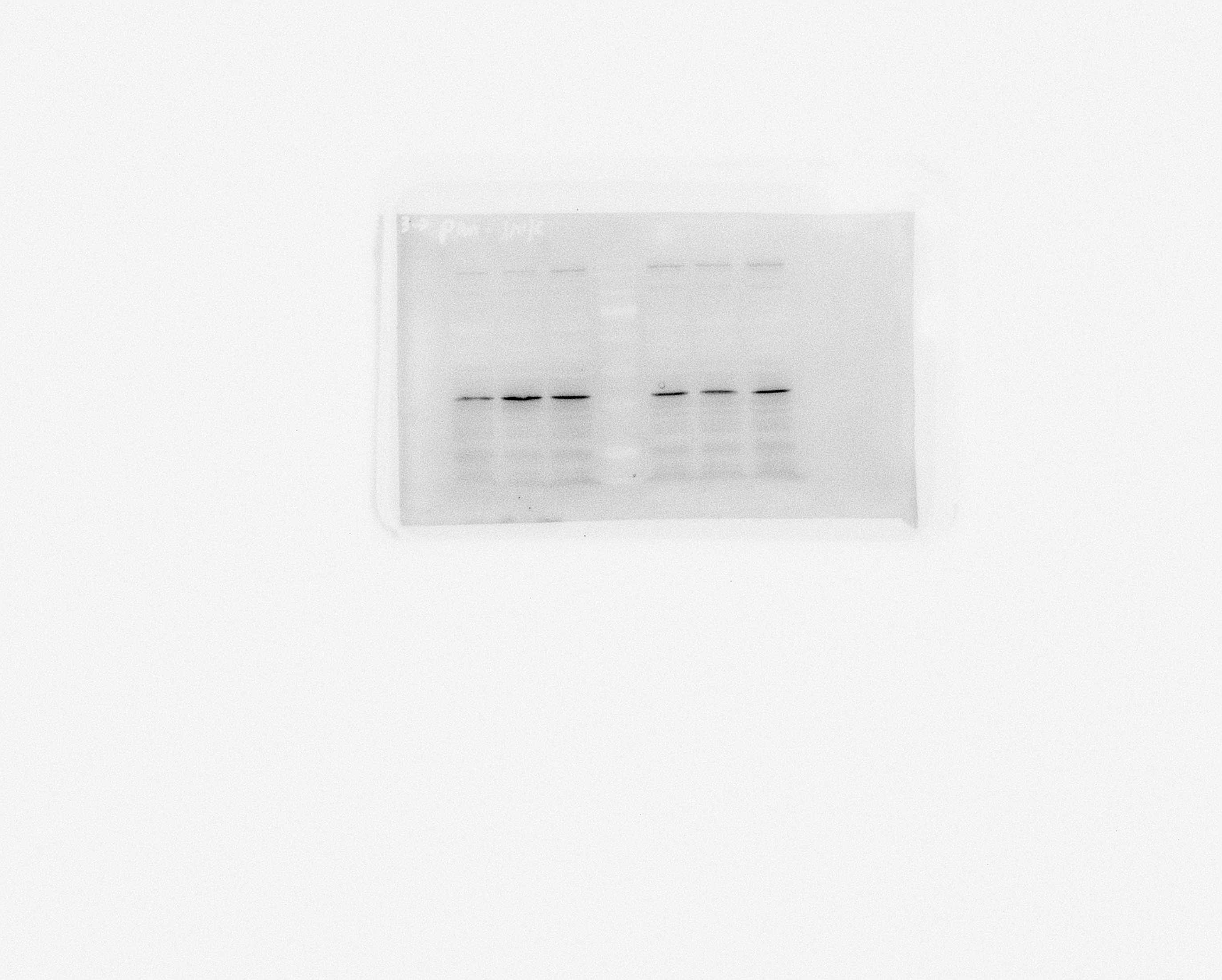

Supplement: Figure 6—source data 2. [file elife-77340-fig6-data2.zip › Figure 6D raw data/#2#3/total JNK/total JNK.tif]

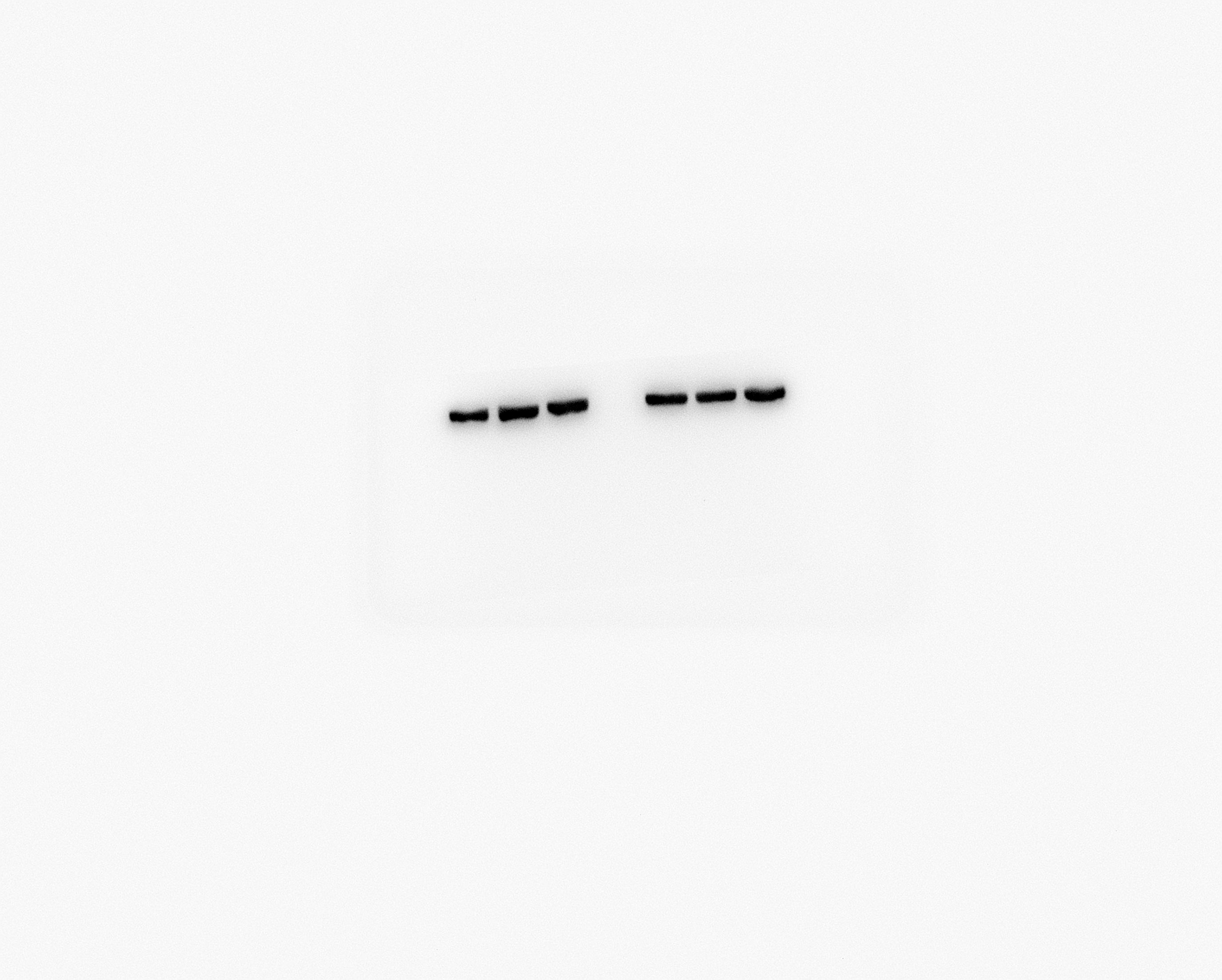

Supplement: Figure 6—source data 2. [file elife-77340-fig6-data2.zip › Figure 6D raw data/#2#3/tubulin/tubulin.tif]

#1

myc

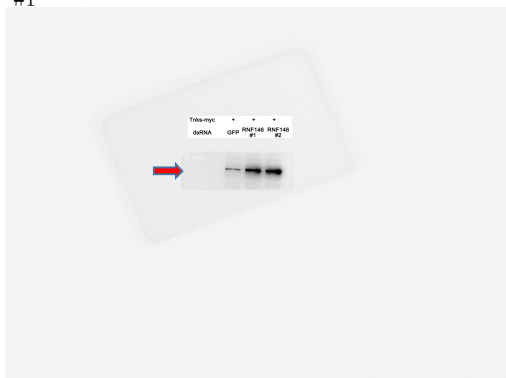

#2&#3

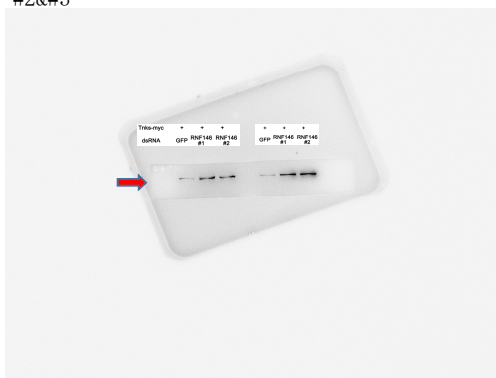

P-JNK

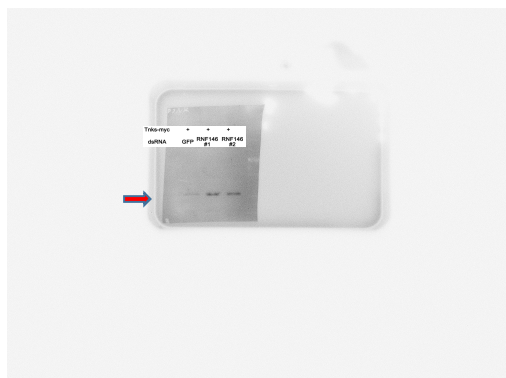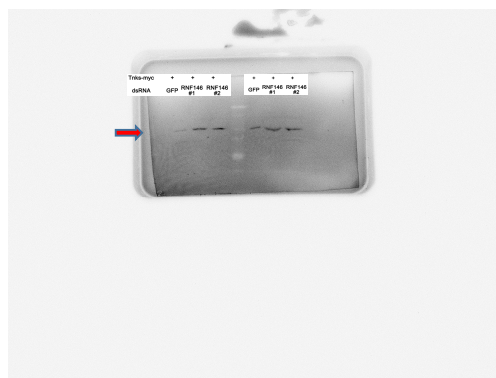

JNK

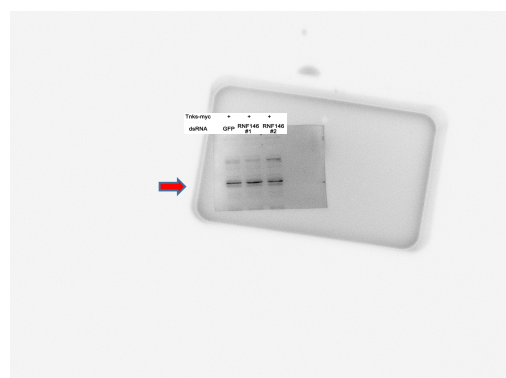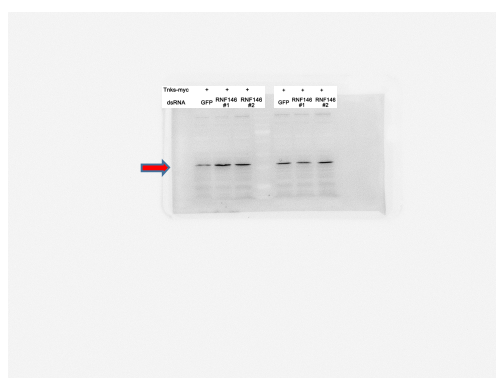

tubulin

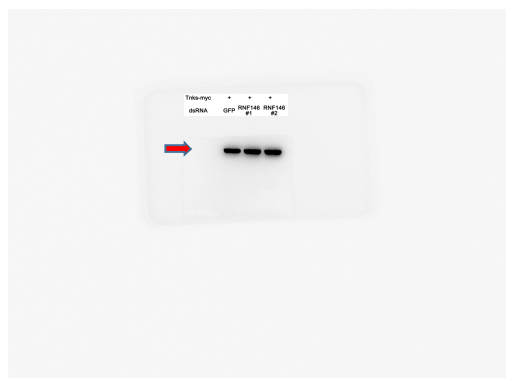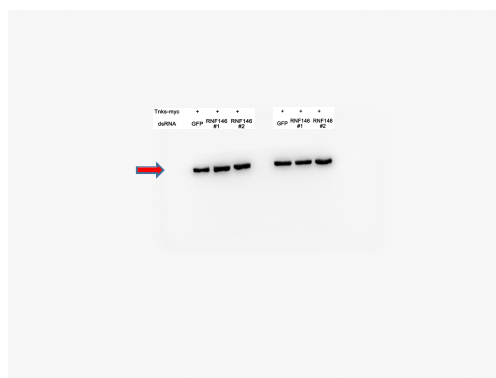

Supplement: Figure 6—source data 2. [file elife-77340-fig6-data2.zip › Figure 6D uncropped blots with label/Figure 6D with label 2.pdf]

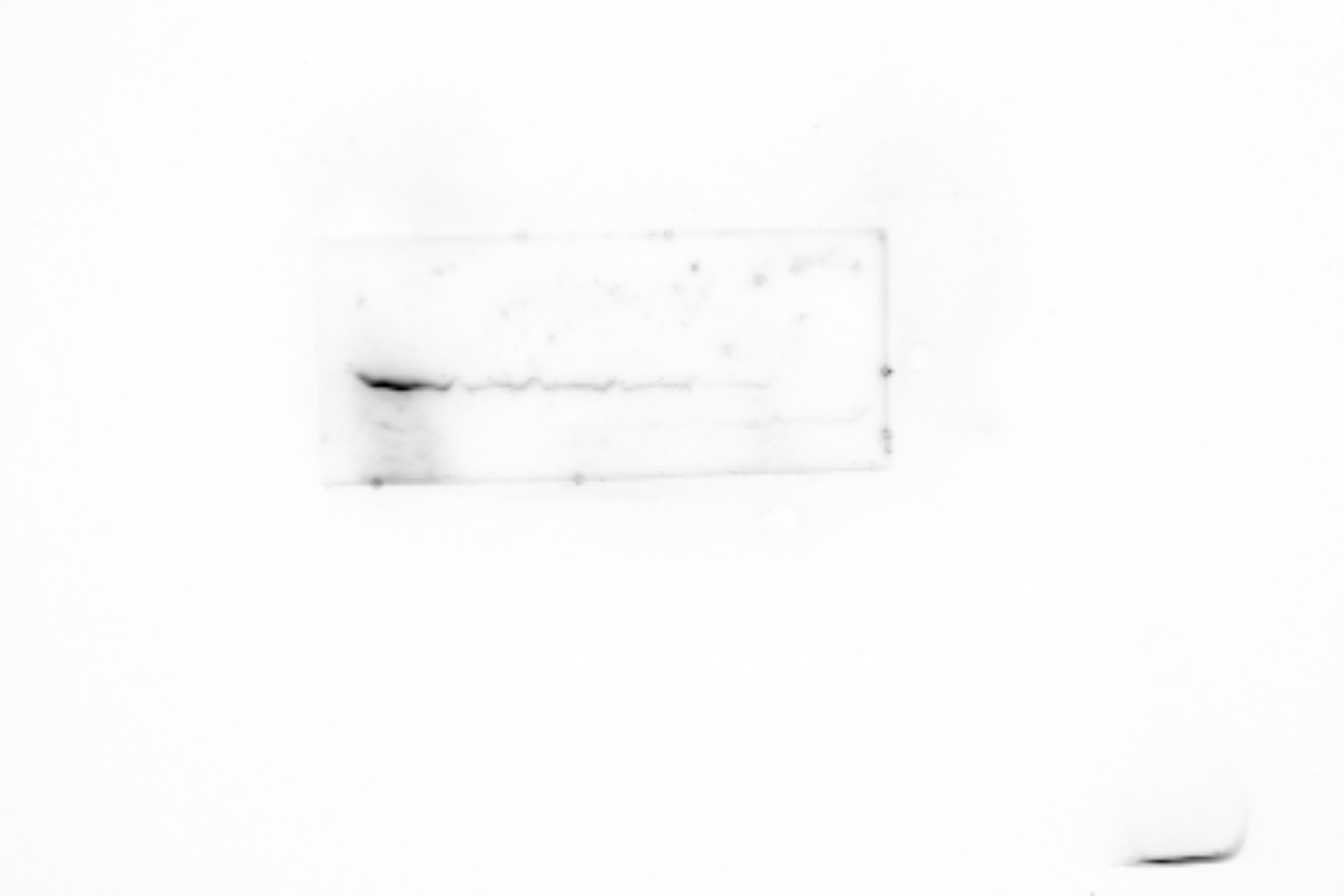

Supplement: Figure 6—source data 2. [file elife-77340-fig6-data2.zip › Figure 6N raw data/#1/myc/Figure 6N myc #1.tif]

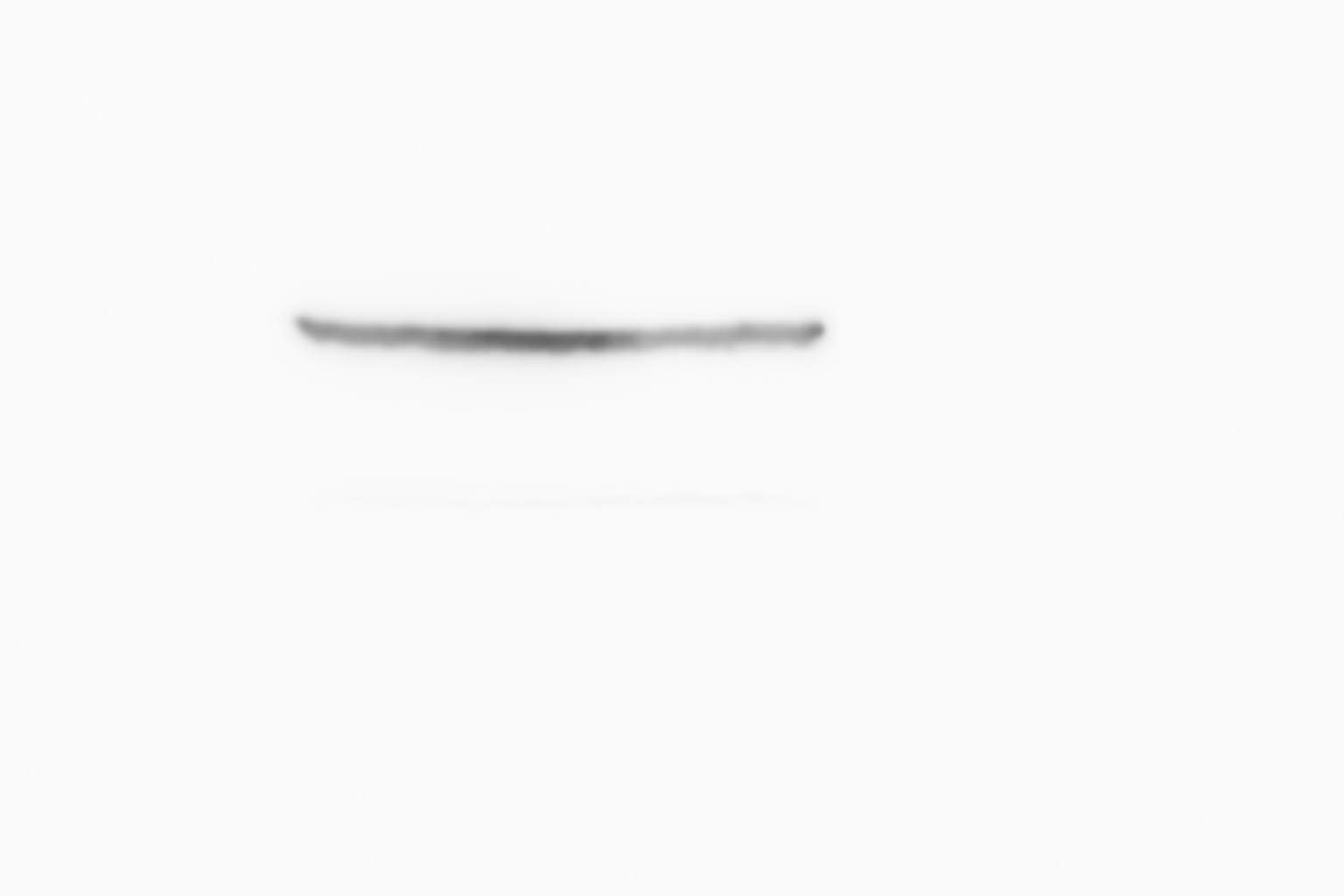

Supplement: Figure 6—source data 2. [file elife-77340-fig6-data2.zip › Figure 6N raw data/#1/Tubulin/Figure 6N tubulin #1.tif]

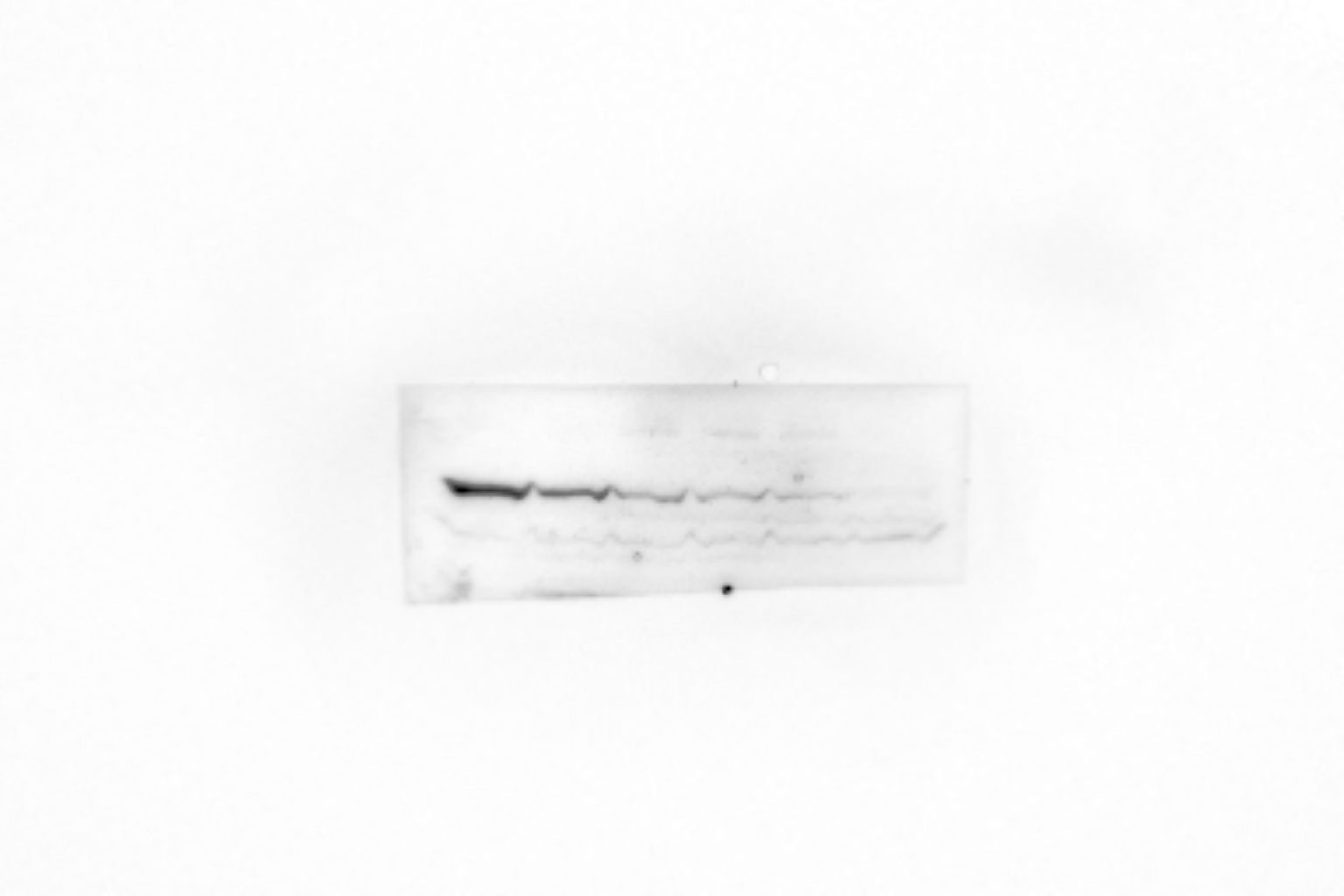

Supplement: Figure 6—source data 2. [file elife-77340-fig6-data2.zip › Figure 6N raw data/#2/myc/Figure 6N myc #2.tif]

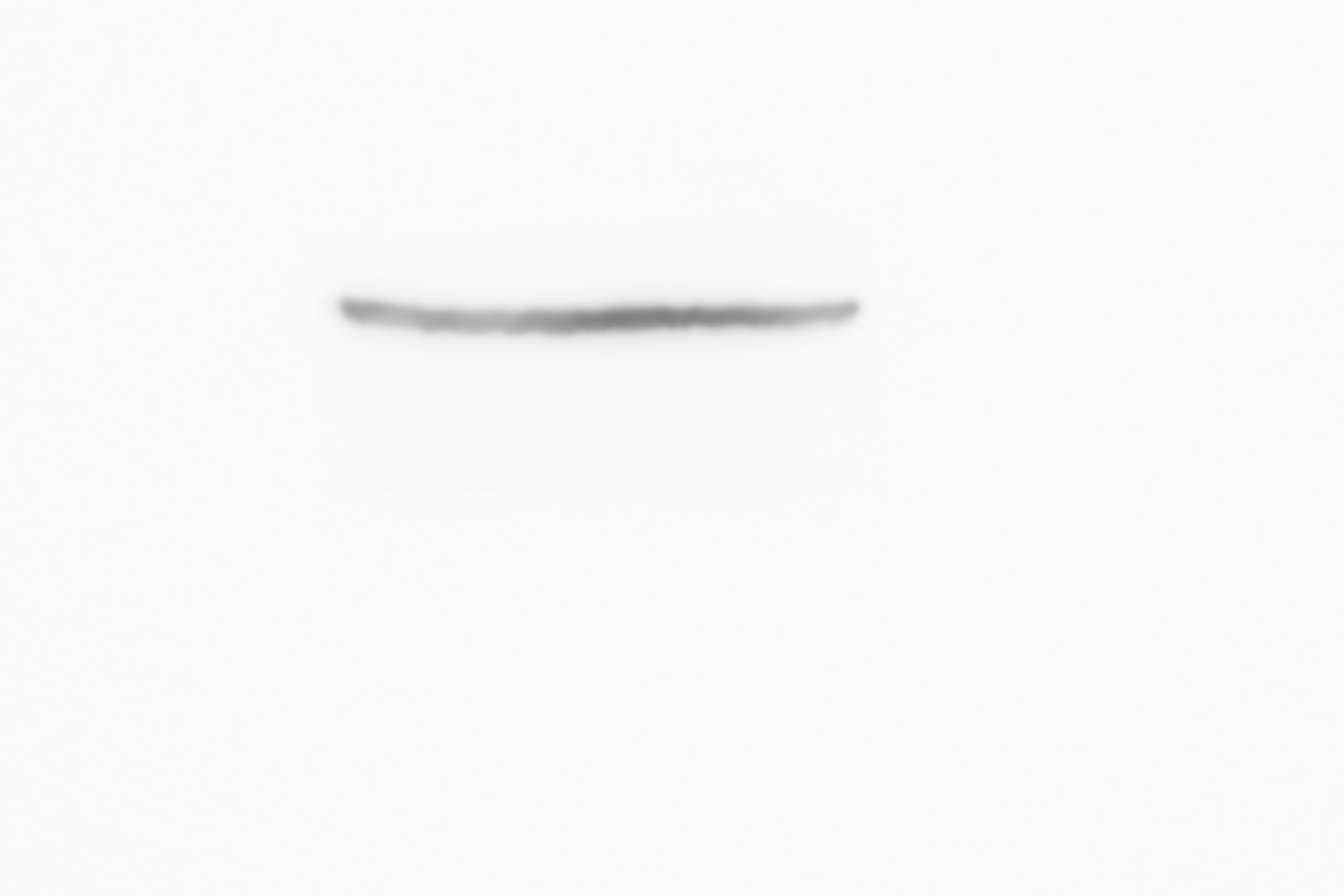

Supplement: Figure 6—source data 2. [file elife-77340-fig6-data2.zip › Figure 6N raw data/#2/tubulin/Figure 6N tubulin #2.tif]

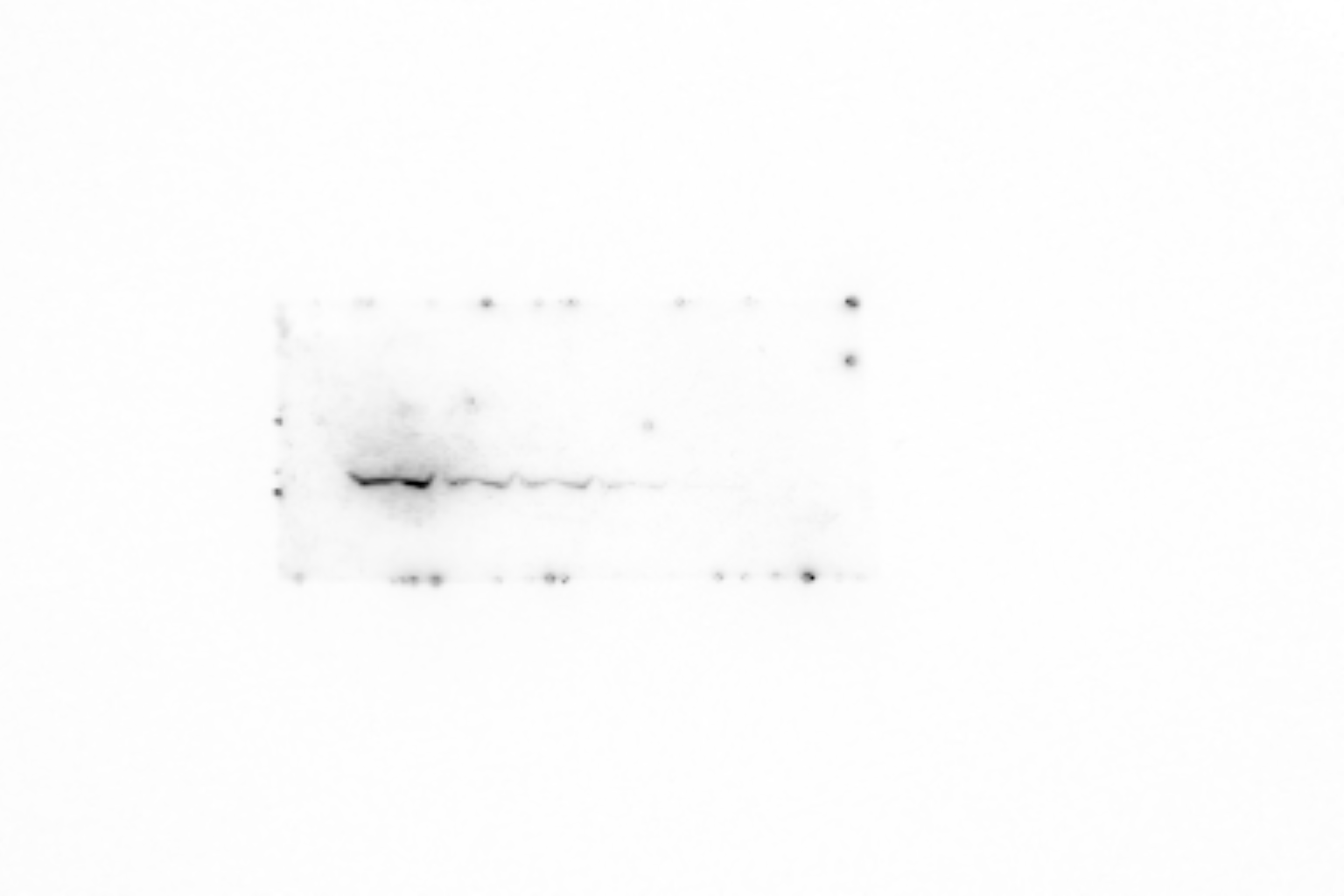

Supplement: Figure 6—source data 2. [file elife-77340-fig6-data2.zip › Figure 6N raw data/#3/myc/Figure 6N myc #3.tif]

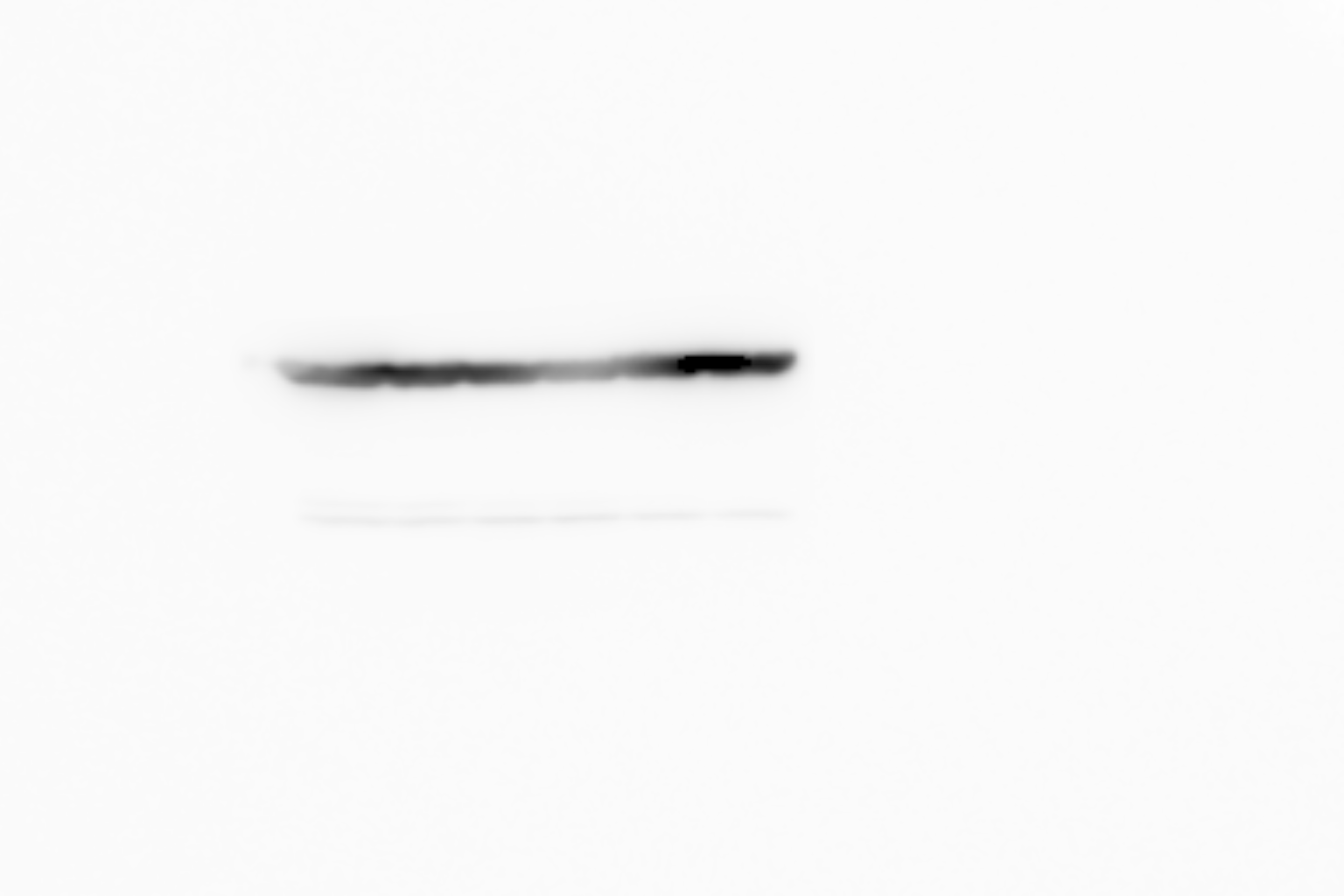

Supplement: Figure 6—source data 2. [file elife-77340-fig6-data2.zip › Figure 6N raw data/#3/tubulin/Figure 6N tubulin #3.tif]

Figure 6N

anti-myc

anti-Tubulin

#1

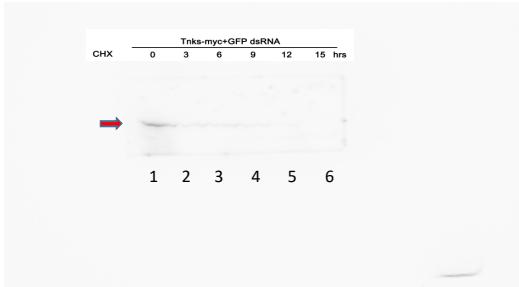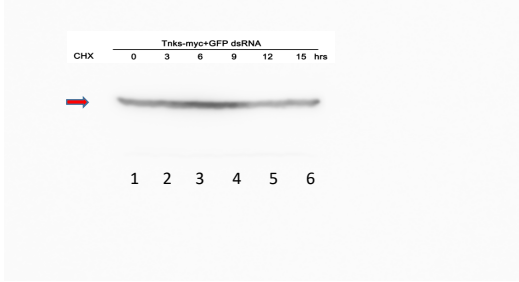

#2

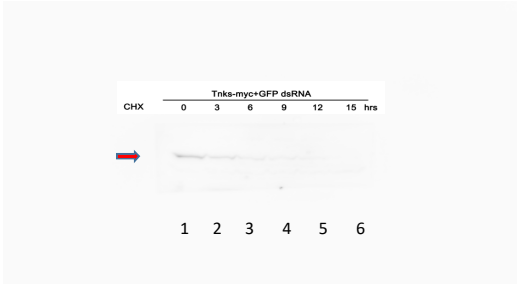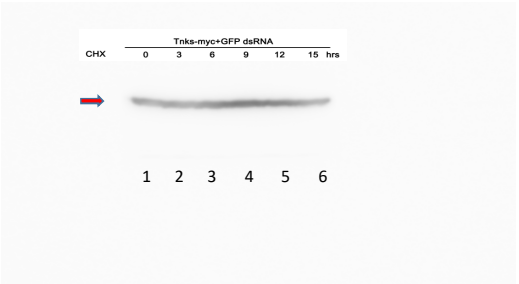

#3

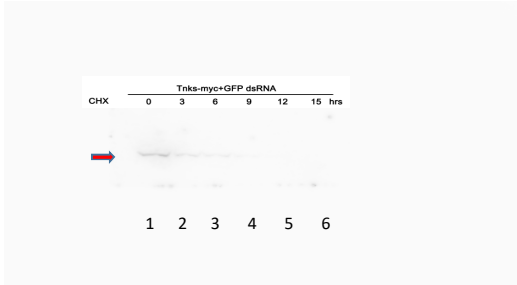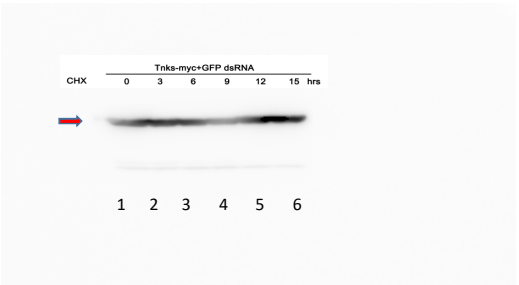

Supplement: Figure 6—source data 2. [file elife-77340-fig6-data2.zip › Figure 6N uncropped blots with label/Figure 6N with label.pdf]

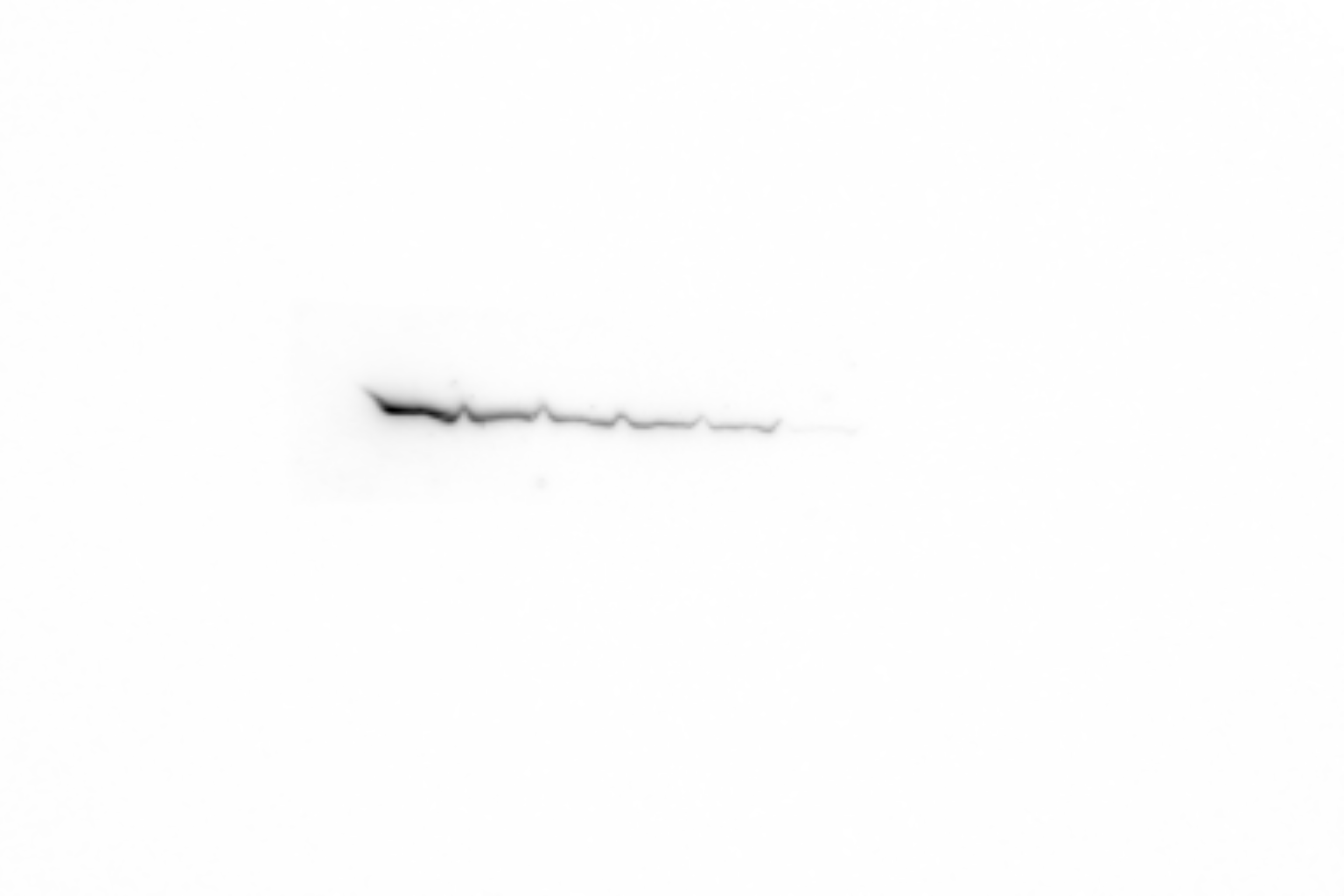

Supplement: Figure 6—source data 2. [file elife-77340-fig6-data2.zip › Figure 6O raw data/#1/myc/Figure 6O myc #1.tif]

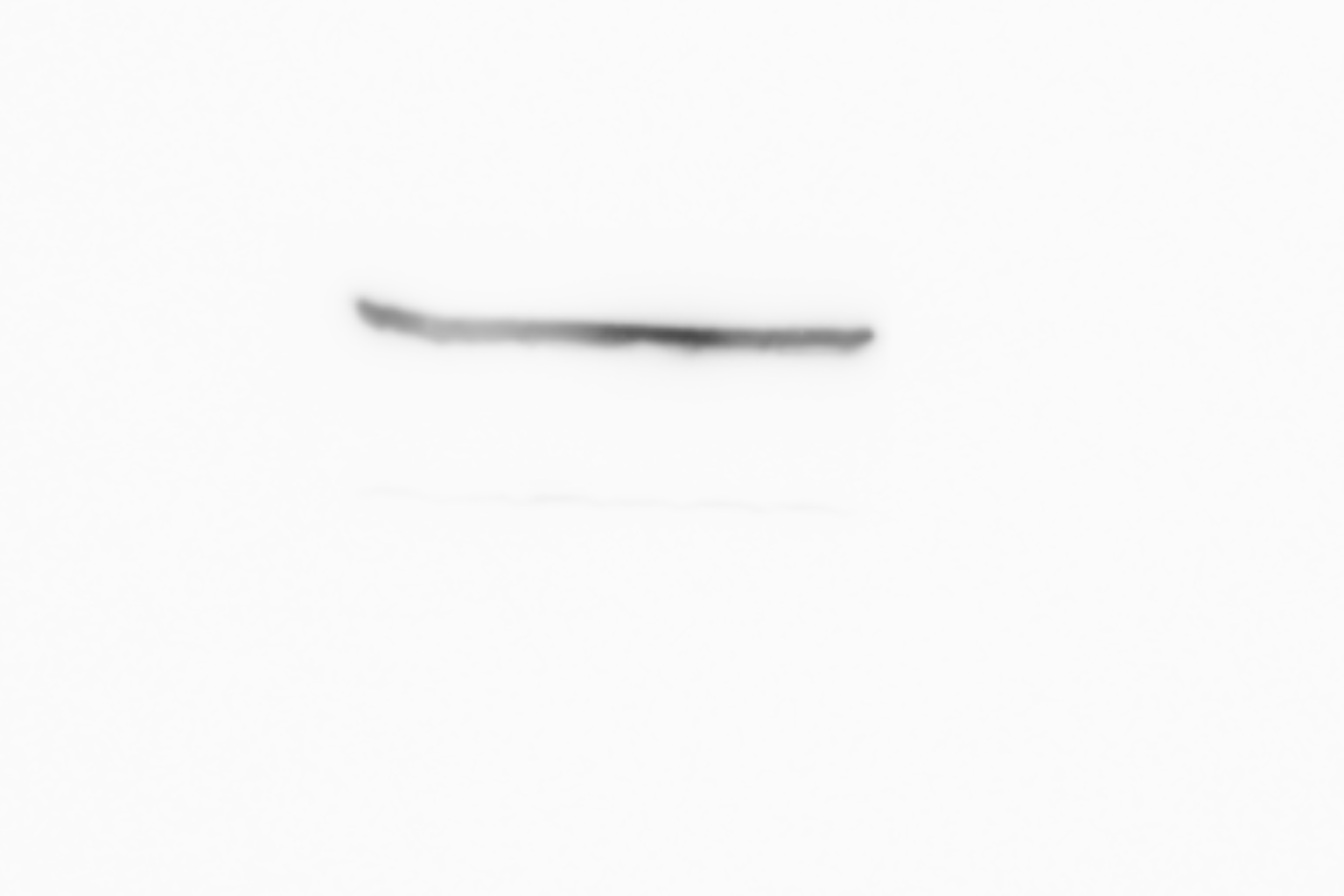

Supplement: Figure 6—source data 2. [file elife-77340-fig6-data2.zip › Figure 6O raw data/#1/Tubulin/Figure 6O tubulin #1.tif]

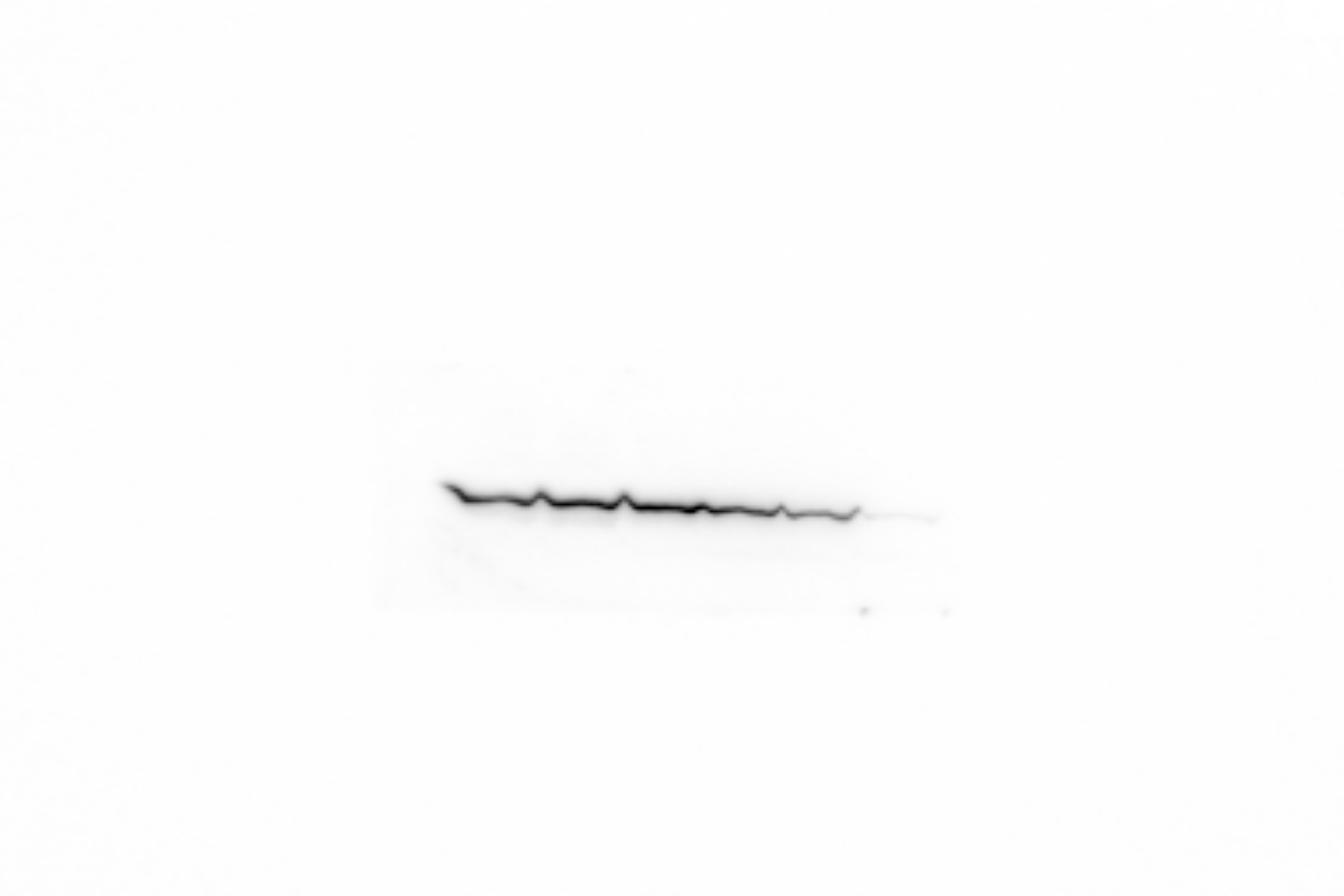

Supplement: Figure 6—source data 2. [file elife-77340-fig6-data2.zip › Figure 6O raw data/#2/myc/Figure 6O myc #2.tif]

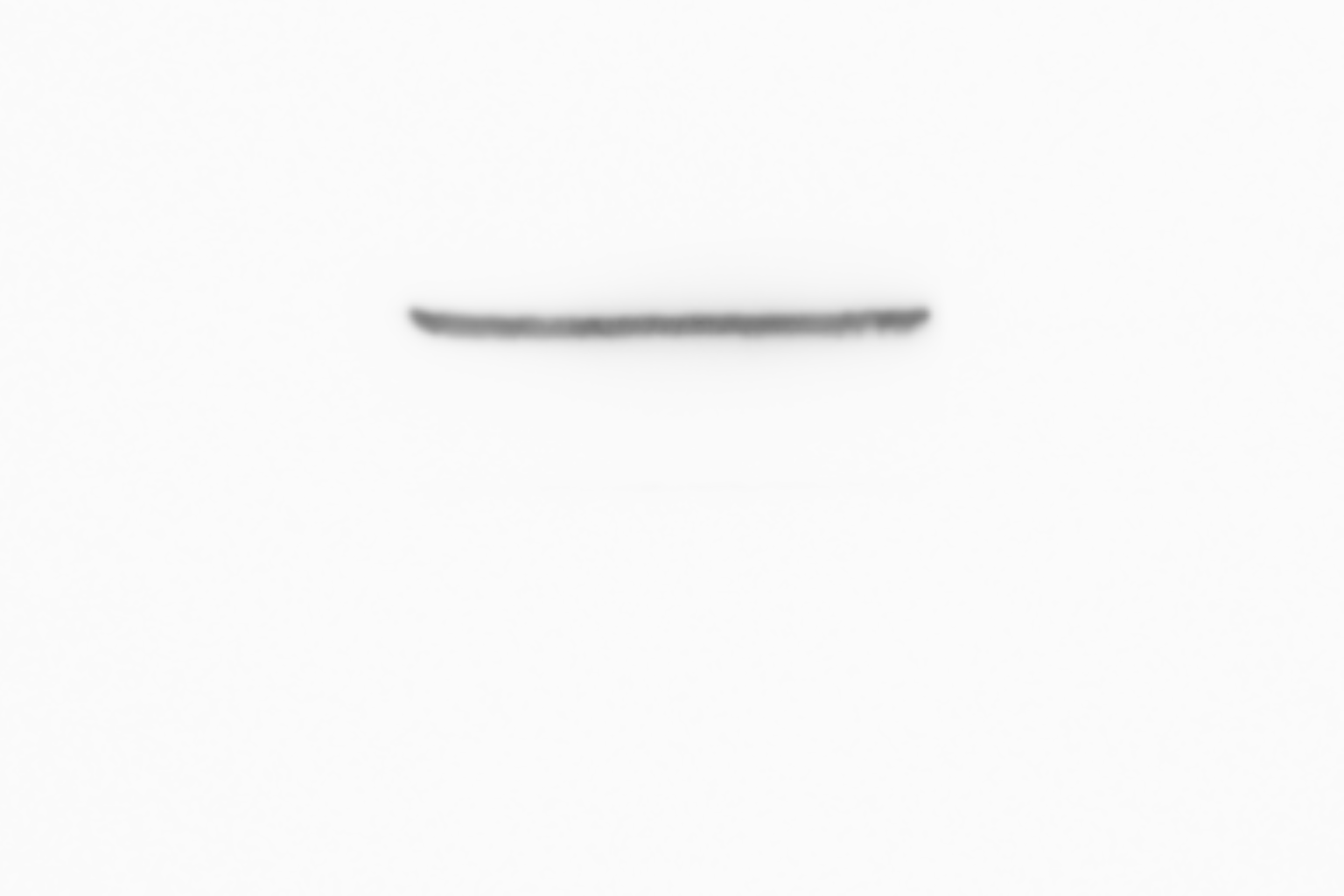

Supplement: Figure 6—source data 2. [file elife-77340-fig6-data2.zip › Figure 6O raw data/#2/tubulin/Figure 6O tubulin #2.tif]

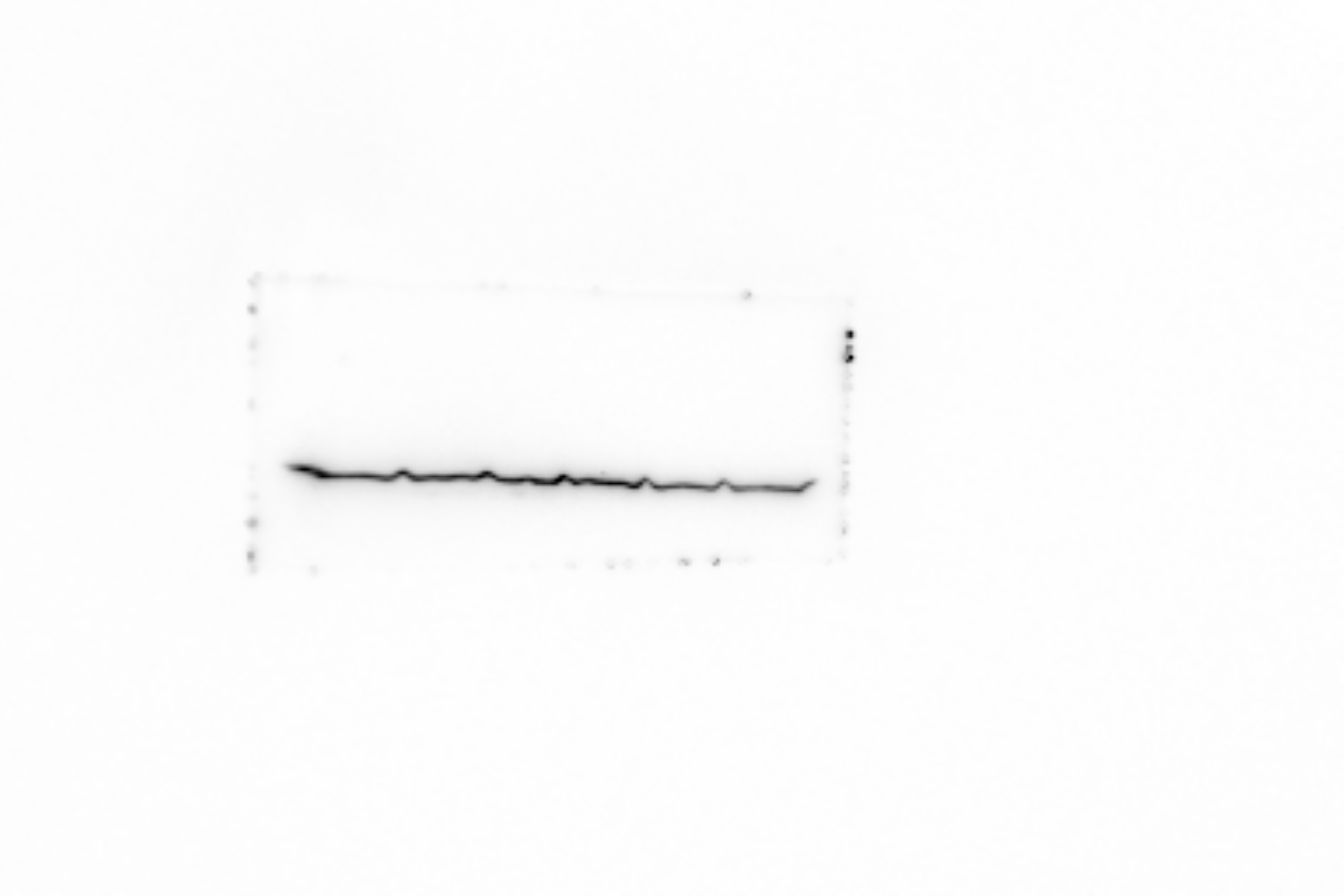

Supplement: Figure 6—source data 2. [file elife-77340-fig6-data2.zip › Figure 6O raw data/#3/myc/Figure 6O myc #3.tif]

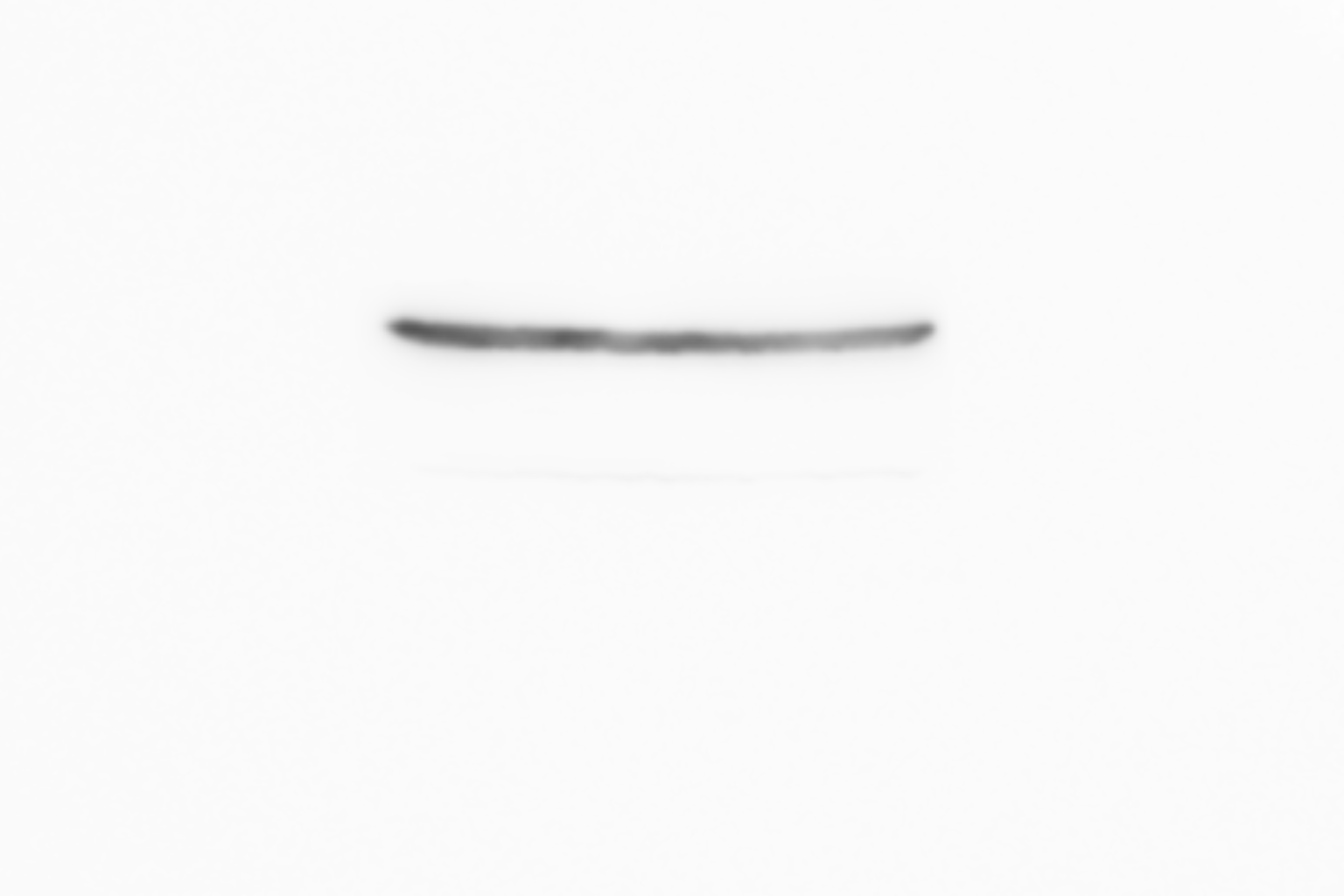

Supplement: Figure 6—source data 2. [file elife-77340-fig6-data2.zip › Figure 6O raw data/#3/tubulin/Figure 6O tubulin #3.tif]

Figure 60

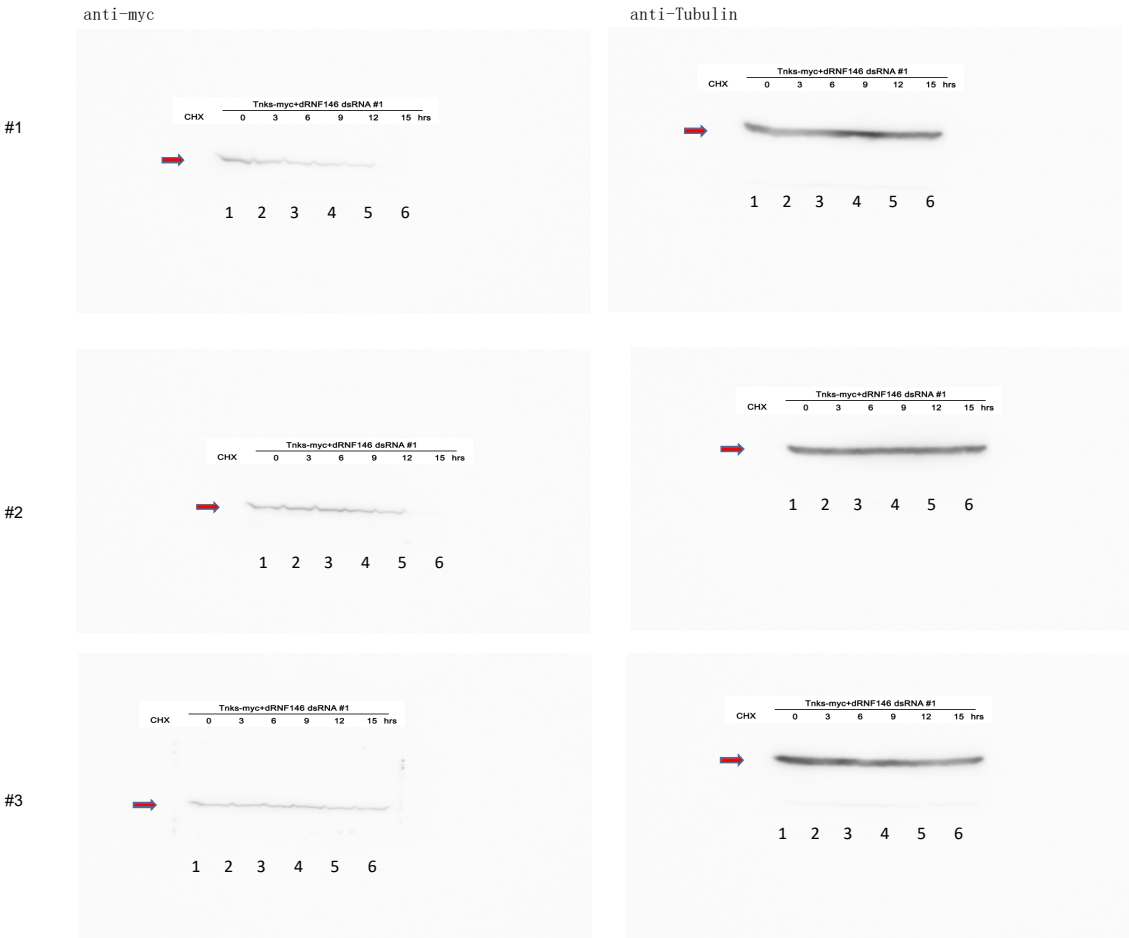

Supplement: Figure 6—source data 2. [file elife-77340-fig6-data2.zip › Figure 6O uncropped blots with label/Figure 6O with label.pdf]

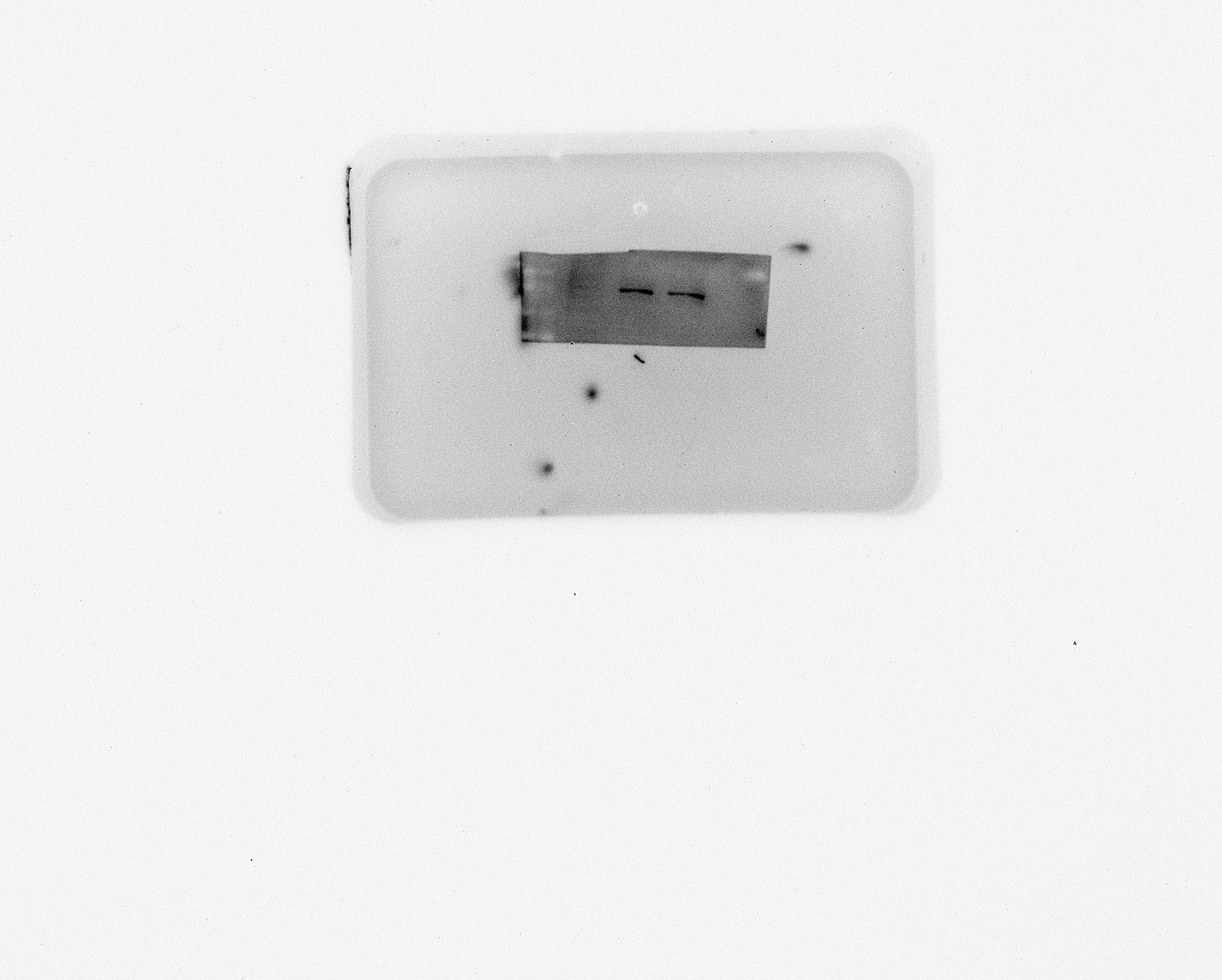

Supplement: Figure 6—figure supplement 1—source data 1. [file elife-77340-fig6-figsupp1-data1.zip › Figure 6—figure supplement 1—source data 1/Figure 6—figure supplement 1A raw data/#1/myc/myc.tif]

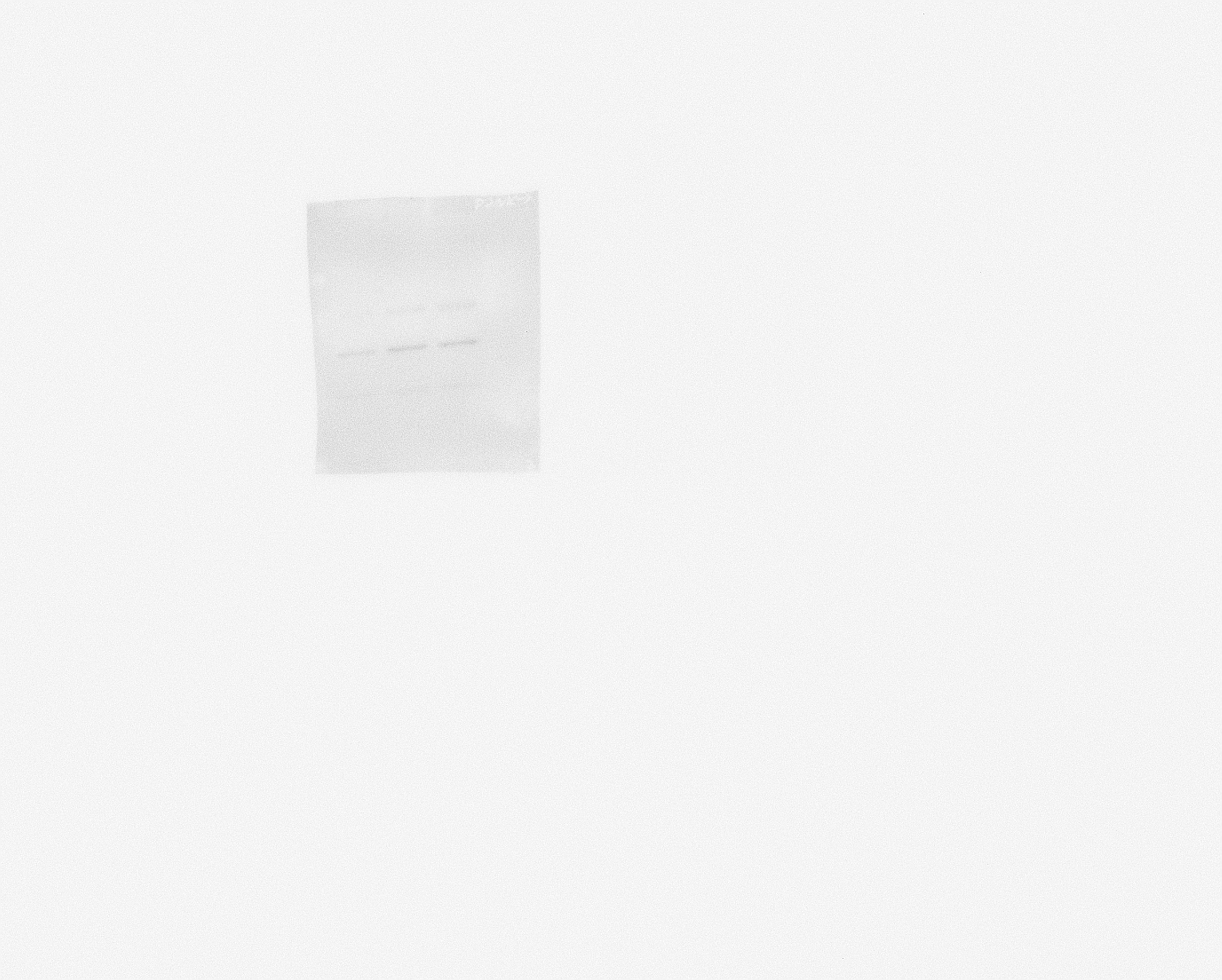

Supplement: Figure 6—figure supplement 1—source data 1. [file elife-77340-fig6-figsupp1-data1.zip › Figure 6—figure supplement 1—source data 1/Figure 6—figure supplement 1A raw data/#1/P-JNK/P-JNK.tif]

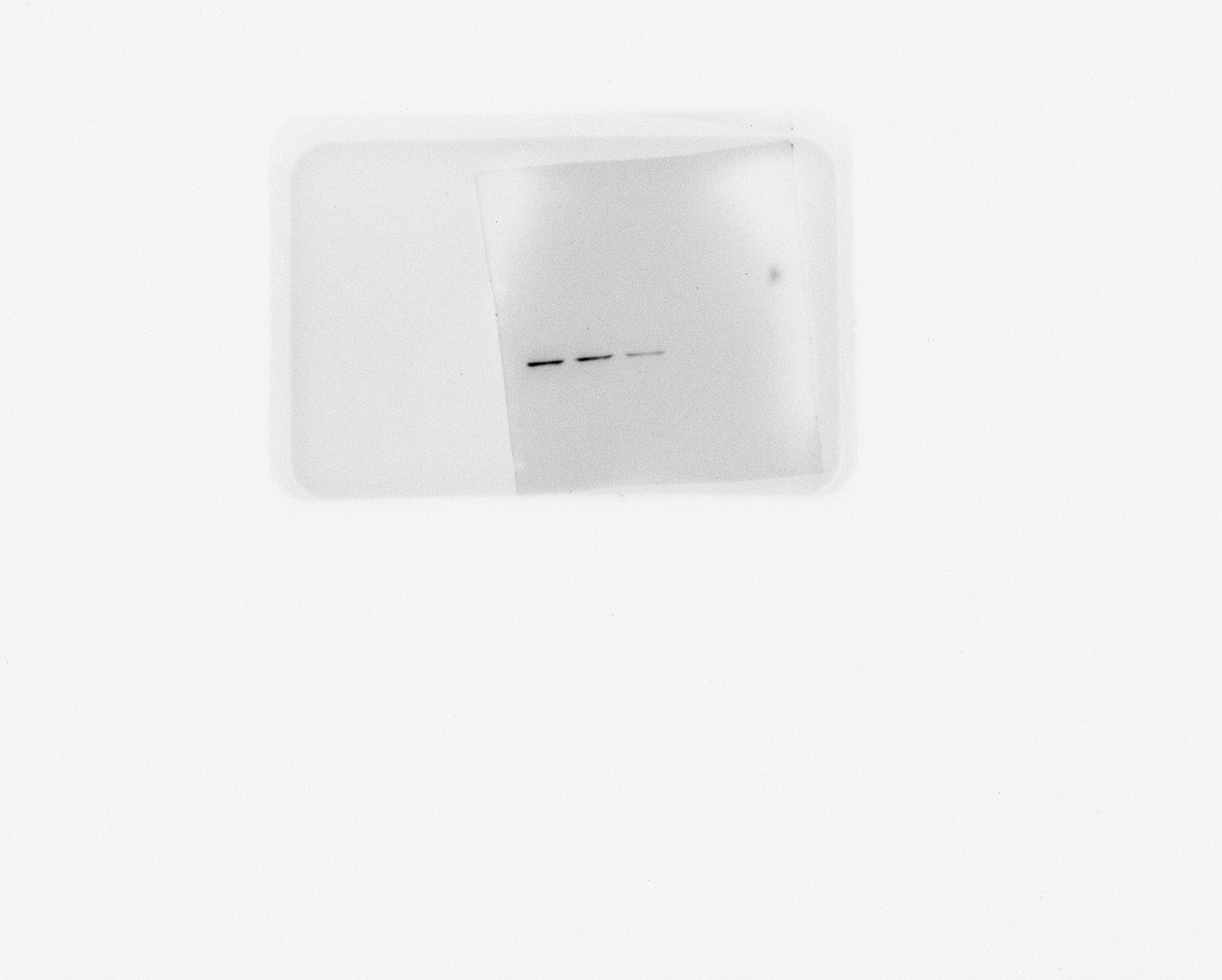

Supplement: Figure 6—figure supplement 1—source data 1. [file elife-77340-fig6-figsupp1-data1.zip › Figure 6—figure supplement 1—source data 1/Figure 6—figure supplement 1A raw data/#1/total JNK/JNK.tif]

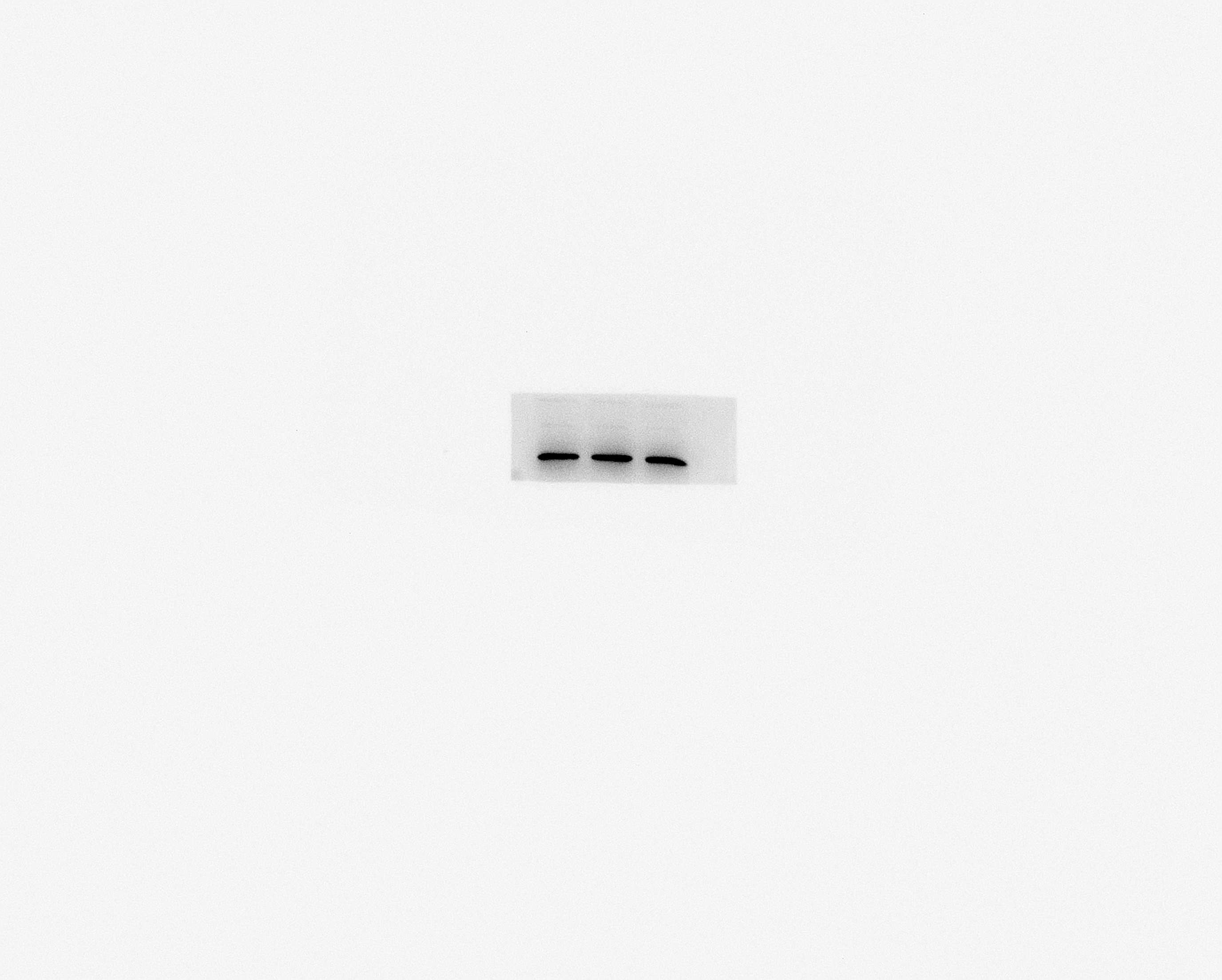

Supplement: Figure 6—figure supplement 1—source data 1. [file elife-77340-fig6-figsupp1-data1.zip › Figure 6—figure supplement 1—source data 1/Figure 6—figure supplement 1A raw data/#1/tubulin/tubulin.tif]

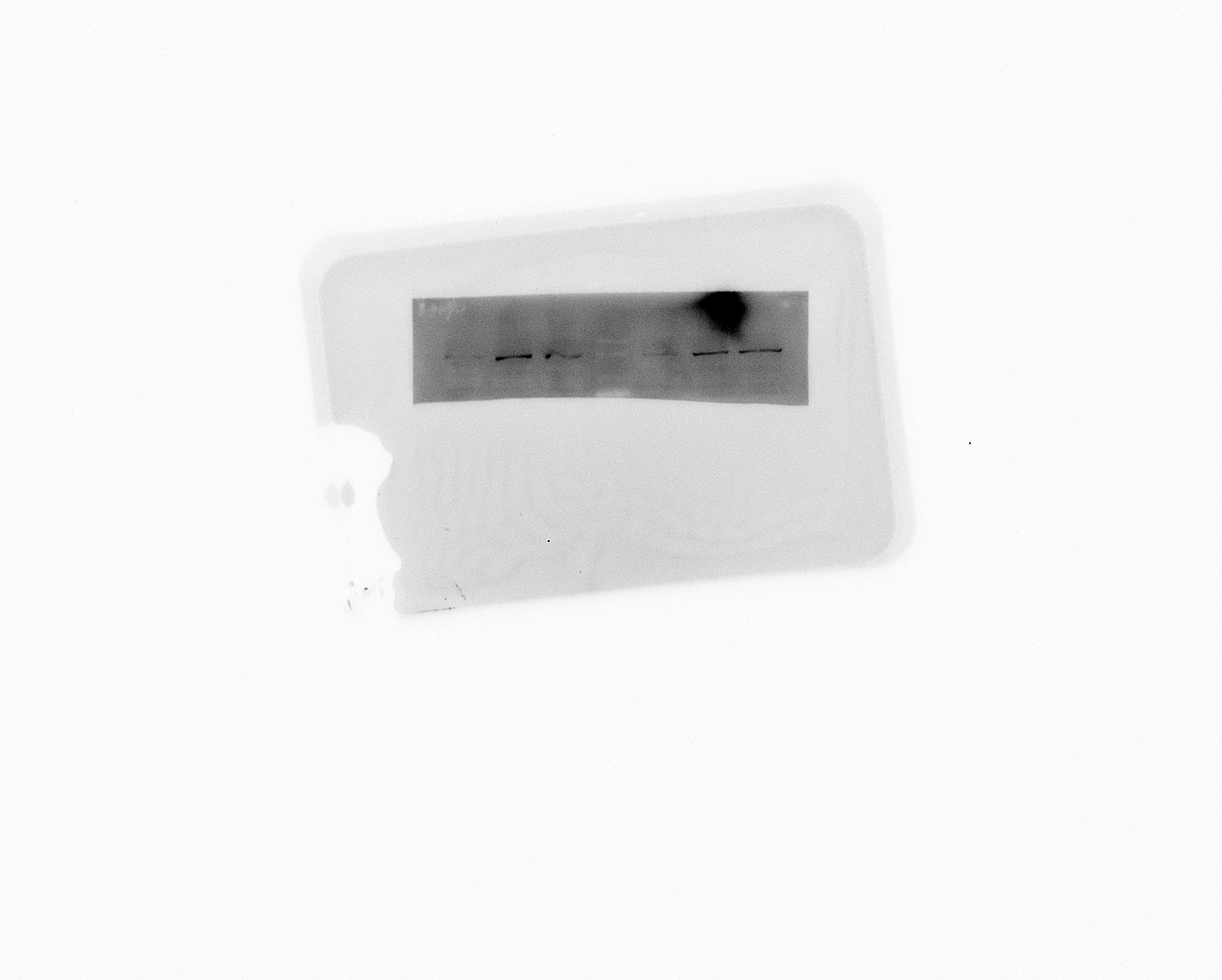

Supplement: Figure 6—figure supplement 1—source data 1. [file elife-77340-fig6-figsupp1-data1.zip › Figure 6—figure supplement 1—source data 1/Figure 6—figure supplement 1A raw data/#2#3/myc/myc.tif]

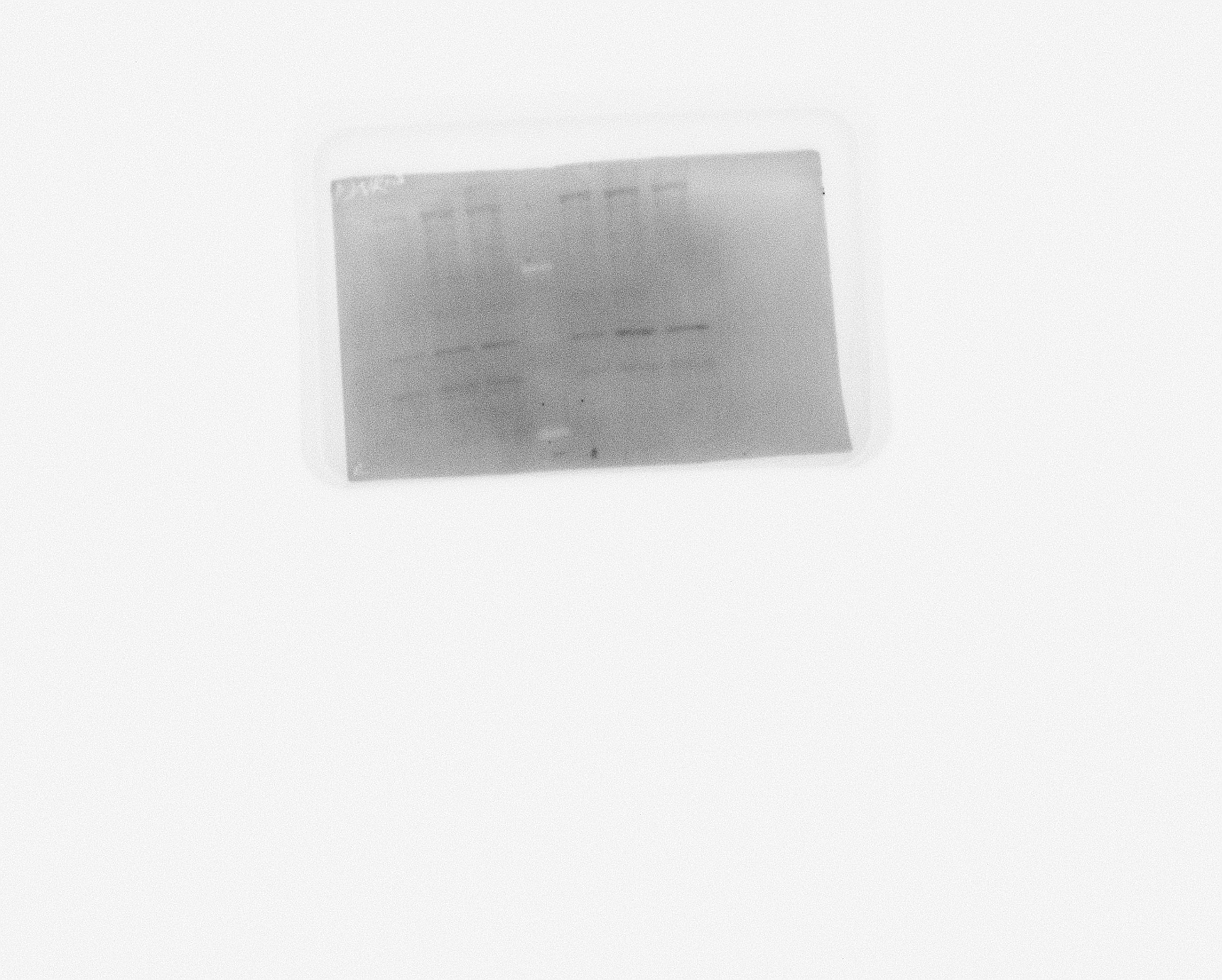

Supplement: Figure 6—figure supplement 1—source data 1. [file elife-77340-fig6-figsupp1-data1.zip › Figure 6—figure supplement 1—source data 1/Figure 6—figure supplement 1A raw data/#2#3/P-JNK/P-JNK.tif]

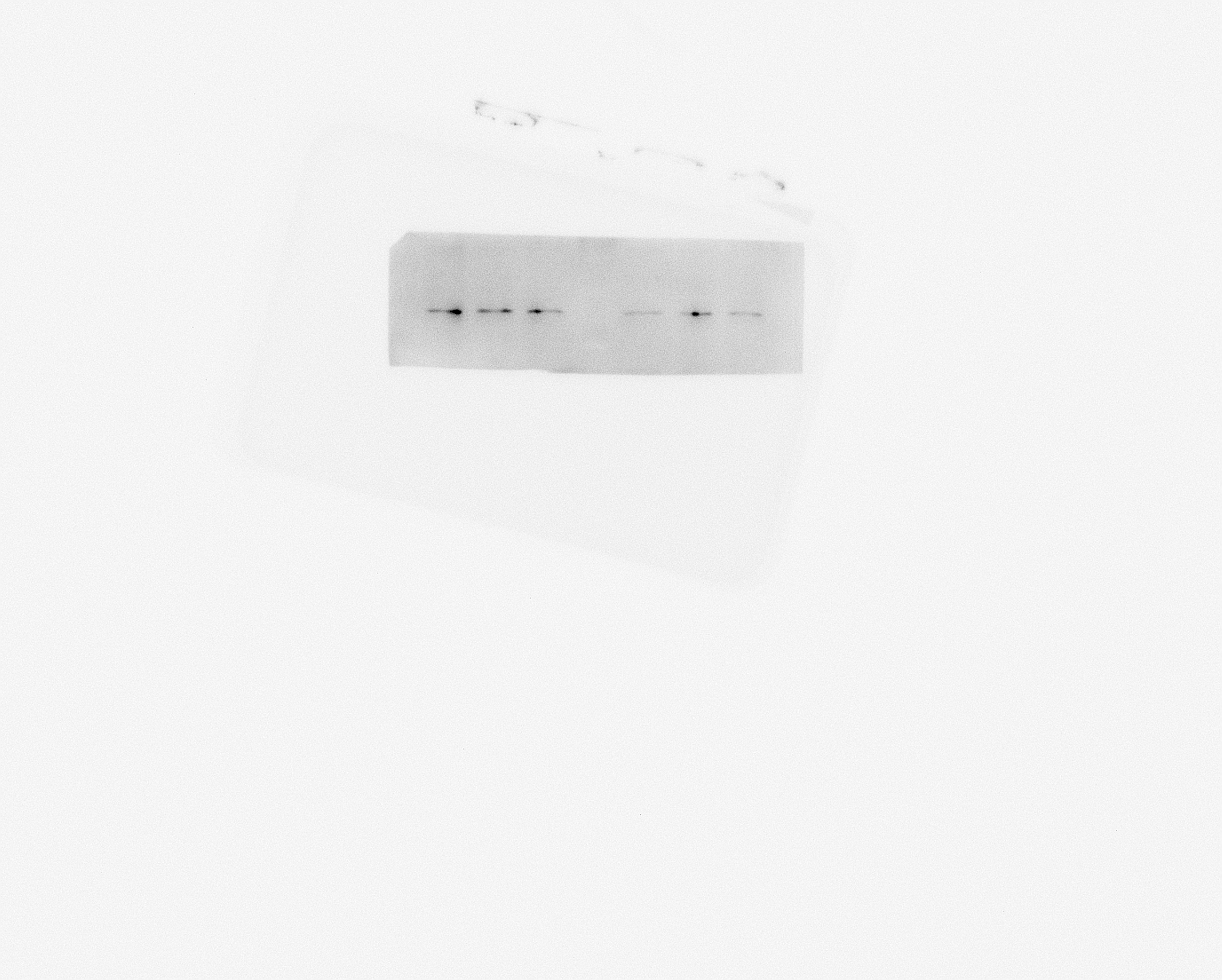

Supplement: Figure 6—figure supplement 1—source data 1. [file elife-77340-fig6-figsupp1-data1.zip › Figure 6—figure supplement 1—source data 1/Figure 6—figure supplement 1A raw data/#2#3/total JNK/JNK.tif]

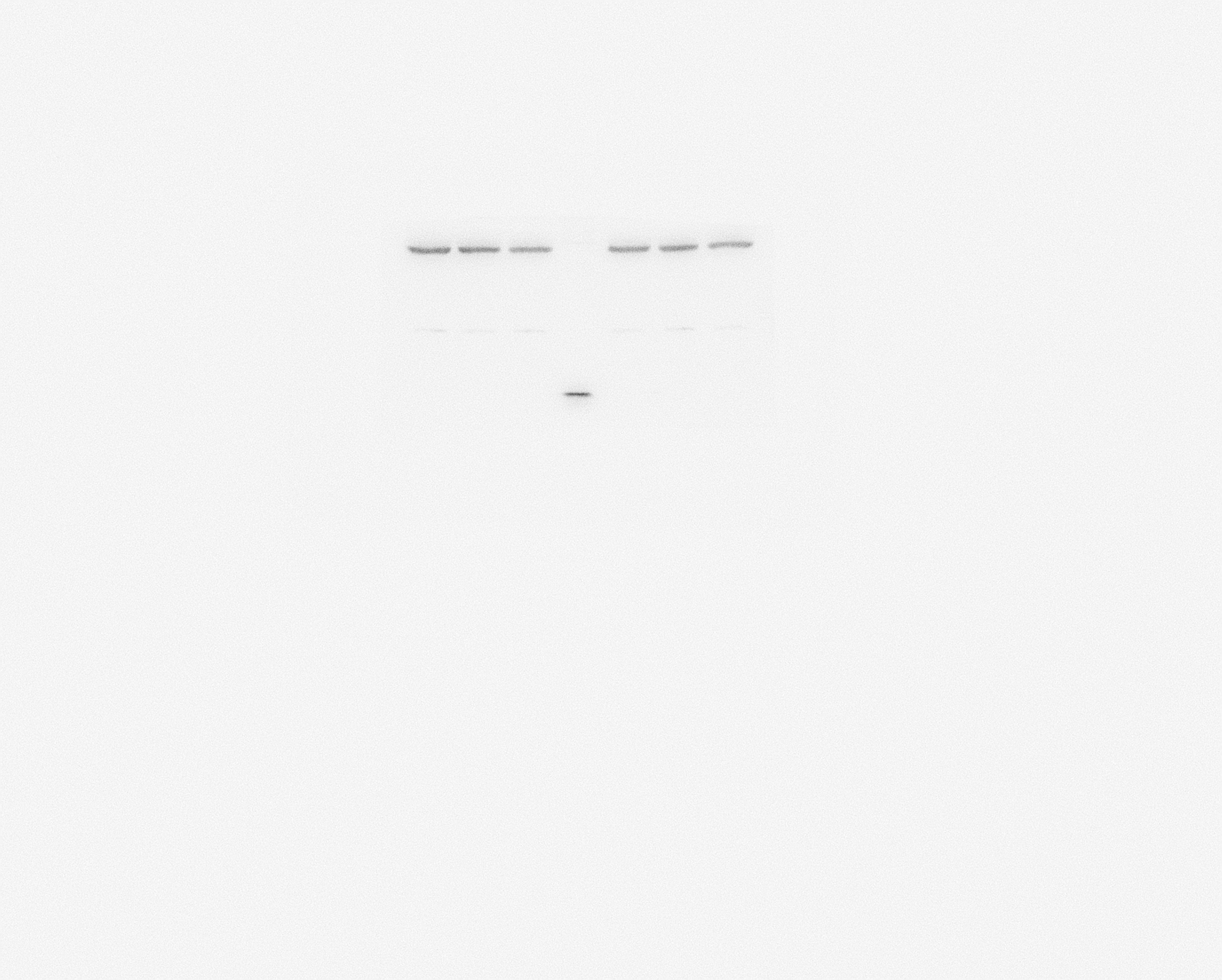

Supplement: Figure 6—figure supplement 1—source data 1. [file elife-77340-fig6-figsupp1-data1.zip › Figure 6—figure supplement 1—source data 1/Figure 6—figure supplement 1A raw data/#2#3/tubulin/tubulin.tif]

#1

myc

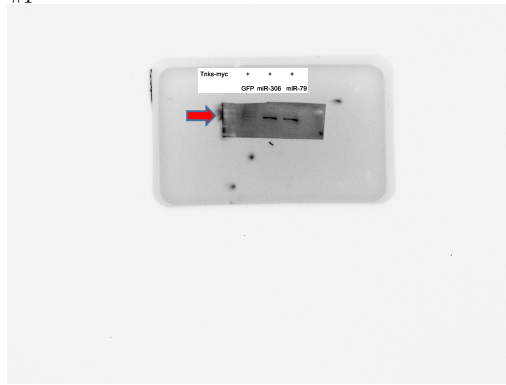

#2&#3

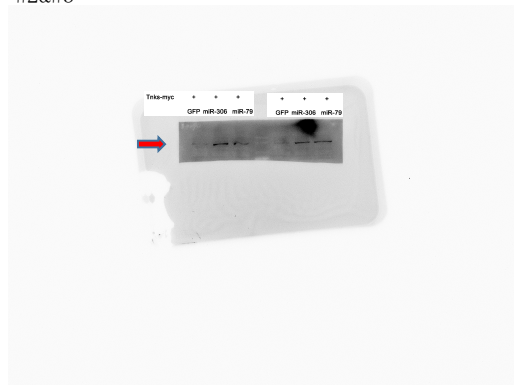

P-JNK

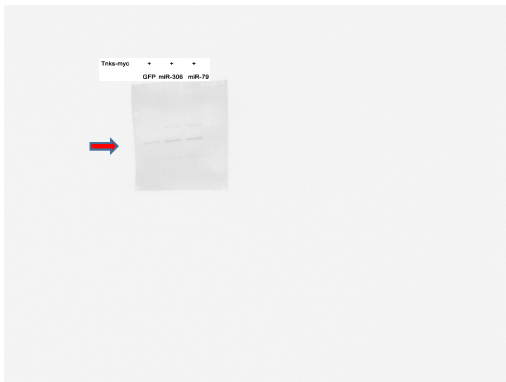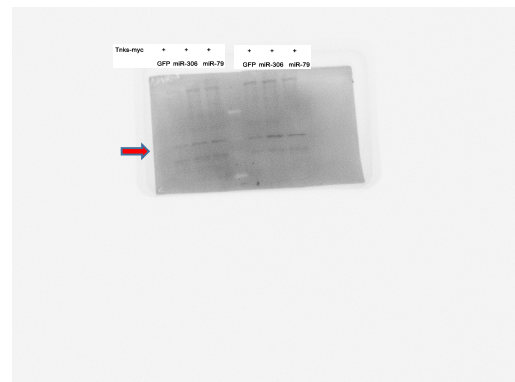

JNK

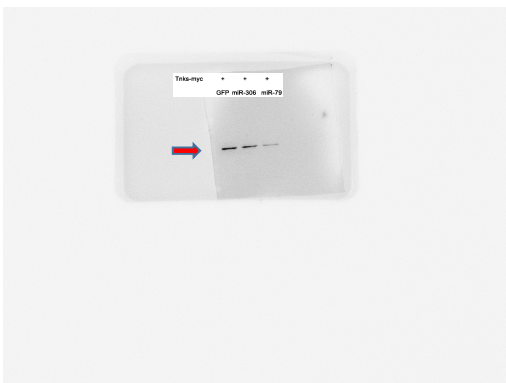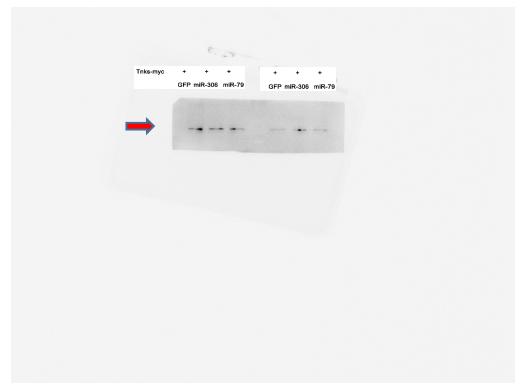

tubulin

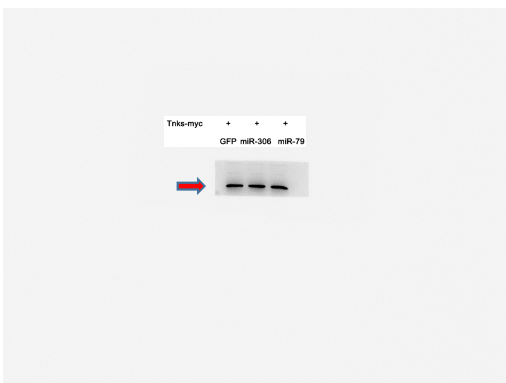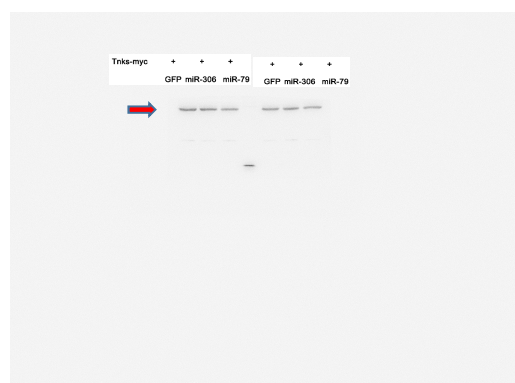

Supplement: Figure 6—figure supplement 1—source data 1. [file elife-77340-fig6-figsupp1-data1.zip › Figure 6—figure supplement 1—source data 1/Figure 6—figure supplement 1A uncropped blots with label/Figure 6—figure supplement 1A with label.pdf]
